# Phospholipid imbalance impairs autophagosome completion

**DOI:** 10.15252/embj.2022110771

**Published:** 2022-10-27

**Authors:** Alexandra Polyansky, Oren Shatz, Milana Fraiberg, Eyal Shimoni, Tali Dadosh, Muriel Mari, Fulvio M Reggiori, Chao Qin, Xianlin Han, Zvulun Elazar

**Affiliations:** ^1^ Department of Biomolecular Sciences The Weizmann Institute of Science Rehovot Israel; ^2^ Department of Chemical Research Support The Weizmann Institute of Science Rehovot Israel; ^3^ Department of Biomedical Sciences of Cells and Systems University of Groningen, University Medical Center Groningen Groningen The Netherlands; ^4^ Department of Biomedicine Aarhus University Aarhus Denmark; ^5^ Barshop Institute for Longevity and Aging Studies University of Texas Health Science Center at San Antonio San Antonio TX USA; ^6^ Department of Medicine University of Texas Health Science Center at San Antonio San Antonio TX USA

**Keywords:** autophagosome biogenesis, autophagy, phagophore, phospholipids, Autophagy & Cell Death

## Abstract

Autophagy, a conserved eukaryotic intracellular catabolic pathway, maintains cell homeostasis by lysosomal degradation of cytosolic material engulfed in double membrane vesicles termed autophagosomes, which form upon sealing of single‐membrane cisternae called phagophores. While the role of phosphatidylinositol 3‐phosphate (PI3P) and phosphatidylethanolamine (PE) in autophagosome biogenesis is well‐studied, the roles of other phospholipids in autophagy remain rather obscure. Here we utilized budding yeast to study the contribution of phosphatidylcholine (PC) to autophagy. We reveal for the first time that genetic loss of PC biosynthesis via the CDP‐DAG pathway leads to changes in lipid composition of autophagic membranes, specifically replacement of PC by phosphatidylserine (PS). This impairs closure of the autophagic membrane and autophagic flux. Consequently, we show that choline‐dependent recovery of *de novo* PC biosynthesis via the CDP‐choline pathway restores autophagosome formation and autophagic flux in PC‐deficient cells. Our findings therefore implicate phospholipid metabolism in autophagosome biogenesis.

## Graphical abstract

A relatively high ratio of phosphatidylcholine to phosphatidylserine drives closure of the autophagosome membrane.

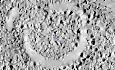

## Introduction

Macroautophagy (hereafter termed autophagy) is an evolutionarily conserved intracellular trafficking pathway that degrades cytosolic components, including protein aggregates, damaged or excess organelles and bulk cytosol, thereby contributing to the recycling of the macromolecular building blocks and cell homeostasis. Autophagy is triggered by stresses such as nutrient deprivation and other metabolic conditions (Weidberg *et al*, 2011), and defects in this process have been implicated in several pathophysiological conditions (Dikic & Elazar, 2018).

Autophagy is initiated with hierarchical recruitment of autophagy‐related (Atg) proteins to the phagophore assembly site (PAS; Suzuki *et al*, 2007). Activation of the Atg1 complex kinase activity, followed by recruitment of Atg9 vesicles and the phosphatidylinositol 3‐kinase complex that generates phosphatidylinositol‐3‐phosphate (PI3P), leads to formation of a single‐membrane cistern, termed phagophore. Following expansion of the phagophore into a cup shape structure, its edge seals to form a double‐membrane vesicle, called autophagosome. Complete autophagosomes undergo maturation, namely the cytoplasmic release of the recruited Atg proteins and PI3P turnover, followed by fusion to the yeast vacuole or mammalian lysosome, and consumption therein (Nakatogawa, 2020). It has been proposed that the phagophore grows via the transfer of phospholipids from the endoplasmic reticulum (ER) by the Atg2‐Atg18 complex (Maeda *et al*, 2019; Osawa *et al*, 2019; Valverde *et al*, 2019) and by the conjugation of Atg8 to phosphatidylethanolamine (PE), a process mediated by the E1‐ and E2‐like enzymes Atg7 and Atg3, respectively, upon local activation by E3‐like Atg12‐Atg5‐Atg16 complex (Nakatogawa, 2020). *De novo* biosynthesis of phospholipids may also contribute to phagophore growth (Andrejeva *et al*, 2020; Ogasawara *et al*, 2020; Schutter *et al*, 2020; Orii *et al*, 2021). While studies showed that the autophagosomal membrane is enriched in phosphatidylcholine (PC; Ogasawara *et al*, 2020; Schutter *et al*, 2020; Orii *et al*, 2021), the contribution of this ubiquitous phospholipid to autophagy remains unclear.

Multiple diseases are associated with altered PC metabolism, including steatohepatitis (Li *et al*, 2006), Alzheimer's disease (Mulder *et al*, 2003; Whiley *et al*, 2014), and muscular dystrophy (Mitsuhashi *et al*, 2011), some of which are also linked to defects in autophagy (Dikic & Elazar, 2018). In yeast growing in the absence of exogenous choline, PC is synthesized primarily via the CDP‐DAG pathway, whereby the ER‐resident enzyme Cho2 first methylates PE into phosphatidyl‐monomethylethanolamine (PMME), which is then further methylated into phosphatidyl‐dimethylethanolamine (PDME) and finally into PC by the ER‐resident enzyme Opi3. The secondary CDP‐choline pathway exploits available intra‐ and extracellular choline for synthesis of PC and can compensate for the loss of the CDP‐DAG pathway activity upon supplementation with exogenous choline (Carman & Han, 2011). The yeast mutant Δ*opi3* displays a specific defect in mitophagy (Sakakibara *et al*, 2015), while an impairment in the CDP‐choline pathway results in inhibition of autophagy in mammalian cancer cells (Dupont *et al*, 2014; Andrejeva *et al*, 2020; Ogasawara *et al*, 2020).

Here we use budding yeast to specifically study the role of PC biosynthesis by Opi3 and Cho2 in autophagy. Our study shows for the first time that the phospholipid equilibrium, conferred by PC production via the CDP‐DAG pathway in choline‐deficient conditions, is essential for efficient closure of the phagophore. In CDP‐DAG mutant, however, PS quantitatively substitutes PC in correlation with accumulation of open autophagic membranes. Accordingly, we demonstrate that choline‐dependent *de novo* PC biosynthesis by the CDP‐choline pathway and the concomitant reduction in PS rescue autophagy. Finally, our findings indicate that the choline‐dependent pathway is dispensable when the CDP‐DAG pathway is active. Our work thus highlights the contribution of phospholipid metabolism to autophagosome biogenesis.

## Results

### Loss of PC biosynthesis via the CDP‐DAG pathway specifically impairs autophagy

**Figure 1:**
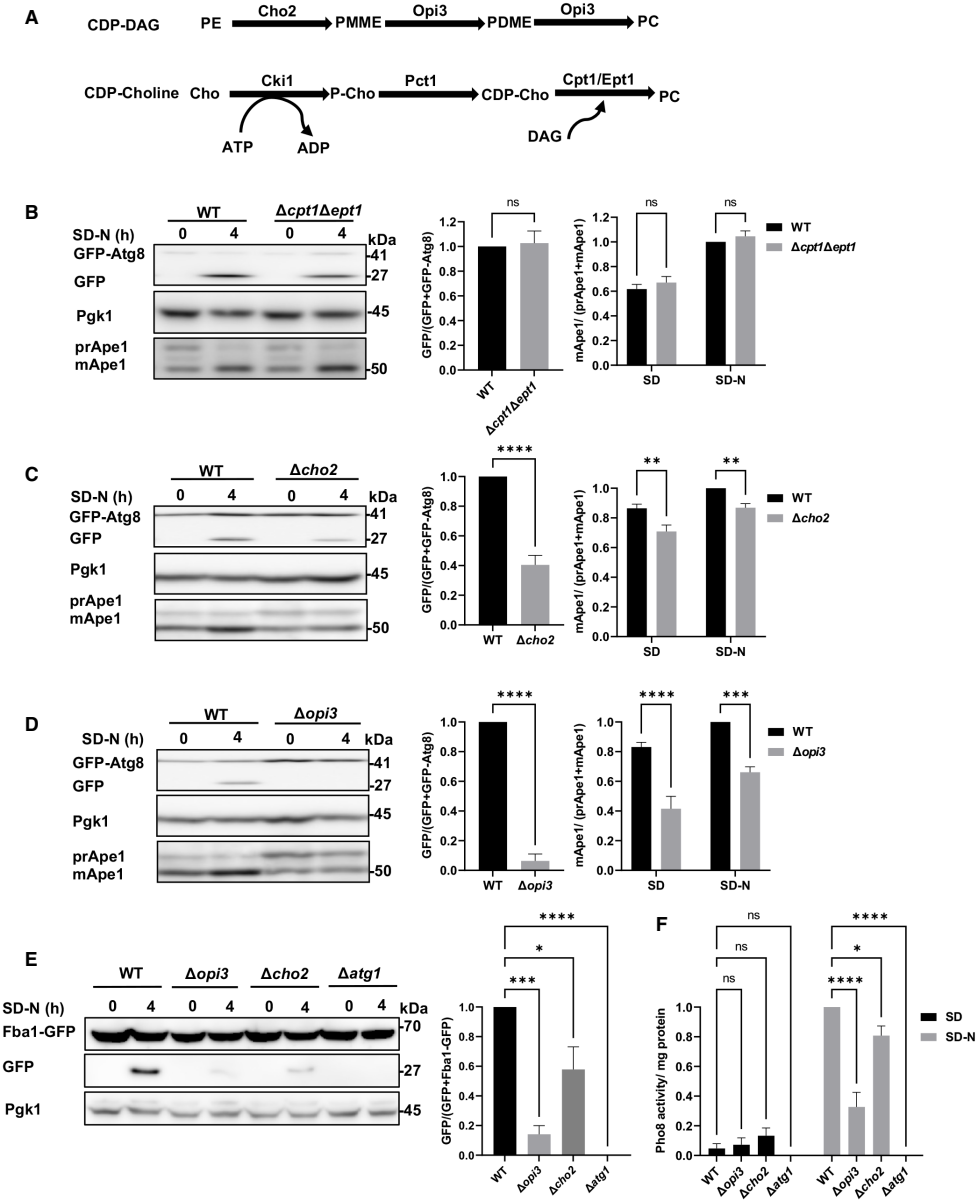
Loss of PC biosynthesis via the CDP‐DAG pathway impairs autophagic flux AScheme of PC biosynthesis pathways in the yeast *Saccharomyces cerevisiae*.B–DWT, ∆*cpt1*∆*ept1* (B), Δ*cho2* (C), or Δ*opi3* (D) cells expressing GFP‐Atg8 were grown to logarithmic phase in SD‐URA, and shifted to SD‐N for 4 h of starvation. Cells were harvested at indicated time points and subjected to western blotting (left panel). Pgk1 was used as a loading control. Middle panel‐ Autophagic activity during starvation was quantified by calculating the ratio of free GFP to total GFP (GFP‐Atg8 + free GFP). Statistical analysis was done by Student's *t*‐test (paired, two tailed; ns, not significant, ****P ≤ 0.0001), error bars represent SEM of at least 3 independent experiments. Right panel‐ Ape1 maturation was quantified by measuring the mApe1 level out of the total Ape1 amount, during SD and SD‐N. Statistical analysis was done by ANOVA multiple comparisons test‐Sidak's, compared with WT (ns, not significant, **P ≤ 0.005, ****P ≤ 0.0001), error bars represent SEM of at least 3 independent experiments.EWT, Δ*opi3*, Δ*cho2* and Δ*atg1* (negative autophagy control) cells expressing chromosomally tagged Fba1‐GFP were grown to log phase in SD, and starved in SD‐N for 4 h. Cells were harvested at indicated starvation time points and subjected to western blotting (left panel). Pgk1 was monitored as a loading control. Right panel—autophagic activity during starvation was quantified, by calculating the ratio of free GFP to total GFP (Fba1‐GFP + free GFP). Statistical analysis was done by ANOVA, Dunnet's multiple comparisons test (*P ≤ 0.05, ***P ≤ 0.0005, ****P ≤ 0.0001), error bars represent SEM of at least 3 independent experiments.FWT, Δ*opi3*, Δ*cho2* and Δ*atg1* cells on the background of *pho8*Δ*60 pho13*Δ strain, were grown to log phase in SD, and starved in SD‐N for 4 h. Pho8 activity was measured by the alkaline phosphatase assay. Statistical analysis was done by ANOVA, Dunnet's multiple comparisons test (ns, not significant, *P ≤ 0.05, ****P ≤ 0.0001), error bars represent SEM of at least 3 independent experiments. Source data are available online for this figure.

To investigate the role of PC in autophagy, we utilized yeast strains lacking genes encoding enzymes involved in PC biosynthesis (Fig 1A). We created either a double knockout of the secondary CDP‐choline pathway enzymes Cpt1 and Ept1 (Δ*cpt1*Δ*ept1*) or the single deletions of the primary CDP‐DAG pathway enzymes Cho2 and Opi3 (Δ*cho2* and Δ*opi3*). Autophagic activity was analyzed by the well‐characterized GFP‐Atg8 cleavage assay (Shintani & Klionsky, 2004), in which upon delivery to the vacuolar lumen, Atg8 is degraded while GFP remains relatively stable, thus allowing assessment of autophagic flux through the quantification of free GFP formation by western blot. As an independent readout for selective autophagic activity, the yeast‐specific biosynthetic cytosol‐to‐vacuole (Cvt) autophagic pathway was also assayed by monitoring Ape1 maturation, as this vacuolar aminopeptidase is synthesized as a precursor (prApe1) in the cytosol, transported to the vacuole via the Cvt pathway, and processed into a mature form (mApe1) in the vacuolar lumen (Lynch‐Day & Klionsky, 2010). As depicted in Fig 1B, when the CDP‐choline pathway double mutant Δ*cpt1*Δ*ept1* was grown to a logarithmic phase and subjected to nitrogen starvation (SD‐N), the Cvt pathway and bulk autophagy were unaltered, as free GFP levels and Ape1 maturation were similar to those of wild‐type (WT) cells. This indicates that the CDP‐DAG pathway is sufficient to sustain autophagosome formation and vacuolar delivery of cargo. In contrast, delivery of Atg8 to the vacuole was impaired and Ape1 maturation was reduced for both CDP‐DAG PC biosynthesis mutants, indicating inhibition of autophagy (Fig 1C and D).

To directly test whether non‐selective autophagy was affected in these strains, we followed the delivery of the cytosolic enzyme fructose 1,6‐bisphosphate aldolase fused to GFP (Fba1‐GFP) to the vacuole using the Fba1‐GFP cleavage assay (Fba1 is a cytosolic protein transported in bulk to the vacuole via non‐selective autophagy). Generation of free GFP indicates transport of Fba1 to the vacuole (Kraft *et al*, 2008). As depicted in Fig 1E, starvation of the WT strain but not autophagy‐deficient Δ*atg1* cells led to a substantial GFP cleavage, which was reduced in Δ*opi3* and Δ*cho2* mutants. As another readout of non‐selective autophagy evaluation we performed the Pho8Δ60 activity assay (Noda & Klionsky, 2008). Indeed, autophagic delivery of cytosolic Pho8Δ60, measured by activation of the enzyme, was reduced in both Δ*opi3* and Δ*cho2* mutants (Fig 1F). Taken together, different independent methods indicate an inhibition of the autophagic flux in PC synthesis mutants.

**Figure 2:**
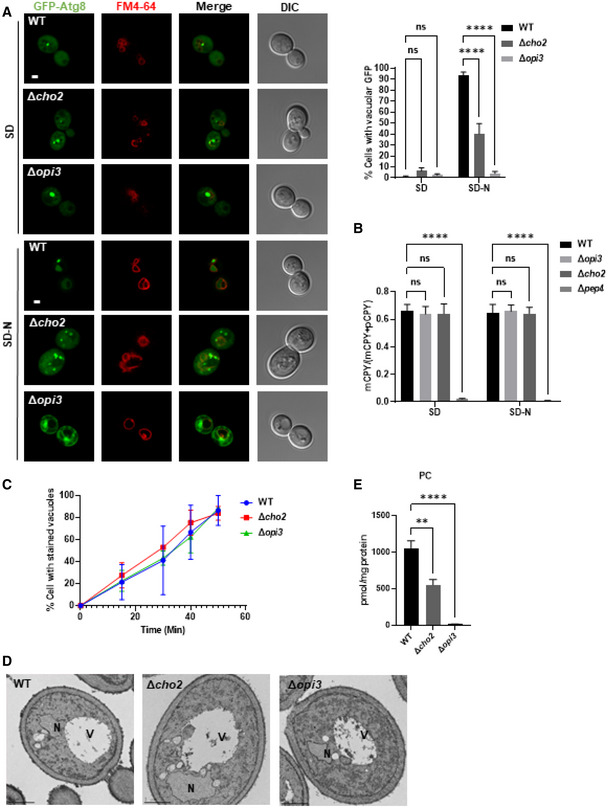
PC deficiency specifically compromises the autophagic pathway Representative images of GFP‐Atg8 (green) and vacuole (red, stained with FM4‐64). WT, Δ*opi3* and Δ*cho2* cells expressing GFP‐Atg8 were grown to log phase in SD‐URA medium and shifted to SD‐N for 3 h. Cells were observed by confocal microscopy before (SD) and during nitrogen starvation (SD‐N; left panel). Scale bar 1 μm. Right panel—quantification of cells with or without GFP inside vacuoles. Statistical analysis was done by ANOVA multiple comparisons test‐Sidak's, compared with WT (****P ≤ 0.0001; ns, not significant), error bars represent SEM of at least 3 independent experiments. Number of cells analyzed for each strain and condition: SD (WT (*n* = 446), Δ*cho2* (*n* = 272), Δ*opi3* (*n* = 272)), SD‐N (WT (*n* = 398), Δ*cho2* (*n* = 224), Δ*opi3* (*n* = 213)).CPY maturation assay: precursor CPY (pCPY) is transported to the vacuole and processed into a mature form (mCPY) in the vacuolar lumen. WT, Δ*opi3* and Δ*cho2* cells, as well as Δ*pep4* cells as negative control for CPY maturation, all expressing GFP‐Atg8, were grown to log phase in SD‐URA, and shifted to SD‐N for 3 h. Cells were harvested at the indicated time points and subjected to western blotting (Fig EV2B). Quantification of CPY maturation (mCPY/(mCPY+pCPY)) is shown for samples in log phase (SD) and starvation (3 h SD‐N). Statistical analysis was done by ANOVA multiple comparison test‐Dunnett's, compared with WT (****P ≤ 0.0001, ns, not significant), error bars represent SEM of at least 3 independent experiments.WT, Δ*opi3* and Δ*cho2* cells were grown to log phase in SD medium, stained for 30 min on ice with FM4‐64, washed 3 times with cold SD, and observed at indicated time points after wash by widefield microscopy (Fig EV2C). Quantification of cells with stained vacuoles (%), statistical analysis was done by Sidak's multiple comparison test compared with WT (ns, not significant), error bars represent SEM of two independent exp. Number of cells analyzed for each strain and time points; (WT: 0 min (*n* = 170), 15 min (*n* = 186), 30 min (*n* = 130), 40 min (*n* = 210), 50 min (*n* = 352)), (Δ*cho2*: 0 min (*n* = 195), 15 min (*n* = 191), 30 min (*n* = 126), 40 min (*n* = 98), 50 min (*n* = 150)), (Δ*opi3*: 0 min (*n* = 148), 15 min (*n* = 200), 30 min (*n* = 224), 40 min (*n* = 221), 50 min (*n* = 216)).WT, Δ*cho2* and Δ*opi3* cells were grown to log phase in SD‐URA medium and shifted to SD‐N for 3 h. Cells were harvested after 3 h of starvation, fixed, embedded and observed by EM. V‐vacuole, N‐nucleus. Scale bar 1 μm.PC levels were quantified by shotgun lipidomics in WT, Δ*opi3* and Δ*cho2* cells expressing GFP‐Atg8 during log phase in SD‐URA. Statistical analysis was done by ANOVA multiple comparisons test‐Dunnett's, compared with WT (**P ≤ 0.005, ****P ≤ 0.0001), error bars represent SEM of 3 independent experiments.

**Figure EV1:**
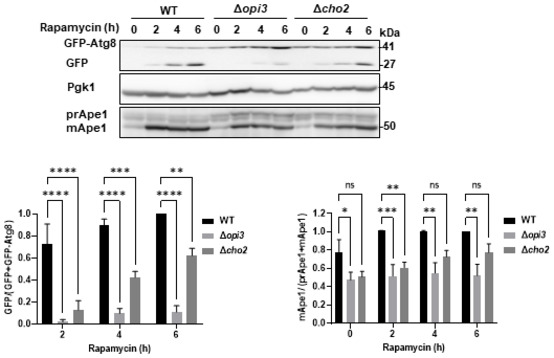
Rapamycin induced autophagy is inhibited in PC deficient strains WT, Δ*opi3* and Δ*cho2* cells expressing GFP‐Atg8 were grown to log phase in SD‐URA and treated with rapamycin (200 ng/ml) for the indicated durations. Cells were harvested at indicated time points and subjected to western blotting (left panel). Pgk1 was monitored as a loading control. Bottom left panel‐ Autophagic activity was quantified during indicated time points of starvation, by calculating the ratio of free GFP to total GFP (GFP‐Atg8 + free GFP). Statistical analysis was done by ANOVA multiple comparison test‐Dunnett's, compared with WT (**P ≤ 0.005, ***P ≤ 0.005, ****P ≤ 0.0001), error bars represent SEM of at least 3 independent experiments. Bottom right panel‐Ape1 maturation was quantified by measuring the mApe1 level out of the total Ape1 amount, during indicated time points. Statistical analysis was done by ANOVA multiple comparison test‐Dunnett's, compared with WT (*P ≤ 0.05, **P ≤ 0.005, ***P ≤ 0.005, ns, not significant), error bars represent SEM of at least 3 independent experiments. Source data are available online for this figure.

**Figure EV2:**
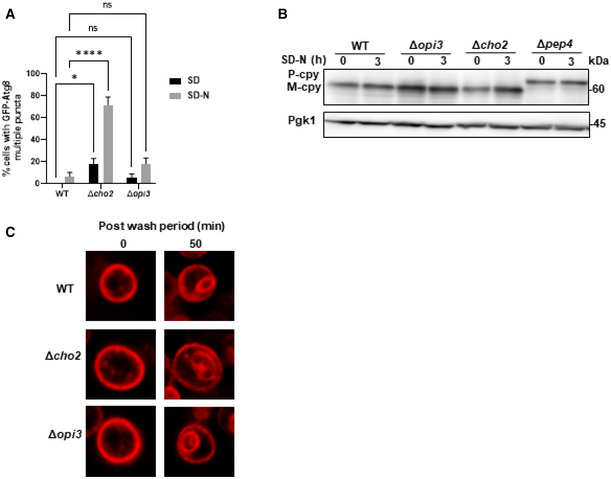
PC deficiency specifically compromises the autophagic pathway Quantification of cells with multiple GFP‐Atg8 puncta during SD and SD‐N. WT, Δ*opi3*, Δ*cho2* cells expressing GFP‐Atg8 were grown to log phase in SD‐URA and shifted to SD‐N for 3 h. Percentage of cells with more than two GFP‐Atg8 puncta per cell GFP‐Atg8 puncta was counted. Statistical analysis was done by ANOVA multiple comparisons test‐Sidak's (*P ≤ 0.05, ****P ≤ 0.0001, ns, not significant), error bars represent SEM of 3 independent experiments. Number of cells analyzed for each strain and condition, SD: WT (*n* = 446), Δ*opi3* (*n* = 272), Δ*cho2* (*n* = 272), SD‐N: WT (*n* = 398), Δ*opi3* (*n* = 213), Δ*cho2* (*n* = 224).CPY maturation assay: precursor CPY (pCPY) is transported to the vacuole and processed into a mature form (mCPY) in the vacuolar lumen. WT, Δ*opi3* and Δ*cho2* cells, as well as Δ*pep4* cells as negative control for CPY maturation, all expressing GFP‐Atg8, were grown to log phase in SD‐URA, and shifted to SD‐N for 3 h. Cells were harvested at the indicated time points and subjected to western blotting (Fig 2B). Pgk1 was monitored as loading control.WT, Δ*opi3* and Δ*cho2* cells were grown to log phase in SD medium, stained for 30 min on ice with FM4‐64, washed 3 times with cold SD, and observed at indicated time points after wash by widefield microscopy (Fig 2C). Scale bar 1 μm. Source data are available online for this figure.

To determine whether autophagy induced by direct inhibition of mTOR was also affected upon CDP‐DAG loss‐of‐function, we treated Δ*cho2* and Δ*opi3* cells with the mTOR inhibitor rapamycin. As depicted in Fig EV1, both mutants exhibited reduced GFP‐Atg8 cleavage and Ape1 maturation, indicating impairment of rapamycin‐induced autophagy. Consistently, confocal microscopy analysis confirmed that during starvation, most Atg8 is translocated into the vacuoles in WT cells but remains mostly cytosolic in Δ*cho2* and Δ*opi3* knockouts (Fig 2A). More particularly, we observed large perivacuolar structures in Δ*opi3* cells and numerous puncta in Δ*cho2* cells (Figs 2A and EV2A).

Importantly, consistent with a previous report (Thibault *et al*, 2012), vacuolar targeting via the endosomal system and maturation of carboxypeptidase Y (CPY, also known as Prc1) remained unaffected upon loss of either Opi3 or Cho2, yet was impaired upon loss of the master vacuolar protease Pep4 (Figs 2B and EV2B). We also show that delivery of the acidic compartment membrane dye FM4‐64 to the vacuole by endocytosis remained unaffected in the PC deficient mutants (Figs 2C and EV2C). Moreover, transmission electron microscopy (TEM) analysis showed no substantial morphological differences of these mutants (Fig 2D). Altogether, these observations indicate that PC biosynthesis by the CDP‐DAG pathway during starvation or inhibition of mTOR is specifically important for autophagy rather than for other intracellular vesicular trafficking processes.

Finally, lipidomic analysis of Δ*opi3* and Δ*cho2* cells revealed a significant drop in PC content in both mutants, namely 48% in the Δ*cho2* mutant and 98.55% in Δ*opi3* cells (Fig 2E). Cumulatively, these results show that autophagy impairment in these two mutants correlates with the cellular PC content, as the autophagy impairment in the Δ*opi3* strain appeared more severe than in the Δ*cho2* knockout and correlated with markedly lower PC level.

### Restoration of phospholipid equilibrium in Δ*opi3* cells rescues autophagy

**Figure 3:**
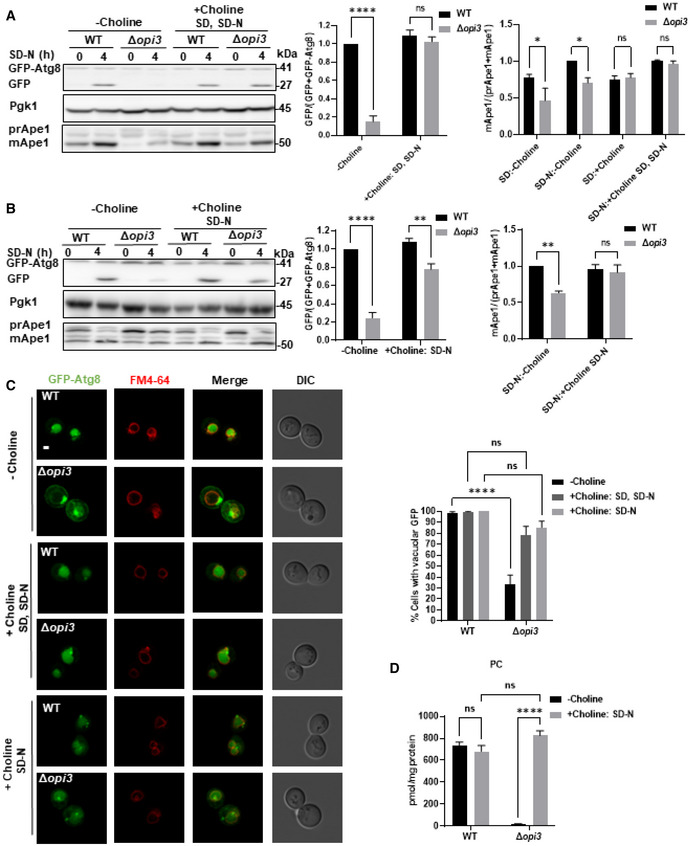
Addition of choline rescues autophagy in Δ*opi3* cells A, BWT and Δ*opi3* cells expressing GFP‐Atg8 were grown to log phase in SD‐URA, and shifted to SD‐N for 4 h, choline (1 mM) was not supplemented (−choline) or supplemented to SD and SD‐N (+choline SD, SD‐N) or only to SD‐N (+choline, SD‐N) as indicated. Cells were harvested at indicated time points and subjected to western blotting (left panel). Pgk1 was monitored as a loading control. Middle panel‐ Autophagic activity was quantified during starvation by calculating the ratio of free GFP to total GFP (GFP‐Atg8 + free GFP). Statistical analysis was done by ANOVA multiple comparisons test‐Sidak's, compared with WT (ns, not significant, **P ≤ 0.005, ****P ≤ 0.0001), error bars represent SEM of at least 3 independent experiments. Right panel‐ Ape1 maturation was quantified by measuring the mApe1 level out of the total Ape1 amount in Δ*opi3* cells at growth (SD) or during starvation (SD‐N), with choline supplemented as indicated. Statistical analysis was done by Sidak's multiple comparisons test compared with WT (ns, not significant, *P ≤ 0.05, **P ≤ 0.005), error bars represent SEM of at least 3 independent experiments.CRepresentative images of GFP‐Atg8 (green) and vacuole (red, stained with FM4‐64). WT and Δ*opi3* cells expressing GFP‐Atg8 were grown in SD‐URA and shifted to SD‐N. Choline (1 mM) was either excluded (−choline), added to growth and starvation medium (+choline SD, SD‐N) or added only to starvation medium (+choline, SD‐N). Cells were observed using confocal microscopy during starvation (left panel). Scale bar 1 μm. Right panel‐ Quantification of cells with GFP inside vacuoles. Statistical analysis was done by ANOVA multiple comparisons test‐Sidak's, compared with WT (****P ≤ 0.0001, ns, not significant), error bars represent SEM of 3 independent experiments. Number of cells analyzed for each strain condition; Choline: WT (*n* = 487), Δ*opi3* (*n* = 316), Choline SD, SD‐N: WT (*n* = 251), Δ*opi3* (*n* = 361), Choline SD‐N: WT (*n* = 320), Δ*opi3* (*n* = 267).DPC levels determined by shotgun lipidomics for WT and Δ*opi3* cells expressing GFP‐Atg8, with or without choline (1 mM) supplementation to SD‐N. Samples were taken after 4 h in SD‐N medium. Statistical analysis was done by ANOVA multiple comparisons test‐Sidak's (****P ≤ 0.0001, ns‐ not significant), error bars represent SEM of at least 3 independent experiments. Source data are available online for this figure.

To examine whether PC biosynthesis by the CDP‐choline route (Fig 1A) could rescue autophagy in CDP‐DAG pathway‐deficient cells, choline was added to the growth and nitrogen starvation media (SD and SD‐N, respectively). As depicted in Fig 3A, impaired activities of autophagy and Cvt pathway in Δ*opi3* cells were fully recovered by addition of choline, implying that restoration of PC biosynthesis rescues autophagy. We next tested whether *de novo* biosynthesis of PC during starvation rescues autophagy by including choline only in the starvation medium (SD‐N). Autophagy was indeed rescued in the Δ*opi3* mutant upon addition of choline to the starvation medium (Fig 3B). Consistently, confocal imaging showed that WT‐like vacuolar delivery of Atg8 was restored while large perivacuolar Atg8 structures were abolished upon addition of choline to growth and starvation media (Choline: SD, SD‐N) or starvation medium alone (Choline: SD‐N; Fig 3C).

**Figure 4:**
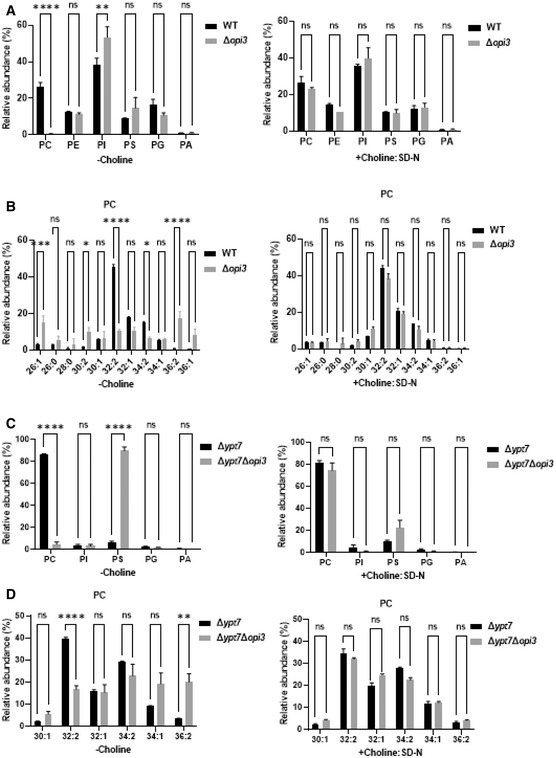
Addition of choline to Δ*opi3* cells specifically restores PC levels Phospholipids species in WT and Δ*opi3* cells. Cells were grown to log phase in SD‐URA, and shifted to starvation (SD‐N) without choline (left panel, −Choline), or supplemented with choline (1 mM) during nitrogen starvation only (Right panel, +Choline: SD‐N). Cells were harvested after 4 h of starvation and analyzed by shotgun lipidomics. Statistical analysis was done by ANOVA multiple comparisons test‐Sidak's (****P ≤ 0.0001, **P ≤ 0.005, *P ≤ 0.05, ns, not significant), error bars represent SEM of at least 3 independent experiments.Acyl chain length and saturation of PC in WT and Δ*opi3* cells. Cells were grown and analyzed as in A.Phospholipid composition of autophagic membranes isolated from Δ*ypt7* and Δ*opi3*Δ*ypt7* cells expressing GFP‐Atg8. Cells were grown to log phase in SD‐URA, and shifted to starvation (SD‐N) without choline (left panel, −Choline), or supplemented with choline (1 mM) during nitrogen starvation only (right panel, +Choline: SD‐N). Cells were harvested after 3 h of starvation and autophagic membranes were isolated and analyzed by shotgun lipidomics as detailed in Materials and Methods. Statistical analysis was done by ANOVA multiple comparisons test‐Sidak's (****P ≤ 0.0001, **P ≤ 0.005, ns, not significant), error bars represent SEM of 3 independent experiments.Acyl chain length and saturation of PC in autophagic membranes isolated from Δ*ypt7* and Δ*opi3*Δ*ypt7* cells expressing GFP‐Atg8. Cells were grown and analyzed as in C.

Lipidomic analysis indicated that addition of choline to the nitrogen starvation medium led to restoration of WT‐like PC levels in Δ*opi3* cells (Fig 3D), showing that the rescue of autophagic flux under starvation conditions correlates with a concomitant *de novo* biosynthesis of PC. Additionally, we evaluated the total phospholipid composition upon *de novo* PC biosynthesis during nitrogen starvation. WT and Δ*opi3* cells had similar amounts of PE, phosphatidylglycerol (PG), PS and phosphatidic acid (PA), yet the Δ*opi3* mutant had higher phosphatidylinositol (PI) and PMME levels, which were only partially reduced upon addition of choline (Fig 4A; Appendix Fig S1F). This attributes the autophagic defects of Δ*opi3* to low PC levels rather than to the presence of PMME or elevated PI levels (Fig 4A; Appendix Fig S1F). No major differences in PC acyl chain composition in the presence of choline were detected in Δ*opi3* in comparison with WT cells in the presence or the absence of choline (Fig 4B; Appendix Fig S1G). Both WT and Δ*opi3* cells had similar fatty acid chain composition of PG and PE, PA, and PS in the presence or absence of choline (Appendix Fig S1A–D), while PI and PMME composition and level remained altered in the presence of choline (Appendix Fig S1E and F).

Furthermore, to evaluate phospholipid content of autophagic membranes in PC deficient and sufficient cells, we enriched Atg8‐positive autophagic membranes by immunoprecipitation of membrane‐bound GFP‐Atg8, as previously done by Graef and coworkers (Schutter *et al*, 2020), and analyzed phospholipid composition as above (Appendix Fig S1H). To establish the method in our hands, we knocked out *YPT7*, a small GTPase required for fusion of autophagosomes with the vacuole, thus allowing accumulation of autophagosomes in the cytosol. Shotgun lipidomic of isolated membranes showed that in PC deficient Δ*opi3*Δ*ypt7* cells, PC levels were low compared with Δ*ypt7* (Fig 4C), while elevated upon choline addition—with no differences in PC acyl‐chain composition (Fig 4C and D). PA, PG, and PI levels and acyl chain compositions were similar in Δ*ypt7* and Δ*opi3*Δ*ypt7* in the presence or absence of choline (Fig 4C; Appendix Fig S1I, K, and L), while PS levels were largely elevated in Δ*opi3*Δ*ypt7*, and restored upon choline addition (Fig 4C), while acyl chain composition was substantially different (Appendix Fig S1J). PE and PMME levels were not detected due to technical problems of this relatively new assay. These results indicate that Atg8‐labeled membranes are enriched for PC, which is drastically lost upon impairment of PC synthesis by the CDP‐DAG pathway, a condition which favors enrichment of PS. Moreover, these findings also indicate that autophagic PC can be rescued by the addition of choline—while maintaining different composition of PS acyl chains.

### Phospholipid imbalance leads to accumulation of *bona fide* autophagic structures

**Figure 5:**
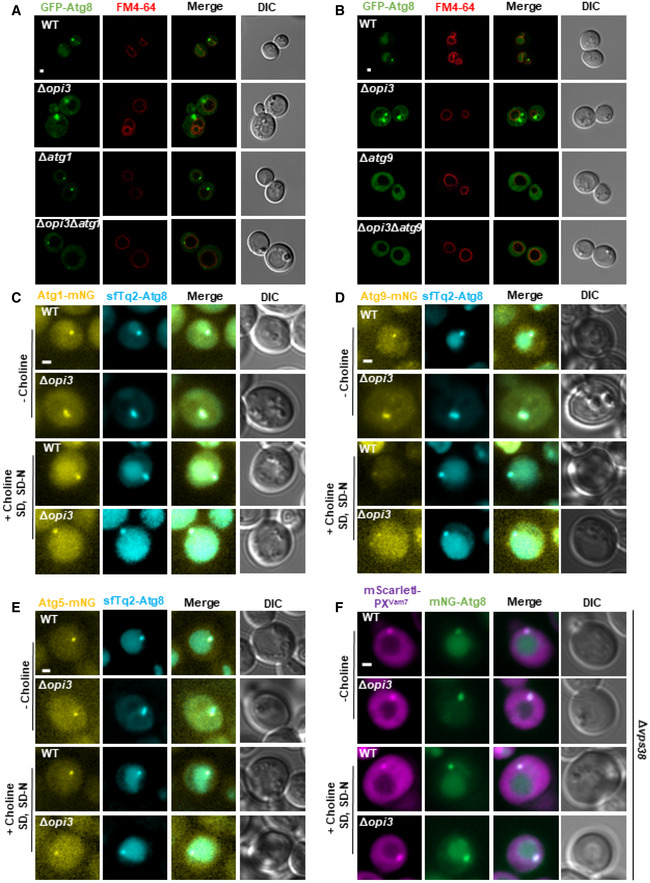
Phospholipid imbalances leads to accumulation of *bona fide* autophagic structures A, BRepresentative images of GFP‐Atg8 (green) and vacuole (red, stained with FM4‐64). WT, Δ*opi3*, Δ*atg1* and Δ*opi3*Δ*atg1* (A) or Δ*atg9* and Δ*opi3*Δ*atg9* (B) cells, all expressing GFP‐Atg8, were grown to log phase in SD‐URA medium, shifted to SD‐N for 3 h and observed by confocal microscopy. Scale bar 1 μm.C–ERepresentative images of mNG‐tagged Atg1 (C), Atg9 (D), or Atg5 (E) colocalized with sfTq2‐Atg8. WT and Δ*opi3* cells were grown to log phase in SD medium, and shifted to SD‐N. Choline (1 mM) was excluded (−choline) or supplemented during growth and starvation (+choline SD, SD‐N) as indicated. Images were taken during SD‐N (1–3 h) by widefield microscopy. Scale bar 1 μm.FRepresentative images of mNG‐tagged Atg8 colocalized with mScarletI‐PX^Vam7^. WT and Δ*opi3* cells, expressing mScarletI‐PX^Vam7^ under the CUP1 promoter from the knocked‐out *VPS38* locus, were grown to log phase in SD medium in the presence of 10 μM copper sulfate, and shifted to SD‐N in the presence of 10 μM copper sulfate. Choline (1 mM) was excluded (−choline) or supplemented during growth and starvation (+choline SD, SD‐N) as indicated. Images were taken during SD‐N (1–3 h) by widefield microscopy. Scale bar 1 μm.

**Figure EV3:**
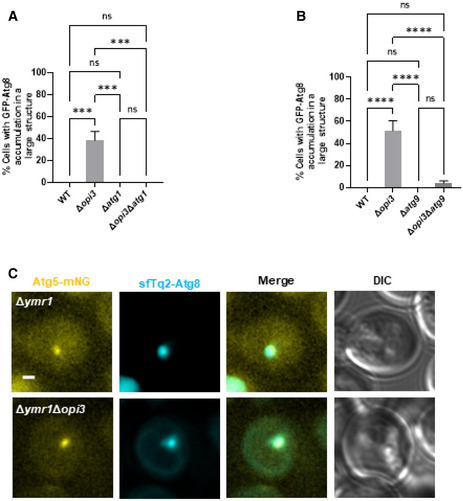
Phospholipid imbalance leads to accumulation of *bona fide* autophagic structures A, BQuantification of imaged data shown in Fig 5A and B, respectively, of cells with GFP‐Atg8 accumulation in large structures. Statistical analysis was done by ANOVA multiple comparisons test‐Tukey's (****P ≤ 0.0001, ns, not significant) error bars represent SEM of least 3 independent experiments. Number of cells analyzed for each strain. Panel (A): WT (*n* = 298), Δ*opi3* (*n* = 510), Δ*atg1* (*n* = 179), Δ*opi3*Δ*atg1* (*n* = 177), Panel (B): WT (*n* = 349), Δ*opi3* (*n* = 564), Δ*atg9* (*n* = 275), Δ*opi3*Δ*atg9* (*n* = 253).CRepresentative images of mNG‐tagged Atg5 colocalized with sfTq2‐Atg8 on the background of Δ*ymr1*. WT and Δ*opi3* cells were grown to log phase in SD medium, and shifted to SD‐N. Images were taken during SD‐N (1–3 h) by widefield microscopy. Scale bar 1 μm.

As indicated above (Figs 2A and 3C), starved Δ*opi3* cells accumulate large perivacuolar Atg8‐positive structures, while most Atg8 is translocated to the vacuole in WT cells. To determine whether accumulated structures in PC deficient cells are aberrant autophagic membranes, we further knocked out core the autophagy gene *ATG1* or *ATG9*, and analyzed the effect of these deletions on formation of these structures. As depicted in Figs 5A and B, and EV3A and B, knockout of *ATG1* or *ATG9* in Δ*opi3* cells abolished the accumulation of the large Atg8‐positive structures, showing a similar distribution of GFP‐Atg8 as the cognate deletions in the WT background. This suggests that starvation induces accumulation of aberrant autophagic structures in Δ*opi3*.

To establish the *bona fide* autophagic identity of these structures, we co‐expressed Atg1 or Atg9 tagged at their C‐terminus with mNeonGreen (mNG), in WT or Δ*opi3* cells with superfolder mTurquoise2‐tagged Atg8 (sfTq2‐Atg8). As depicted in Fig 5C and D, Atg1 and Atg9 mostly colocalized with large Atg8‐positive structures, corroborating their autophagic identity. Importantly, these aberrant structures were lost in the presence of choline (Fig 5C and D), suggesting that PC biosynthesis by the CDP‐choline pathway is sufficient to prevent or resolve their accumulation. As Atg1 and Atg9 can be present on both phagophores and autophagosomes, we also examined the localization of phagophore marker protein Atg5 (Graef *et al*, 2013; Suzuki *et al*, 2013; Nishimura & Tooze, 2020). As shown in Fig 5E, Atg5 colocalizes with the Atg8 elongated‐positive structures in Δ*opi3* cells, as previously visualized for non‐selective autophagy (Graef *et al*, 2013), and these elongated Atg8 structures were absent in the presence of choline. For better visualization of Atg5 on immature autophagic structures, we depleted Ymr1, the major phosphatase required for autophagosome maturation (Cebollero *et al*, 2012; Cheng *et al*, 2014), which allows comparison between Δ*ymr1* and Δ*opi3*Δ*ymr1* immature structures. As depicted in Fig EV3C, Atg5 clearly colocalized with Atg8‐positive structures in both strains.

We next corroborated phagophore identity by independent readout of PI3P, a well‐known marker of autophagic membranes. To this end, we visualized PI3P by fusing the fluorescent protein mScarletI to the PX domain of the v‐SNARE Vam7, which binds PI3P (Cheever *et al*, 2001). We integrated this mScarletI‐PX^Vam7^ expression cassette into the *VPS38* locus—to focus on autophagic (Atg14‐dependent) but not endosomal (Vps38‐dependent) PI3P (Kihara *et al*, 2001). As depicted in Fig 5F, PI3P colocolized along Atg8‐positive membranes in both WT and Δ*opi3*, and choline rescued the vacuolar translocation of cytosolic Atg8 in Δ*opi3* cells (as above) without dramatically changing the distribution of PI3P. These data strength the notion that Δ*opi3* cells accumulate *bona fide* autophagic membranes.

### Phospholipid equilibrium promotes completion of autophagosome formation

**Figure 6:**
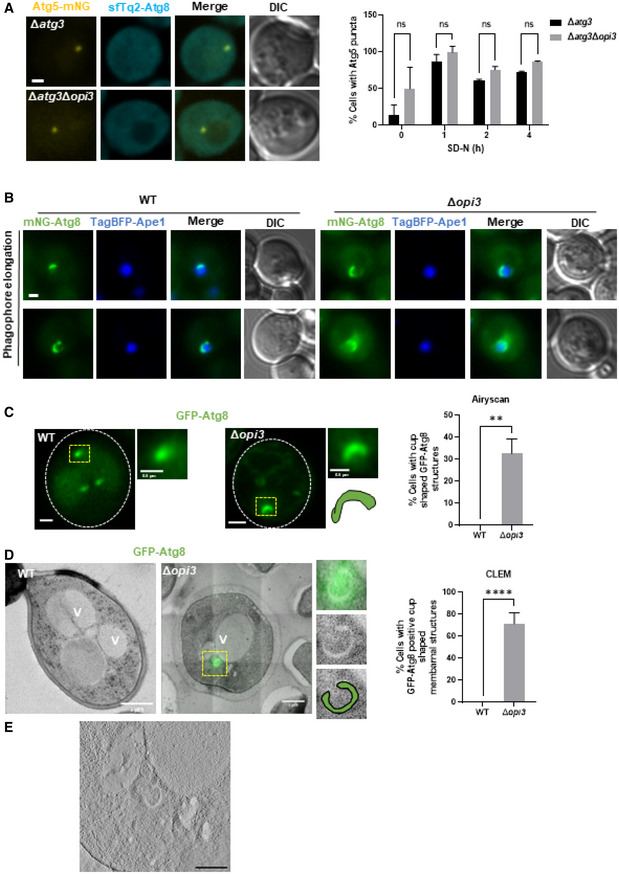
Phospholipid imbalance leads to accumulation of elongated unsealed phagophores Representative Images of Atg5‐mNG with sfTq2‐Atg8 during SD‐N. Δ*atg3* and Δ*atg3*Δ*opi3* cells were grown to log phase in SD medium, shifted to SD‐N for 4 h, and observed by Widefield microscopy (left panel), scale bar 1 μm. Right panel‐ quantification of percentage of cells with Atg5 puncta during different time points. Statistical analysis was done by ANOVA multiple comparisons test‐Sidak's (ns, not significant), error bars represent SEM of 2 independent experiments. Number of cells analyzed for each strain and time point (0 h: Δ*atg3* (*n* = 296), Δ*atg3*Δ*opi3* (*n* = 111), 1 h: Δ*atg3* (*n* = 325), Δ*atg3*Δ*opi3* (*n* = 110), 2 h: Δ*atg3* (*n* = 338), Δ*atg3*Δ*opi3* (*n* = 135), 4 h: Δ*atg3* (*n* = 309), Δ*atg3*Δ*opi3* (*n* = 167)).Representative images of WT and Δ*opi3* cells expressing TagBFP‐Ape1 under the native APE1 promoter and Ape1 under the CUP1 promoter. Cells were grown to log phase in SD‐LEU medium in the presence of 500 μM copper sulfate, and observed by Widefield microscopy, scale bar 1 μm.Representative images of WT and Δ*opi3* cells expressing GFP‐Atg8. Cells were grown to log phase in SD‐URA medium, shifted to SD‐N for 3 h and observed by Airyscan microscopy (left panel). White dashes indicate cell boundaries, yellow dashes indicate autophagic structures. For each cell—Magnification of yellow dashed area (top right), schematic representation (bottom right). Scale bar 1 μm. Right panel‐ quantification of the percentage of cells with cup shaped GFP‐Atg8 structures imaged by Airyscan microscopy. Statistical analysis was done by Student's *t*‐test (unpaired; **P ≤ 0.05), error bars represent SEM of least 3 independent experiments. Number of cells analyzed for each strain, WT (*n* = 45), Δ*opi3* (*n* = 110).Representative CLEM images of WT and Δ*opi3* cells expressing GFP‐Atg8, grown to log phase in SD‐URA medium and shifted to SD‐N for 3 h. Cells were harvested, deep frozen and processed for CLEM (left panel), V‐vacuole. For Δ*opi3* cell—Magnification of yellow dashed area of GFP‐Atg8 positive phagophore (top right), TEM image only (middle right), schematic representation of a phagophore (bottom right). Scale bar 1 μm. Right panel‐ quantification of the percentage of cells with GFP‐Atg8 positive cup shaped membrane structures imaged by CLEM. Statistical analysis was done by Student's *t*‐test (unpaired; ***P ≤ 0.005), error bars represent SEM of least 3 independent experiments. Number of cells analyzed for each strain, WT (*n* = 20), Δ*opi3* (*n* = 21).Tomogram slice of CLEM Δ*opi3* image (D), focused on the phagophore, thickness of 120 nm, scale bar 500 nm.

To better characterize the step of autophagosome biogenesis that is affected by insufficient PC, we first sought to arrest the process before membrane elongation but after PAS and phagophore formation. To this end, we knocked out in WT or Δ*opi3* cells the E2‐like enzyme *ATG3*, which conjugates Atg8 to membrane‐resident PE to promote elongation (Xie *et al*, 2008), and determined Atg5 recruitment to the PAS upon nitrogen starvation. As shown in Fig 6A, recruitment of Atg5‐mNG to the PAS was not impaired by the absence of Opi3. To further determine whether phagophore elongation was affected in the Δ*opi3* mutant, we overexpressed the selective autophagy cargo Ape1 tagged with TagBFP (Suzuki *et al*, 2013; Pfaffenwimmer *et al*, 2014). As showed in Fig 6B, elongated mNG‐Atg8‐positive phagophores encircled a giant TagBFP‐Ape1 complex in both WT and Δ*opi3* mutant to a similar extent, indicating that the ability of the phagophore membrane to elongate was not affected by the lack of PC.

**Figure EV4:**
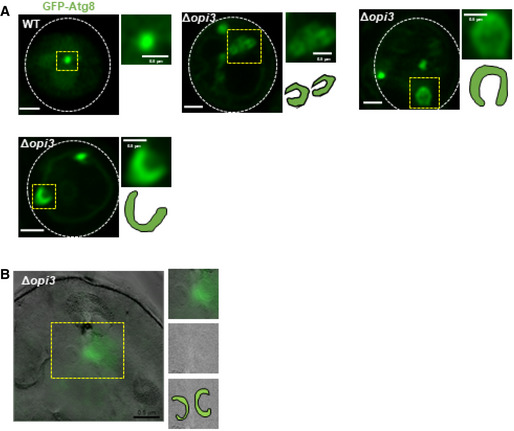
Phospholipid imbalance leads to accumulation of elongated unsealed phagophores Representative images of autophagic structures in WT and Δ*opi3* cells expressing GFP‐Atg8. Cells were grown to log phase in SD‐URA medium, shifted to SD‐N for 3 h and observed by Airyscan microscopy. White dashed lines indicate cell boundaries, yellow dashed area indicate autophagic structures. For each cell—whole cell (left), magnification of yellow dashed area (top right) and schematic representation (bottom right). Scale bar 1 μm, 0.5 μm (enlarged images).Representative CLEM image of Δ*opi3* cells expressing GFP‐Atg8, grown to log phase in SD‐URA medium and shifted to SD‐N for 3 h. Cells were harvested, deep frozen and processed for CLEM (left), V‐vacuole. For each cell—magnification of yellow dashed area of GFP‐Atg8 positive phagophore (top right), TEM image only (middle right), schematic representation of phagophores (bottom right). Scale bar 1 μm (left).

The observations above suggest that autophagy in Δ*opi3* is stalled at a step following elongation of the phagophore. Indeed, fast Airyscan super‐resolution microscopy detected GFP‐Atg8‐positive cup‐shape structures in Δ*opi3* (but not WT) cells, with variable rim apertures (Figs 6C and EV4A).

To gain higher resolution and ultrastructural insight into Δ*opi3* phagophores, we turned to correlative light electron microscopy (CLEM). To this end, starved WT and Δ*opi3* cells expressing GFP‐Atg8 were deep‐frozen, fixed, and analyzed by both widefield fluorescence microscopy and TEM (see materials and methods). CLEM analysis indicated that most of the fluorescent GFP‐Atg8 signal in Δ*opi3* cells correlated with ultrastructural observations of cytosolic unsealed double‐membrane structures engulfing cytosolic material—namely phagophores (Figs 6D and EV4B). In WT cells neither GFP‐Atg8 fluorescence nor cytosolic double‐membrane structures could be located (Fig 6D), probably due to rapid maturation and vacuolar consumption of autophagic structures in unperturbed conditions. Electron tomography further indicated an open elongated shape of phagophore in the PC deficient mutant (Fig 6E; Movie EV1).

**Figure 7:**
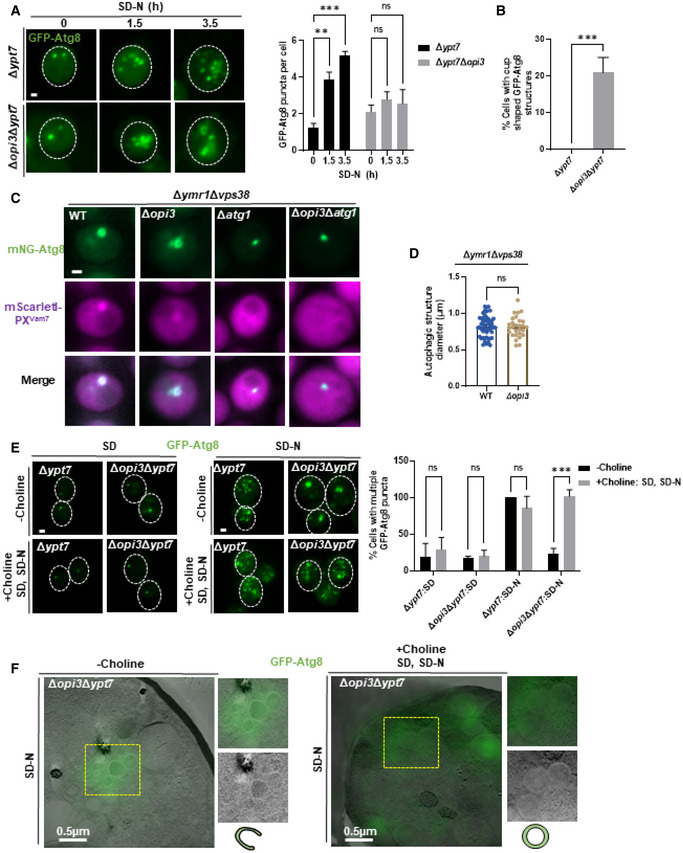
Phospholipid balance promotes succession of autophagosome biogenesis Representative images of Δ*ypt7*, Δ*opi3*Δ*ypt7* expressing GFP‐Atg8 (z‐stack projection). Cells were grown to log phase in SD‐URA medium and starved in SD‐N, images were taken during starvation in indicated time points by widefield microscopy (left panel). Scale bar 1 μm. Right panel‐ quantification of GFP‐Atg8 puncta per cell in Δ*ypt7*, Δ*opi3*Δ*ypt7* at indicated time points. Statistical analysis was done by ANOVA multiple comparisons test‐Sidak's (***P ≤ 0.001, **P ≤ 0.005, ns, not significant), compared with WT. Error bars represent SEM of least 3 independent experiments. Number of cells analyzed for each strain and time point, 0 h: Δ*ypt7* (*n* = 322), Δ*opi3*Δ*ypt7* (*n* = 194), 1.5 h: Δ*ypt7* (*n* = 220), Δ*opi3*Δ*ypt7* (*n* = 155), 3.5 h Δ*ypt7* (*n* = 216), Δ*opi3*Δ*ypt7* (*n* = 307).Quantification of cup‐shaped phagophores in Δ*ypt7* and Δ*opi3*Δ*ypt7* cells expressing GFP‐Atg8 during starvation (1‐3 h). Statistical analysis was done by Student's *t*‐test (***P ≤ 0.001), error bars represent SEM from at least 3 independent experiments. Number of cells analyzed for each strain, Δ*ypt7* (*n* = 347), Δ*opi3*Δ*ypt7* (*n* = 474).Representative images of mNG‐tagged Atg8 colocalized with mScarletI‐PX^Vam7^. WT, Δ*opi3*, Δ*atg1* and Δ*opi3*Δ*atg1* cells on the background of Δ*ymr1*, expressing mScarletI‐PX^Vam7^ under the CUP1 promoter from the knocked‐out *VPS38* locus, were grown to log phase in SD medium in the presence of 10 μM copper sulfate, and shifted to SD‐N in the presence of 10 μM copper sulfate. Images were taken during SD‐N (1–3 h) by widefield microscopy. Scale bar 1 μm.Quantification of autophagic structure diameters in WT and Δ*opi3* cells on the background of Δ*ymr1*Δ*vps38* (C) by ImageJ. Statistical analysis was done by Student's *t*‐test, unpaired, two sided (ns, not significant), error bars represent SEM of at least three independent experiments. Number of cells analyzed for each strain, WT (*n* = 51), Δ*opi3* (*n* = 28).Representative images of Δ*ypt7*, Δ*opi3*Δ*ypt7* cells expressing GFP‐Atg8 (projection). Cells were grown to log phase in SD‐URA medium and starved in SD‐N, with supplementation of choline (1 mM) to SD and SD‐N as indicated. Images were taken during growth and starvation by confocal microscopy (left panel). Scale bar 1 μm. Right panel‐ quantification of cell with more than two GFP‐Atg8 puncta, in Δ*ypt7* and Δ*opi3*Δ*ypt7* at growth (SD) or starvation (SD‐N) in the presence (+choline: SD, SD‐N) or absence (−choline) of choline (1 mM). Incidence were normalized to Δ*ypt7* (SD‐N) control. Statistical analysis was done by ANOVA multiple comparisons test‐Sidak's (ns, not significant, ***P ≤ 0.001). Error bars represent SEM of least 3 independent experiments. Number of cells analyzed for each strain and condition, SD: Δ*ypt7* (*n* = 209), Δ*opi3*Δ*ypt7* (*n* = 111), SD‐N: Δ*ypt7* (*n* = 118), Δ*opi3*Δ*ypt7* (*n* = 103), SD + Choline: Δ*ypt7* (*n* = 107), Δ*opi3*Δ*ypt7* (*n* = 120), SD‐N+ Choline: Δ*ypt7* (*n* = 84), Δ*opi3*Δ*ypt7* (*n* = 74).CLEM images of Δ*opi3*Δ*ypt7* cells expressing GFP‐Atg8, grown to log phase in SD‐URA medium and shifted to SD‐N for 4 h, with supplementation of choline (1 mM) excluded (−choline) or present at growth and starvation (+choline SD, SD‐N) as indicated. Cells were harvested, deep frozen and processed for CLEM. For each cell—whole cell (left), magnification of yellow dashed area of GFP‐Atg8 positive phagophore (top right), TEM image only (middle right), schematic representation of autophagic structure (bottom right). Scale bar 0.5 μm.

**Figure EV5:**
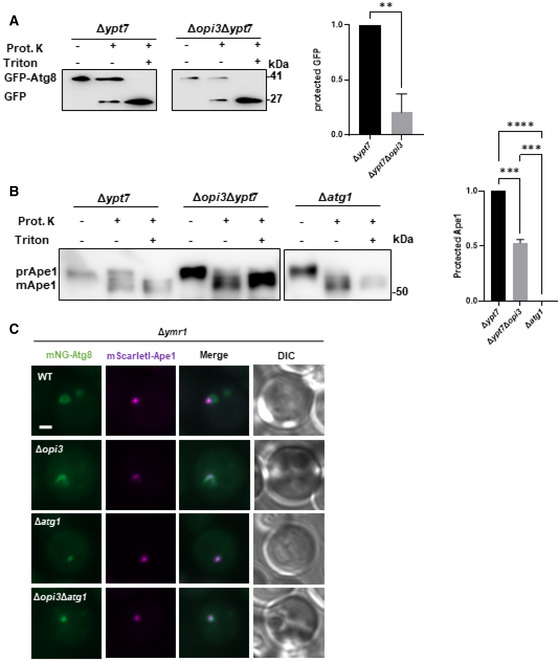
Phospholipid balance promotes succession of autophagosome completion A, BΔ*ypt7* and Δ*opi3*Δ*ypt7* mutant cells expressing GFP‐Atg8 were grown to log phase in SD‐URA medium and shifted to SD‐N for 3 h, cell lysates were subjected to protease protection assay combined with immunoblot analysis (left panel). GFP‐Atg8 processing (A) or Ape1 Processing (B) were determined with or without addition of proteinase K (Prot. K), in the presence or absence of detergent (Triton). Protection of GFP‐Atg8/ ape1 may be assessed when proteinase K is added without detergent. Right panel (A)‐ protected GFP‐the ratio of GFP‐Atg8 to total GFP (GFP‐Atg8 + free GFP). Statistical analysis was done by Student's *t*‐test (paired, two tailed; **P ≤ 0.005), error bars represent SEM of at least 3 independent experiments. Right panel (B)‐ protected Ape1‐the ratio of prApe1 to total Ape1 (PrApe1 + mApe1). Statistical analysis was done by ANOVA multiple comparison Tukey's test (****P ≤ 0.0001, ***P ≤ 0.001).CRepresentative images of mNG‐tagged Atg8 colocalized with mScarletI‐Ape1 expressed under the CUP1 promoter on the background of Δ*ymr1*. WT, Δ*opi3*, Δ*atg1* and Δ*opi3*Δ*atg1* cells were grown to log phase in SD medium in the presence of 10 μM copper sulfate, and shifted to SD‐N in the presence of 10 μM copper sulfate. Images were taken during SD‐N (1–3 h) by widefield microscopy. Scale bar 1 μm. Source data are available online for this figure.

To test whether the aberrant stalling of phagophores correlates with low incidence of autophagosome completion in PC deficient cells, we performed a protease protection assay (Nair *et al*, 2011). Indeed, cargo protection in Δ*ypt7*Δ*opi3* cells was lower than in Δ*ypt7* cells (Fig EV5A and B), implicating PC deficiency in impairment of the capacity or rate of autophagosome completion. To directly demonstrate a kinetic delay in formation of closed autophagosomes due to loss of PC synthesis, we counted the number of GFP‐Atg8 positive structures in Δ*ypt7*Δ*opi3* and Δ*ypt7* cells over time upon starvation. As depicted in Fig 7A, Δ*ypt7* accumulated GFP‐Atg8 puncta over time, while Δ*ypt7*Δ*opi3* exhibited a constant average count of autophagic structures, in correlation with detection of a visibly open elongated structures, which were detected only in the PC‐deficient cells (Fig 7B). We therefore conclude that a maturation defect in PC deficient cells precludes further completion and formation rounds of numerous autophagosomes—resulting in loss of autophagic flux (as shown above).

To unbiasedly compare the size of the autophagic structures between PC deficient and PC sufficient cells we utilized mutant cells lacking Ymr1, as described above—thus allowing clear visual comparison of a single terminal structure between Δ*ymr1* and Δ*opi3*Δ*ymr1* cells, on the background of the strains with PI3P marker (in the absence of Vps38 as in Fig 5F). As depicted in Fig 7C, PI3P marker was enriched in the mNG‐Atg8‐positive structure in Δ*ymr1* and Δ*opi3*Δ*ymr1* cells. Importantly, those structures were not formed in the Δ*ymr1*Δ*atg1* and Δ*ymr1*Δ*opi3*Δ*atg1* controls. These structures were also colocalized with Ape1, a phagophore and PAS marker (Fig EV5C). As depicted in Fig 7D, the immature autophagic structures detected in Δ*ymr1* and Δ*opi3*Δy*mr1* strains showed similar diameters (Fig 7D), indicating that in the absence of Opi3 phagophore expansion remains intact, while the loss of autophagosome accumulation and cargo protection is specifically due to impaired closure.

We then asked whether this arrest in biogenesis due to the reduction of PC levels can be alleviated by supplementation of choline and restoration of PC levels. Strikingly, addition of choline, shown above to suppress the loss of flux in Δ*opi3* cells (Fig 3), also restored the appearance of numerous GFP‐Atg8 puncta in Δ*ypt7*Δ*opi3* cells as observed in Δ*ypt7* regardless of choline (Fig 7E), indicating a direct relation between PC levels, progression and completion of autophagosome biogenesis. To further challenge our interpretation, we followed the mobility of Atg8‐positive structures in Δ*opi3* Δ*ypt7* cells (Movies [Link], [Link]). Motion of GFP‐Atg8 puncta in PC‐sufficient Δ*ypt7* cells was evidently fast (Movie EV2), whereas in PC‐deficient Δ*opi3*Δ*ypt7* cells Atg8‐positive membranes remain mostly immobile (Movie EV3). Importantly, mobility in Δ*opi3*Δ*ypt7* was recovered upon addition of choline (Movie EV5), without a further effect on Δ*ypt7* structures (Movie EV4). This finding is line with the notion that phagophores are immobile while mature autophagosome are mobile (Fass *et al*, 2006).

To better visualize stalled phagophore structures in the presence or depletion of PC, we performed CLEM analysis of Δ*ypt7*Δ*opi3* cells with or without choline. In the absence of choline, structures with open rims are readily observed, together with circular structures where the rim cannot be detected—some of which are possibly closed. Upon addition of choline, however, open rims are not found (Fig 7F), further supporting the notion that PC deficiency impairs closure, which in turn precludes formation of complete autophagosomes. We therefore conclude that PC promotes the transition from phagophore elongation to sealing and subsequent autophagosome maturation, which in turn allows efficient succession of autophagosome biogenesis.

## Discussion

Understanding the process of autophagosome biogenesis in molecular terms has been the focus of intensive research over the past two decades (Nakatogawa, 2020). The earlier stages of the autophagosome biogenesis, i.e., phagophore nucleation and expansion, have been relatively characterized. In contrast, less is known on later stages, i.e. phagophore sealing and autophagosome maturation, envisioned as occurring spontaneously or facilitated by hitherto‐unknown molecules, namely proteins and lipids. It has been recently reported that autophagic membranes are enriched with PC (Andrejeva *et al*, 2020; Ogasawara *et al*, 2020; Schutter *et al*, 2020; Orii *et al*, 2021; Schmitt *et al*, 2022), but the exact contribution of this phospholipid to the process has remained obscure. In this study, we show that sufficient PC biosynthesis by the CDP‐DAG pathway enzyme Opi3 promotes transition from phagophore elongation to autophagosome maturation, while during starvation the CDP‐choline pathway can take over this role.

Δ*opi3* cells accumulate stalled Atg8‐positive structures that we identified as immature phagophores based on their genetic dependency on core *ATG* genes. Standard confocal and widefield as well as Airyscan high‐resolution fluorescence microscopy, complemented by CLEM, supplemented with colocalization of autophagic membrane markers such as Atg1, Atg9, Ape1, and PI3P, and particularly the phagophore‐exclusive Atg5—which decorate these structures in Δ*opi3* cells, also served to support this notion. The stalling of core Atg proteins on these intermediate structures in the absence of active recycling back to the cytoplasm may explain the observed inhibition in the completion of new rounds of autophagosome biogenesis (Cebollero *et al*, 2012; Nair *et al*, 2012).

Importantly, our data indicate that yeast deficient in PC biosynthesis are nevertheless competent for formation of pre‐autophagosomal structures and phagophore elongation, while mature autophagosomes still form, albeit with reduced efficiency. The defect in the progression of phagophores into mature autophagosomes may be attributed to perturbed physical membrane properties upon lipid composition disequilibrium. The cylindrical phospholipid PC favors lipid bilayer integrity (Boumann *et al*, 2006) and contributes to membrane fluidity (Bao *et al*, 2021). These roles may be satisfied in the autophagic membranes of Δ*opi3* cells by the quantitative substitution for PS, an equally cylindrical phospholipid (Osman *et al*, 2011; McMahon & Boucrot, 2015). It is possible that the loss of PC or accumulation of PS negatively affects proper recruitment of closure‐promoting factors (Zhen *et al*, 2021). Furthermore, in agreement with other studies (Reunanen *et al*, 1985; Schutter *et al*, 2020), Atg8 membranes of both WT and PC deficient cells have high amount of unsaturated fatty acids chains, which may favor membrane fluidity and phagophore elongation together with preservation of optimal environment for lipid transfer from the ER (van der Veen *et al*, 2017). This may account for the retention of WT‐like phagophore size upon depletion of PC as observed in our study. A recent report by Baumeister and coworkers (Bieber *et al*, 2022), has indicated that the phagophore rim has a complex architecture that may be particularly prone to phospholipid imbalance, thereby compromising membrane closure while maintaining expansion of the relatively flat majority of the phagophore surface.

In our hands, Opi3 deficiency maintains moderate prominence of PS throughout the cell while largely substituting PC for PS in the phagophore. This may be attributed to the recently reported preference of the Atg2 protein family (as assayed in mammalian ATG2A/B) to negatively charged phospholipids in general and particularly PS (Maeda *et al*, 2019; Osawa *et al*, 2020; Tan & Finkel, 2022). Along this line, down‐regulation of fruit fly Atg2 leads to abnormal enrichment in accumulated open phagophores of negatively charged phospholipids, most pronouncedly PS (Laczko‐Dobos *et al*, 2021). Moreover, a domain enriched with PS and PC synthesis enzymes was observed in close proximity to the mammalian omegasome (Nishimura *et al*, 2017). Future studies will further elucidate the relevance of specific phospholipid synthesis to the integrity of the evolving phagophore membrane.

The severity of impairment in autophagic flux was greater in Δ*opi3* cells than in the Δ*cho2* mutant, in correlation with their respective degree of deficiency in cellular PC levels, suggesting a quantitative contribution of PC to autophagy. This is in line with recent reports in mammalian cells showing that abrogation of PC biosynthesis leads to inhibition of autophagy (Dupont *et al*, 2014; Andrejeva *et al*, 2020; Ogasawara *et al*, 2020). Intriguingly, loss of Opi3 was originally linked to impaired mitophagy but not starvation‐induced non‐selective autophagy in yeast (Sakakibara *et al*, 2015), which may be explained by the fact that cells under starvation in that study were initially grown in the presence of choline (within the YPD growth medium). The authors suggested that conjugation of Atg8 to accumulated PMME in the absence of Opi3 may lead to inhibition of deconjugation by Atg4 (Sakakibara *et al*, 2015). However, in our hands, the restoration of PC levels by a timely addition of choline was not accompanied by massive loss of pre‐accumulated PMME levels in Δ*opi3* cells, suggesting that autophagic impairment is mainly attributed to the lipid composition imbalance conferred by low PC levels, rather than the dominant‐negative effect of PMME.

Furthermore, the combined prominence of PMME and PS upon PC deficiency could positively compensate for the membrane stress resulting from the loss of PC, which could favor phagophore formation and elongation but not closure. Indeed, supplementation with choline rescues the progression of autophagosome biogenesis in Δ*opi3* knockout cells and restores autophagic flux, emphasizing the contribution of PC itself to autophagy rather than a preference for CDP‐DAG pathway over the CDP‐choline pathway in lipid supply to autophagy. This is in line with the lipidomic observation of restored PC and PS levels under these conditions, with relatively minor alterations in the levels of other phospholipids in Atg8‐enriched membranes. The regained efficiency in autophagosome completion upon restoration of PC synthesis may be also attributed to the similar acyl chain composition of PC derived from both biosynthetic pathways during starvation. The ability to rescue autophagy by starvation‐specific *de novo* synthesis of PC is in line with the recently reported broader importance of lipid synthesis in autophagosome biogenesis (Nishimura *et al*, 2017; Schutter *et al*, 2020). However, it is still unclear whether autophagic membranes acquire PC by resident synthesis, directly from the ER or via membrane contact site with other organelles or vesicular transport, or combinations of the above.

To conclude, our study reveals a role of PC synthesis and the ensuing phospholipid balance in autophagy—particularly in the step of autophagosome completion. The detrimental effect of PC/PS substitution highlights the importance of accurate spatio‐temporal phospholipid synthesis and sorting for the integrity of the autophagic membrane. Our study thus sheds new light on the interplay between phospholipid metabolism and autophagy.

## Materials and Methods

### Yeast strains and media

Yeast strains used in this study are listed in Table 1. Transformation was performed by the lithium acetate method (Gietz & Woods, 2002). Cells were grown in synthetic minimal medium (SD; 0.67% yeast nitrogen base without amino acids, 2% glucose supplemented with amino acids), or starved for nitrogen (SD‐N; 2% glucose, 0.17% yeast nitrogen base without amino acids and ammonium sulfate).

**Table 1:** Yeast strains used in this study.

Strain	Genotype	Source
WT GFP‐Atg8	BY4741 (MATa, his3Δ1, leu2Δ0, met15Δ0, ura3Δ0) pRS316‐GFP‐Atg8	Brachmann *et al* (1998)
*opi3∆* GFP‐Atg8	BY4741 *opi3∆*::natMX4 pRS316‐GFP‐Atg8	This study
*cho2∆* GFP‐Atg8	BY4741 *cho2∆*::natMX4 pRS316‐GFP‐Atg8	This study
WT Fba1‐GFP	BY4741 FBA1‐yeGFP::hphNT1	This study
*opi3∆* Fba1‐GFP	BY4741 FBA1‐yeGFP::hphNT1 *opi3∆*::natMX4	This study
*cho2∆* Fba1‐GFP	BY4741 FBA1‐yeGFP::hphNT1 *cho2∆*::natMX4	This study
*atg1∆* Fba1‐GFP	BY4741 FBA1‐yeGFP::hphNT1 *atg1∆*::natMX4	This study
WT (pho8Δ60 pho13Δ)	*MATaleu2–3112 trp1 ura3‐52 pho8*::*pho8*Δ*60 pho13*Δ::*LEU2*	Noda *et al* (1995)
*opi3∆* (pho8Δ60 pho13Δ)	*MATaleu2–3112 trp1 ura3‐52 pho8*::*pho8*Δ*60 pho13*Δ::*LEU2 opi3∆*::natMX4	This study
*cho2∆* (pho8Δ60 pho13Δ)	*MATaleu2–3112 trp1 ura3‐52 pho8*::*pho8*Δ*60 pho13*Δ::*LEU2 cho2∆*::natMX4	This study
*atg1∆* (pho8Δ60 pho13Δ)	*MATaleu2–3112 trp1 ura3‐52 pho8*::*pho8*Δ*60 pho13*Δ::*LEU2 Atg1∆*:: hphNT1	This study
*pep4∆*	BY4741 *pep4∆*::kanMX4	This study
*atg1∆* GFP‐Atg8	BY4741 *atg1∆*::natMX4 pRS316‐GFP‐Atg8	This study
*opi3∆atg1∆* GFP‐Atg8	BY4741 *atg1∆*::natMX4 *opi3∆*::kanMX4 pRS316‐GFP‐Atg8	This study
*atg9∆* GFP‐Atg8	BY4741 *atg9∆*::natMX4 pRS316‐GFP‐Atg8	This study
*opi3∆atg9∆* GFP‐Atg8	BY4741 *atg9∆*::natMX4 *opi3∆*::kanMX4 pRS316‐GFP‐Atg8	This study
*ypt7∆* GFP‐Atg8	BY4741 *ypt7∆*::natMX4 pRS316‐GFP‐Atg8	This study
*opi3∆* *ypt7∆* GFP‐Atg8	BY4741 *ypt7∆*::natMX4 *opi3∆*::kanMX4 pRS316‐GFP‐Atg8	This study
WT Atg5‐mNG/ sfTq2‐Atg8	w303 ura3::pUAS_F_E_C_CORE1‐OsTIR1 trp1::pATG8‐sfTq2‐Atg8‐tATG8::hphNT1 ATG5‐mNG::tSYNTH8::CgTRP1	This study
*opi3∆* Atg5‐mNG/ sfTq2‐Atg8	w303 ura3::pUAS_F_E_C_CORE1‐OsTIR1 trp1::pATG8‐sfTq2‐Atg8‐tATG8::hphNT1 ATG5‐mNG::tSYNTH8::CgTRP1 *opi3∆*::CgHIS3	This study
*ymr1∆* Atg5‐mNG/ sfTq2‐Atg8	w303 ura3::pUAS_F_E_C_CORE1‐OsTIR1 trp1::pATG8‐sfTq2‐Atg8‐tATG8::hphNT1 ATG5‐mNG::tSYNTH8::CgTRP1 ymr1∆::KlURA3	This study
*ymr1∆* *opi3∆* Atg5‐mNG/ sfTq2‐Atg8	w303 ura3::pUAS_F_E_C_CORE1‐OsTIR1 trp1::pATG8‐sfTq2‐Atg8‐tATG8::hphNT1 ATG5‐mNG::tSYNTH8::CgTRP1 *opi3∆*::CgHIS3 ymr1∆::KlURA3	This study
WT Atg1‐mNG/ sfTq2‐Atg8	w303 ura3::pUAS_F_E_C_CORE1‐OsTIR1 trp1::pATG8‐sfTq2‐Atg8‐tATG8::hphNT1 ATG1‐mNG::tSYNTH8::CgTRP1	This study
*opi3∆* Atg1‐mNG/ sfTq2‐Atg8	w303 ura3::pUAS_F_E_C_CORE1‐OsTIR1 trp1::pATG8‐sfTq2‐Atg8‐tATG8::hphNT1 ATG1‐mNG::tSYNTH8::CgTRP1 *opi3∆*::CgHIS3	This study
WT Atg9‐mNG/ sfTq2‐Atg8	w303 ura3::pUAS_F_E_C_CORE1‐OsTIR1 trp1::pATG8‐sfTq2‐Atg8‐tATG8::hphNT1 ATG9‐mNG::tSYNTH8::CgTRP1	This study
*opi3∆* Atg9‐mNG/ sfTq2‐Atg8	w303 ura3::pUAS_F_E_C_CORE1‐OsTIR1 trp1::pATG8‐sfTq2‐Atg8‐tATG8::hphNT1 ATG9‐mNG::tSYNTH8::CgTRP1 *opi3∆*::CgHIS3	This study
WT mNG‐Atg8/mScarletI‐PX^Vam7^	BY4741 trp1::pATG8‐mNeonGreen‐Atg8‐tATG8::hphNT1 vps38::NatNT2::pCUP1‐mScarletI‐Vam7(PX)‐tSYNTH13	This study
*opi3∆* mNG‐Atg8/ mScarletI‐PX^Vam7^	BY4741 trp1::pATG8‐mNeonGreen‐Atg8‐tATG8::hphNT1 vps38::NatNT2::pCUP1‐mScarletI‐Vam7(PX)‐tSYNTH13 opi3::KlURA3	This study
*ymr1∆* mNG‐Atg8/mScarletI‐PX^Vam7^	BY4741 trp1::pATG8‐mNeonGreen‐Atg8‐tATG8::hphNT1 vps38::NatNT2::pCUP1‐mScarletI‐Vam7(PX)‐tSYNTH13 ymr1::CgHIS3	This study
*opi3∆ymr1∆* mNG‐Atg8/ mScarletI‐PX^Vam7^	BY4741 trp1::pATG8‐mNeonGreen‐Atg8‐tATG8::hphNT1 vps38::NatNT2::pCUP1‐mScarletI‐Vam7(PX)‐tSYNTH13 opi3::KlURA3 ymr1::kanMX4	This study
*ymr1∆atg1∆* mNG‐Atg8/ mScarletI‐PX^Vam7^	BY4741 trp1::pATG8‐mNeonGreen‐Atg8‐tATG8::hphNT1 vps38::NatNT2::pCUP1‐mScarletI‐Vam7(PX)‐tSYNTH13 ymr1::kanMX4 atg1::CgHIS3	This study
*opi3∆ymr1∆atg1∆* mNG‐Atg8/ mScarletI‐PX^Vam7^	BY4741 trp1::pATG8‐mNeonGreen‐Atg8‐tATG8::hphNT1 ape1::NatNT2::pCUP1‐mScarletI‐Ape1‐tAPE1 opi3::KlURA3 ymr1::kanMX4 atg1::CgHIS3	This study
*ymr1∆* mNG‐Atg8/ mScarletI‐Ape1	BY4741 trp1::pATG8‐mNeonGreen‐Atg8‐tATG8::hphNT1 ape1::NatNT2::pCUP1‐mScarletI‐Ape1‐tAPE1 ymr1::CgHIS3	This study
*opi3∆ymr1∆* mNG‐Atg8/ mScarletI‐Ape1	BY4741 trp1::pATG8‐mNeonGreen‐Atg8‐tATG8::hphNT1 ape1::NatNT2::pCUP1‐mScarletI‐Ape1‐tAPE1 opi3::KlURA3 ymr1::kanMX4	This study
*ymr1∆atg1∆* mNG‐Atg8/ mScarletI‐Ape1	BY4741 trp1::pATG8‐mNeonGreen‐Atg8‐tATG8::hphNT1 ape1::NatNT2::pCUP1‐mScarletI‐Ape1‐tAPE1 ymr1::kanMX4 atg1::CgHIS3	This study
*opi3∆ymr1∆atg1∆* mNG‐Atg8/ mScarletI‐Ape1	BY4741 trp1::pATG8‐mNeonGreen‐Atg8‐tATG8::hphNT1 ape1::NatNT2::pCUP1‐mScarletI‐Ape1‐tAPE1 opi3::KlURA3 ymr1::kanMX4 atg1::CgHIS3	This study
*atg3∆* Atg5‐mNG/ sfTq2‐Atg8	w303 ura3::pUAS_F_E_C_CORE1‐OsTIR1 trp1::pATG8‐sfTq2‐Atg8‐tATG8::hphNT1 ATG5‐mNG::tSYNTH8::CgTRP1 *atg3∆*::KlURA3	This study
*opi3∆atg3∆* Atg5‐mNG/ sfTq2‐Atg8	w303 ura3::pUAS_F_E_C_CORE1‐OsTIR1 trp1::pATG8‐sfTq2‐Atg8‐tATG8::hphNT1 ATG5‐mNG::tSYNTH8::CgTRP1 *opi3∆*::CgHIS3 *atg3∆*::KlURA3	This study
WT BFP‐Ape1	BY4741 trp1::pATG8‐mNG‐Atg8‐tATG8::hphNT1 pDP105 (pRS315[pAPE1‐TagBFP‐Ape1‐tCYC1::pCUP1‐Ape1‐tAPE1])	This study
*opi3∆* BFP‐Ape1	BY4741 opi3::natMX4 trp1::pATG8‐mNG‐Atg8‐tATG8::hphNT1 pDP105 (pRS315[pAPE1‐TagBFP‐Ape1‐tCYC1::pCUP1‐Ape1‐tAPE1])	This study

### Culture preparation, protein extraction, and Western blotting

Yeast strains were maintained as colonies on agar plates (4°C) or grown as cultures in liquid medium (30°C, 180–220 rpm). Cultures grown overnight to saturation from colonies were diluted in the morning 1:10, grown for 5–6 h, followed by another dilution and overnight logarithmic growth (to 0.7–1 OD_600_). Cells were treated with rapamycin by direct addition to culture, or starved for nitrogen by washing (3,200 rpm, 2 min, DDW) and resuspending in SD‐N (1 OD_600_). A 1 ml samples were taken at indicated times, centrifuged (12,000 rpm, 2 min), and pellets were frozen. Resuspended pellets (100 μl of DDW) were treated with 0.2 M NaOH (100 μl, 5 min, room temperature), centrifuged (13,000 rpm, 3 min), resuspended in Laemmli sample buffer (70 μl 2×), and heated (65°C, 5 min). Proteins were analyzed on 10% SDS‐PAGE gels and transferred to a methanol‐activated PVDF membrane (Millipore) at 250 mA for 110 min. Membranes were blocked (5% (w/v) non‐fat milk powder in PBS, 30 min), incubated with primary antibody (3 h, or overnight at 4°C), washed (PBS with 0.1% (v/v) Tween‐20 (PBST)), incubated with HRP‐conjugated secondary antibody (in 2.5% (w/v) non‐fat milk powder in PBST, 40 min), washed, and proteins were visualized using the EZ‐ECL western blotting detection reagent (Biological Industries), in accordance with manufacturer's protocol.

### Reagents, antibodies, and plasmids

All reagents were from Sigma, except where indicated, with the following stock compositions. Yeast nitrogen base (Difco); Choline (1 M stock in DDW); Rapamycin (GoldBio; primary stock solution: 2.5 mg/ml in 100% ethanol, and secondary stock of 1 mg/ml in 90% ethanol+10% Tween‐20 (v:v), final 200 ng/ml). Copper sulfate (used at 500 μM from a 0.5 M stock in DDW). Potassium Permanganate (Dissolved in DDW), S‐1 (or Dimethylethanolamine)—Ted‐Pella−18315. BEEM embedding capsules size 00, EMS, 70010‐B. Proteinase K (stock 2 mg/ml, final 100 mg/ml (diluted in PS200)); Triton X‐100 (stock 40% (w/v), final 0.4% (w/v)); DTT (Bio Basic Inc); PMSF (200 mM in EtOH); Z1000 Zymolyase 20T (US biological); Sodium‐Sulfate (BDH); Mouse anti‐GFP (MBL); mouse anti‐Pgk1 (Abcam); Rabbit anti‐Ape1 (polyclonal antibodies prepared by using two synthetic peptides that were conjugated to keyhole limpet hemocyanin (KLH) and then injected into rabbits to produce an anti‐Ape1 antiserum that recognizes both precursor and mature forms); Anti‐cpy1 polyclonal antibody (obtained from Prof. Howard Riezman); pRS316‐GFP‐Atg8 (expressed under the promoter of Atg8, kind gift from Prof. Yoshinori Ohsumi); pDP105 (expressing TagBFP‐Ape1 under the native Ape1 promoter and Ape1 under the pCUP1 promoter, kind gift from Prof. Claudine Kraft).

### Pho8∆60 assay

The measurement of non‐selective autophagy was performed by the Pho8∆60 assay as previously described (Noda & Klionsky, 2008).

### Proteinase K protection assay

Cells expressing GFP‐Atg8 were grown in SD‐URA (0.7–0.8 OD 600/ml, 30 ml cultures) and starved (as above, 4 h). After centrifugation (1,000 *g*, 2 min), cells were pretreated with zymolyase for 30 min at 30°C, centrifuged (2000 *g*, 10 min), resuspended in homogenization buffer (10 mM Tris–HCl, pH 7.4 and 0.25 M sucrose, 4 pellet volumes) supplemented by PMSF (1 mM) and protease inhibitors cocktail (Roche) and homogenized on ice with Kontes Glass Co (Sigma) tissue grinder (30 strokes). Unbroken cells and nuclei were removed (700 *g*, 5 min, 4°C) and equal amounts of homogenate were ultracentrifuged (TLA 120.2 rotor, 90,000 rpm, 30 min, 4°C) to separate cytosol and membrane fractions, and the pellets were resuspended in homogenization buffer. Each fraction was then divided into equal volumes and incubated (30 min) for treatments with proteinase K (10 μg/ml, Merck, 1245680) and/or Triton X‐100 (0.4% (v/v)). Treatments were terminated by addition of PMSF (1 mM, 10 min on ice), and proteins were precipitated by 10% TCA, washed by analytical acetone, boiled in sample buffer and immunoblotted.

### FM4‐64 staining

A 1 μl of 1.6 mM FM4‐64 (Invitrogen) was added to 1 ml of culture at 30°C for 10–20 min, before collection (3,200 rpm, 2 min), wash (DDW), and resuspension in SD (for growth conditions) or SD‐N (for starvation), and imaging.

### Microscopy

Slides were prepared with 7 μl of culture on cover glass (Precision Cover Glass #1.5H, 13 mm, Cat. 0117530, Marienfeld), covered by Zeiss cover slip (high precision, 22 × 22 mm, 170, No.1.5H, Zeiss) secured with Vaseline. Cells were visualized by Zeiss confocal microscope LSM 880, AxioObserver, using X63/1.4 DIC M27 immersion oil objective. Beam splitter: MBS488, MBS‐Invis: plate, DBS1: Mirror. Laser 488 nm. Channels used were: Ex 488, emission: 536 (499–574); Excitation: 561, emission: 682 (606–758); T PMT. Pinhole: 0.66 AU. Line averaging: 4. Airyscan images were obtained by the superresulution Airyscan mode, filters: BP 495–550 + LP 570, beam filters MBS: MBS 488/561, MBS_InVis: Plate, DBS1: Plate. Laser 488, images were processed by the ZEN program. Alternatively, cells were visualized in a widefield Zeiss AxioObserver.Z1/7 microscope, using Plan Apochromat 100×/1.46 oil DIC (UV) M27 immersion oil objective, coupled with 1.6× tube lens. Images obtained from whidefield microscopy were smoothed by Gaussian filter with factor 1.3, and images in Figs 5C–F, 6A and B, 7A, and EV2C, were deconvolved (constrained iterative, 1.4 NA).

### Correlative light and electron microscopy (CLEM)

Cells (0.7–0.8 OD 600/ml, 30 ml cultures) were washed twice with DDW (800 *g*, 2–5 min), resuspended in SD‐N and incubated for 4 h, followed by filtration (0.45 μm membrane filter) and collection of pellets by a silicon spatula. Each pellet was placed in an aluminum disc with a depression of 100 μm and outer diameter of 3 mm (Engineering Office M. Wohlwend GmbH). It was covered with a matching flat disc. The sandwiched sample was high‐pressure frozen using an EM ICE high pressure freezing device (Leica Microsystems). The frozen samples were dehydrated by freeze‐substitution in an AFS2 freeze‐substitution device (Leica Microsystems, Vienna Austria) in anhydrous acetone containing 0.1% uranyl acetate, embedded in Lowicryl HM20 acrylic resin (Electron Microscopy Sciences, USA) and polymerized in UV (Kukulski *et al*, 2012).

Sections with thickness of 120 nM were cut with a diamond knife (Diatome) using a UC7 ultramicrotome (Leica Microsystems). Sections were mounted on 200 mesh Formvar coated nickel grids and labeled with DAPI (1 μg/ml, 20 min). Widefield fluorescence images were taken to identify yeast cells with GFP‐Atg8 using VUTARA SR352 system (Bruker) with 1.3 NA 60× silicon oil immersion objective (Olympus). Imaging was performed using 405 nm and 488 nm excitation lasers in the presence of an imaging buffer (7 μM glucose oxidase (Sigma), 56 nM catalase (Sigma), 2 mM cysteamine (Sigma), 50 mM Tris–HCl pH 8, 10 mM NaCl, 10% glucose).

The same grids were washed with DDW, double stained with 2% uranyl acetate and Reynolds lead citrate and viewed using a Tecnai F20 transmission electron microscope (Thermo Fisher Scientific) operating at 200 kV and equipped with a US400 CCD camera (Gatan). Acquisition of large regions for orientation and for correlating between images acquired by light microscopy and regions observed in the TEM was done using the SerialEM program (Mastronarde, 2005). Initial registration between LM and TEM regions was conducted using the grid's mesh corners as registration points.

DAPI staining and GFP‐Atg8‐labeled regions were used to identify relevant cells in the section and regions of interest for TEM investigation. Fine‐tuning of the correlation was based on nuclei marked with DAPI, which were also easily identified in the TEM. Thus, nuclei were used as fiducial markers to overlay the fluorescence image and the TEM image in high accuracy. Overlay of fluorescent images (GFP‐Atg8 only after validation of the area with the DAPI marker) on TEM image was done by Adobe Photoshop.

### Quantifications and statistical analysis

Quantifications of western blot measurements and autophagic structure diameter were done by ImageJ. For the quantifications of free GFP, Ape1 maturation and pho8 activity, WT were normalized to 1. The background values for the experiments with Pho8 and Fba1 were obtained from the Δ*atg1* mutants. The values obtained for Statistical analysis was performed using Graphpad Prism. Relative abundance of phospholipids was measured as percentage of each phospholipid from the total phospholipid amount.

### Immunoprecipitation of GFP‐Atg8 membranes

Cells (0.7–0.8 OD 600/ml, 50 ml cultures) expressing GFP‐Atg8 were grown in SD‐URA medium and shifted to SD‐N for 3 h. Yeast pellets were resuspended in 4 pellet volumes of homogenization buffer (10 mM Tris–HCl, pH 7.4 and 0.25 M sucrose) supplemented by PMSF (2 mM) and protease inhibitors cocktail (Roche) and homogenized by beating with glass beads (Sigma) using Bullet Blender Storm 24 (Next Advance, 4× [45 s ON, 1 min OFF], 4°C). Unbroken cells and nuclei were removed (12,000 *g*, 5 min, 4°C) and equal amounts of homogenate were ultracentrifuged (as above) to obtain membrane fraction. Pellets were resuspended in homogenization buffer and incubated with GFP‐Trap magnetic beads (Chromotek, 3 h, 4°C) and washed 3 times with washing buffer (10 mM Tris–HCl, pH 7.5, 150 mM NaCl, 0.5 mM EDTA). To validate immunoprecipitation, the proteins were eluted by 30 μl Laemmli sample buffer (65°C, 5 min) and subjected to western blot analyses. After validation, similarly treated beads (without elution) were subjected to lipidomic analysis.

### Lipidomics

For whole cell membranes, cells expressing GFP‐Atg8 were grown in SD‐URA medium and shifted to SD‐N for 4 h. Yeast pellets were taken before and after starvation, immediately frozen at −80°C and sent in dry ice for an analysis by quantitative shotgun lipidomics by Dr. Xianlin Han, as described (Han & Gross, 2005). In brief, individual yeast pellets were homogenized (Potter‐Elvehjem tissue grinder in 0.5 ml 0.1× PBS). BCA protein assay on individual homogenates was used to measure protein concentration. An aliquot of homogenate was transferred to a disposable glass test tube. A mixture of lipid internal standards for quantification of all reported lipid classes was added to the tube based on the tissue protein content (Wang *et al*, 2017). Lipid extraction was performed by a modified Bligh and Dyer method as previously described (Wang & Han, 2014). The entire lipid extracts were flushed with N_2_, capped, and stored at −20°C.

For ESI‐MS analysis after direct infusion, lipid extracts were further diluted to a final concentration of ~ 500 fmol/μl, and the mass spectrometric analysis was performed in a Q‐Exacutive mass spectrometer (Thermo, San Jose, CA) for cardiolipin, lyso‐cardiolipin, and PG analysis and in a TSQ Altis mass spectrometer (Thermo, San Jose, CA) for other lipids. Both instruments were equipped with an automated nanospray device (TriVersa NanoMate, Advion Bioscience Ltd., Ithaca, NY) to ionize the lipid species by nano‐ESI and operated with the Xcalibur software as previously described (Han *et al*, 2008; Yang *et al*, 2009). Identifications and quantifications of all lipid species of interest were performed using an in‐house automated software program (Yang *et al*, 2009), following the principles for quantification by mass spectrometry as previously described in detail (Yang & Han, 2011). Fatty acyl chains of lipids were identified and quantified by neutral loss scans or precursor ion scans of corresponding acyl chains, and calculated using the same in‐house software program (Yang *et al*, 2009). All lipid levels were normalized to the sample protein content.

For Atg8 membranes, lipid species were analyzed by using multidimensional mass spectrometry‐based shotgun lipidomics approach (Han, 2017). The immunoprecipated beads were transferred into a glass tube. Then, a pre‐mixed lipid internal standard were added to each tube according to their protein content. Lipid extraction was performed using a modified Bligh and Dyer method (Wang & Han, 2014). The lipid extract was dissolved in chloroform:methanol (1:1, v:v) at a ratio of 400 μl/mg protein for storage.

For shotgun lipidomics, the lipid extract was further diluted to a total lipid concentration of ~ 2 pmol/μl. The mass spectrometric analysis was performed on a triple quadrupole mass spectrometer (TSQ Altis, Thermo Fisher Scientific, San Jose, CA) and a hybrid quadrupole‐Orbitrap mass spectrometer (Q‐Exactive, Thermo Fisher Scientific, San Jose, CA), both equipped with an automated nanospray ion source device (TriVersa NanoMate, Advion Bioscience Ltd., Ithaca, NY) as described previously (Han *et al*, 2008).

The data processing and analysis was performed based on the principles of shotgun lipidomics such as ion peak selection, baseline correction, isotope effect correction, etc. (Yang & Han, 2011; Han, 2017; Wang *et al*, 2017). The final lipidomics result were normalized to the protein content (pmol/mg protein).

### Transmission electron microscopy (TEM)

Cells expressing GFP‐Atg8 were grown in SD‐URA medium and starved (3 h, as above). Samples were collected and processed for EM as previously described (Griffith *et al*, 2008). Briefly, 10–15 OD_600_ cell equivalent were centrifuged (3,000 rpm, 5 min at room temperature), washed (10 ml of DDW), resuspended in freshly prepared ice‐cold 1.5% (w/v) KMnO_4_ (3 ml) and split into two microcentrifuge tubes, covered entirely with ice‐cold 1.5% KMnO_4_, incubated (in rotation, 30 min at 4°C), centrifuged (4,000 rpm, 3 min at 4°C), resuspended in KMnO_4_ (1.5 ml ice‐cold 1.5% (w/v)), and incubated (as above or overnight), followed by washing in DDW (5 × 1 ml, 5,000 rpm, 3 min). Cells were then step‐wise dehydrated in acetone (10, 30, 50, 70, 90, 95%; each 1 ml at least 20 min, at room temperature, then 5,000 rpm, 4 min), treated with freshly made Spurr's resin (33% (w/v), in rotation at least 1 hat room temperature), centrifuged (5,000 rpm, 3 min), treated again with Spurr's resin (100% (w/v), rotated overnight at room temperature), centrifuged (5,000–7,000 rpm, 3–5 min), and incubation with fresh Spurr resin was repeated (5–6 h at room temperature). Finally, samples were transferred to conic embedding capsules, centrifuged (5,000–7,000 rpm, 3–5 min), filled with Spurr's resin (100% (w/v)) and polymerized (60°C at least 3 days). Cutting and imaging were done as above for CLEM.

## Author contributions

**Zvulun Elazar:** Conceptualization; data curation; supervision; funding acquisition; investigation; writing – original draft; project administration; writing – review and editing. **Alexandra Polyansky:** Conceptualization; data curation; investigation; writing – original draft; writing – review and editing. **Oren Shatz:** Investigation; methodology; writing – original draft; writing – review and editing. **Milana Fraiberg:** Data curation; investigation; methodology. **Eyal Shimoni:** Visualization; methodology. **Tali Dadosh:** Visualization; methodology. **Fulvio M Reggiori:** Visualization; methodology. **Xianlin Han:** Formal analysis; project administration. **Muriel Mari:** Visualization; methodology. **Chao Qin:** Investigation; methodology.

## Disclosure and competing interests statement

The authors declare that they have no conflict of interest.

## Supporting information



Appendix

Expanded View Figures PDF

Movie EV1

Movie EV2

Movie EV3

Movie EV4

Movie EV5

Source Data for Expanded View

Source Data for Figure 1

Source Data for Figure 3

PDF+

## Data Availability

This study includes no data deposited in external repositories.
