# Supplementary material for: Phospholipid imbalance impairs autophagosome completion
Source: EMBO J. 2022 Oct 27;41(23):e110771. doi: 10.15252/embj.2022110771 (PMC9713711; doi:10.15252/embj.2022110771)
Supplement: Supplementary file 1 — Appendix [file EMBJ-41-e110771-s005.pdf]

Table of contents to appendix figure S1

A- Acyl-chain length and composition of PG of WT and Δ*opi3* cells…………..…......2
B- Acyl-chain length and composition of PE of WT and Δ*opi3* cells………….............2
C- Acyl-chain length and composition of PA of WT and Δ*opi3* cells………….............2
D- Acyl-chain length and composition of PS of WT and Δ*opi3* cells……………….....2
E- Acyl-chain length and composition of PI of WT and Δ*opi3* cells…………..........3
F- Acyl-chain length and composition of PMME of WT and Δ*opi3* cells ……............3
G- Acyl-chain length and composition of PC of WT and Δ*opi3* cells………………....3
H- Schematics of the procedure for lipidomic analysis of autophagic membranes…...4
I- Acyl-chain length and composition of PI of Δ*ypt7* and Δ*opi3*Δ*ypt7* cells…………..4
J- Acyl-chain length and composition of PS of Δ*ypt7* and Δ*opi3*Δ*ypt7* cells………….4
K- Acyl-chain length and composition of PG of Δ*ypt7* and Δ*opi3*Δ*ypt7* cells………...4
L- Acyl-chain length and composition of PG of Δ*ypt7* and Δ*opi3*Δ*ypt7* cells…….....4
Legend to appendix figure S1…………………………………………………….5

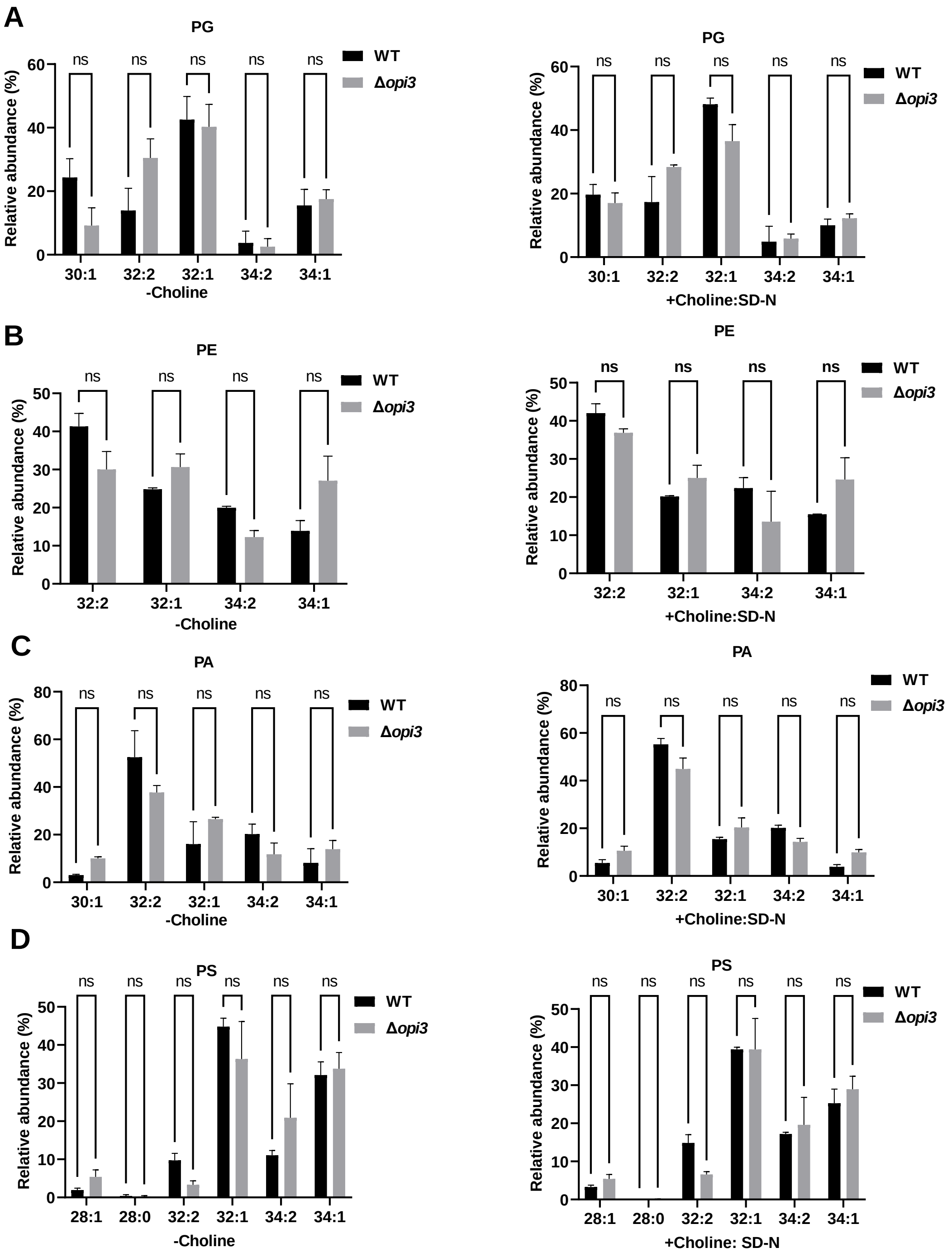

Polyansky et al., appendix Figure S1

E

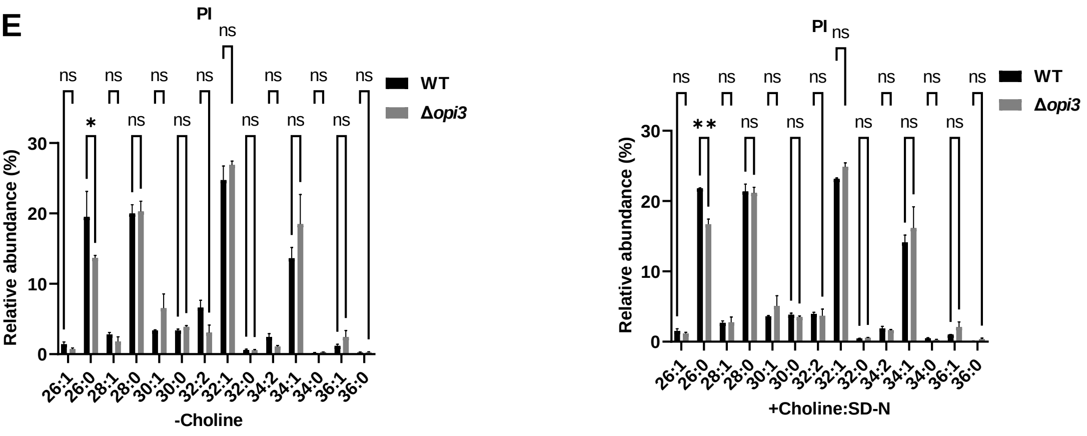

F

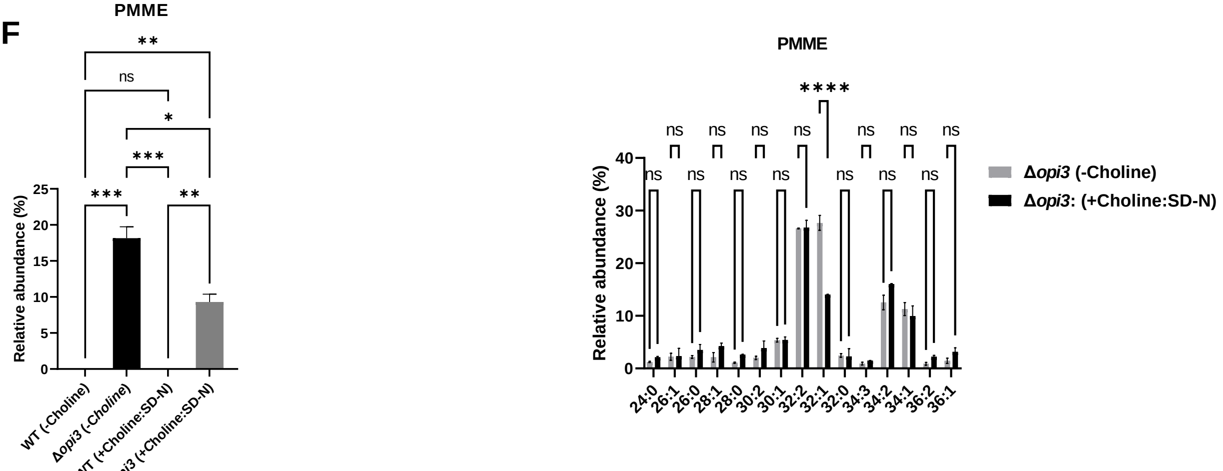

G

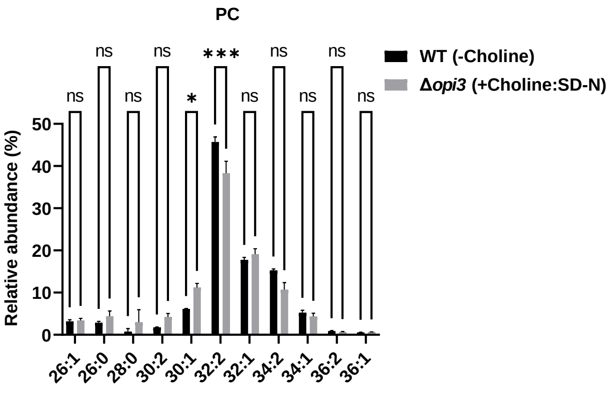

Polyansky et al., appendix Figure S1

H Yeast cells → Lysis → Pellet → IP GFP magnetic beads → Lipidomics of Atg8 membranes

I

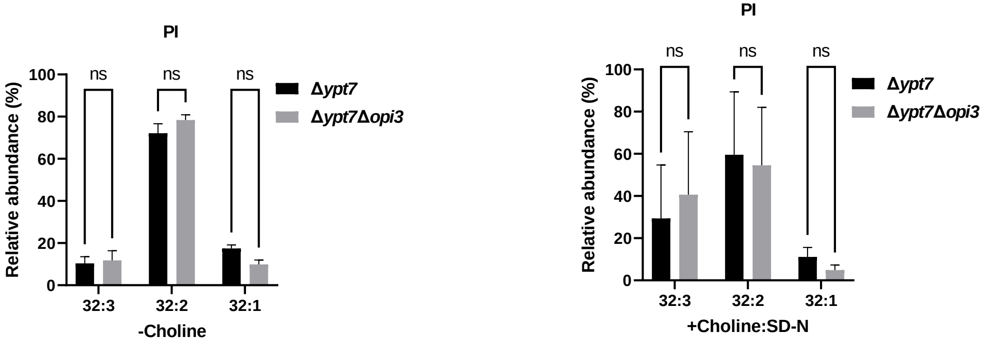

J

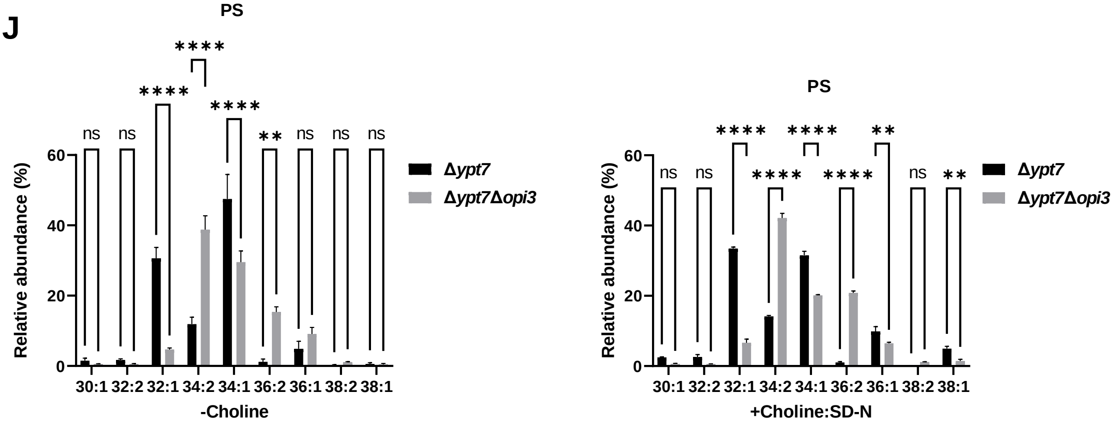

K

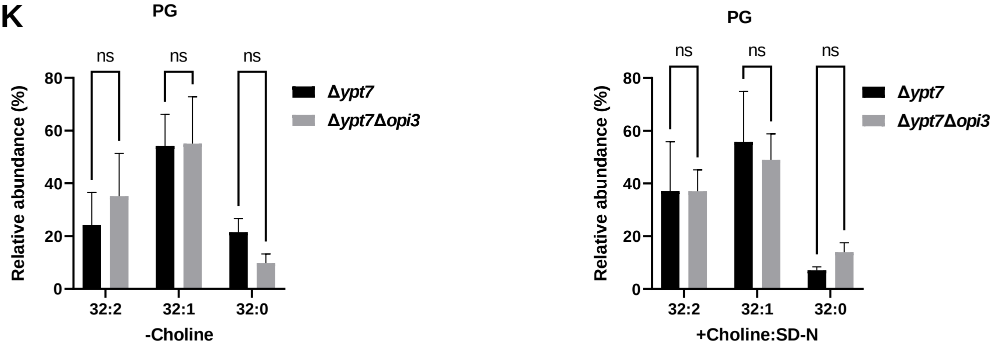

L

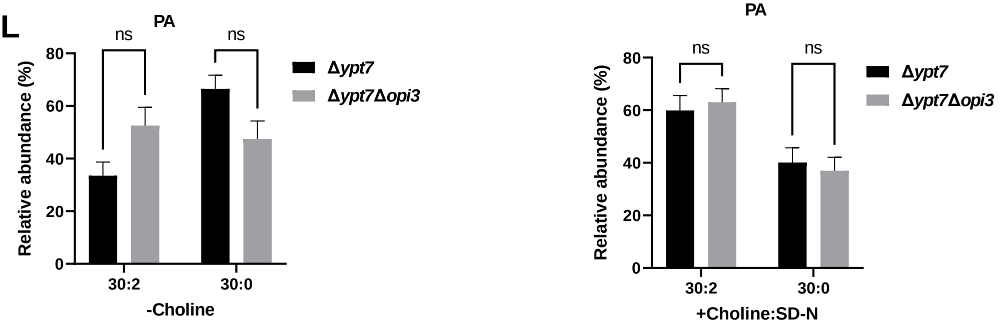

Polyansky et al., appendix Figure S1

Appendix figure S1: Addition of choline to Δ*opi3* cells specifically restores PC levels

A-G. Phospholipids species of WT and Δ*opi3* cells. Shown are acyl-chain length and composition of PG (A), PE (B), PA (C), PS (D), PI (E), PMME (F), and PC (G). Cells were grown in SD-URA, and starved for 4 h in SD-N, with choline (1 mM) supplementation excluded (-choline) or during starvation (+choline: SD-N) as indicated, harvested and analyzed by shotgun lipidomics. Statistical analysis was done by Anova multiple comparisons test- Sidak's (****, $p \leq 0.0001$, **, $p \leq 0.005$, *, $p \leq 0.001$, ns- not significant), error bars represent SEM (n=3, except for PE, PMME and WT with choline, n=2). For PMME (F- left panel)- Tucky's multiple comparison test.

H. Schematics of the procedure for lipidomic analysis of autophagic membranes. GFP-Atg8 expressing cells were lysed, Atg8-positive membranes were isolated from membrane fraction by GFP magnetic beads, and analyzed by shotgun lipidomics for lipid composition.

I-L: Phospholipids species of Δ*ypt7* and Δ*ypt7*Δ*opi3* cells. Shown are acyl chain length and composition of PI (I), PS (J), PG (K), PA (L). Cells expressing GFP-Atg8 were grown to log phase in SD-URA, and shifted to starvation medium. Cells were harvested after 3 h of starvation, lysed and membrane fraction was immunopercipitated with GFP-magnetic beads. Atg8 enriched membranes were eluted and analyzed by shotgun lipidomics. Statistical analysis was done by Anova multiple comparisons test- Sidak's (****, $p \leq 0.0001$, **, $p \leq 0.005$, ns- not significant), error bars represent SEM of 3 independent experiments.
