# Supplementary material for: Phospholipid imbalance impairs autophagosome completion
Source: EMBO J. 2022 Oct 27;41(23):e110771. doi: 10.15252/embj.2022110771 (PMC9713711; doi:10.15252/embj.2022110771)
Supplement: Supplementary file 2 — Expanded View Figures PDF [file EMBJ-41-e110771-s004.pdf]

Expanded View Figures

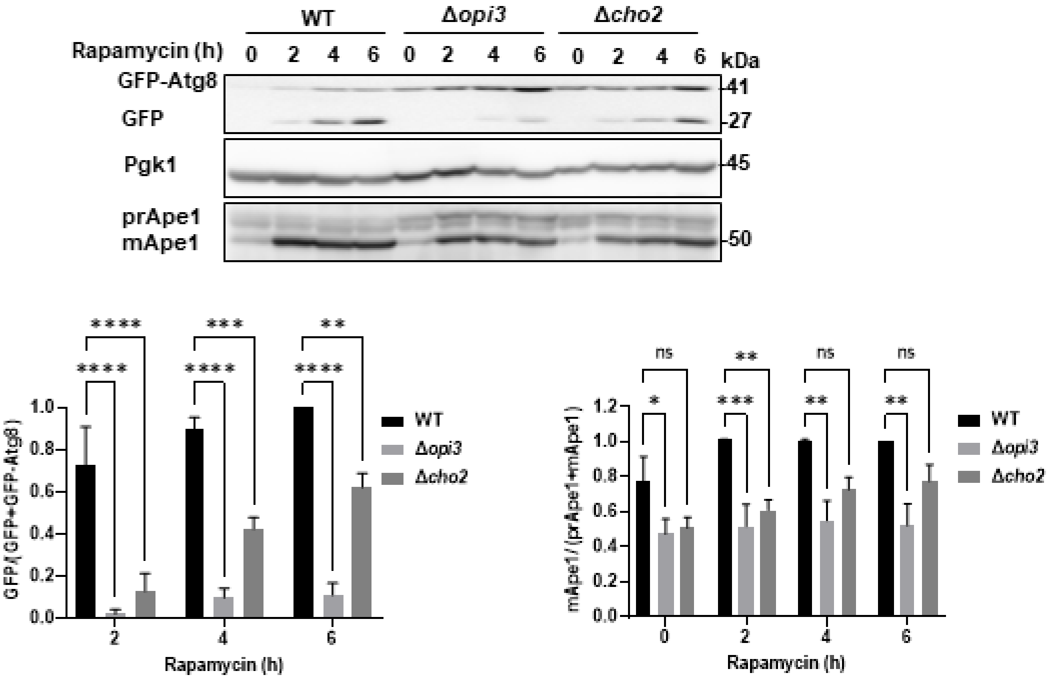

Figure EV1. Rapamycin induced autophagy is inhibited in PC deficient strains.

WT, Δ*opi3* and Δ*cho2* cells expressing GFP-Atg8 were grown to log phase in SD-URA and treated with rapamycin (200 ng/ml) for the indicated durations. Cells were harvested at indicated time points and subjected to western blotting (left panel). Pgk1 was monitored as a loading control. Bottom left panel- Autophagic activity was quantified during indicated time points of starvation, by calculating the ratio of free GFP to total GFP (GFP-Atg8 + free GFP). Statistical analysis was done by ANOVA multiple comparison test-Dunnett's, compared with WT (**$P \leq 0.005$, ***$P \leq 0.005$, ****$P \leq 0.0001$), error bars represent SEM of at least 3 independent experiments. Bottom right panel-Ape1 maturation was quantified by measuring the mApe1 level out of the total Ape1 amount, during indicated time points. Statistical analysis was done by ANOVA multiple comparison test-Dunnett's, compared with WT (*$P \leq 0.05$, **$P \leq 0.005$, ***$P \leq 0.005$, ns, not significant), error bars represent SEM of at least 3 independent experiments.

Source data are available online for this figure.

© 2022 The Authors

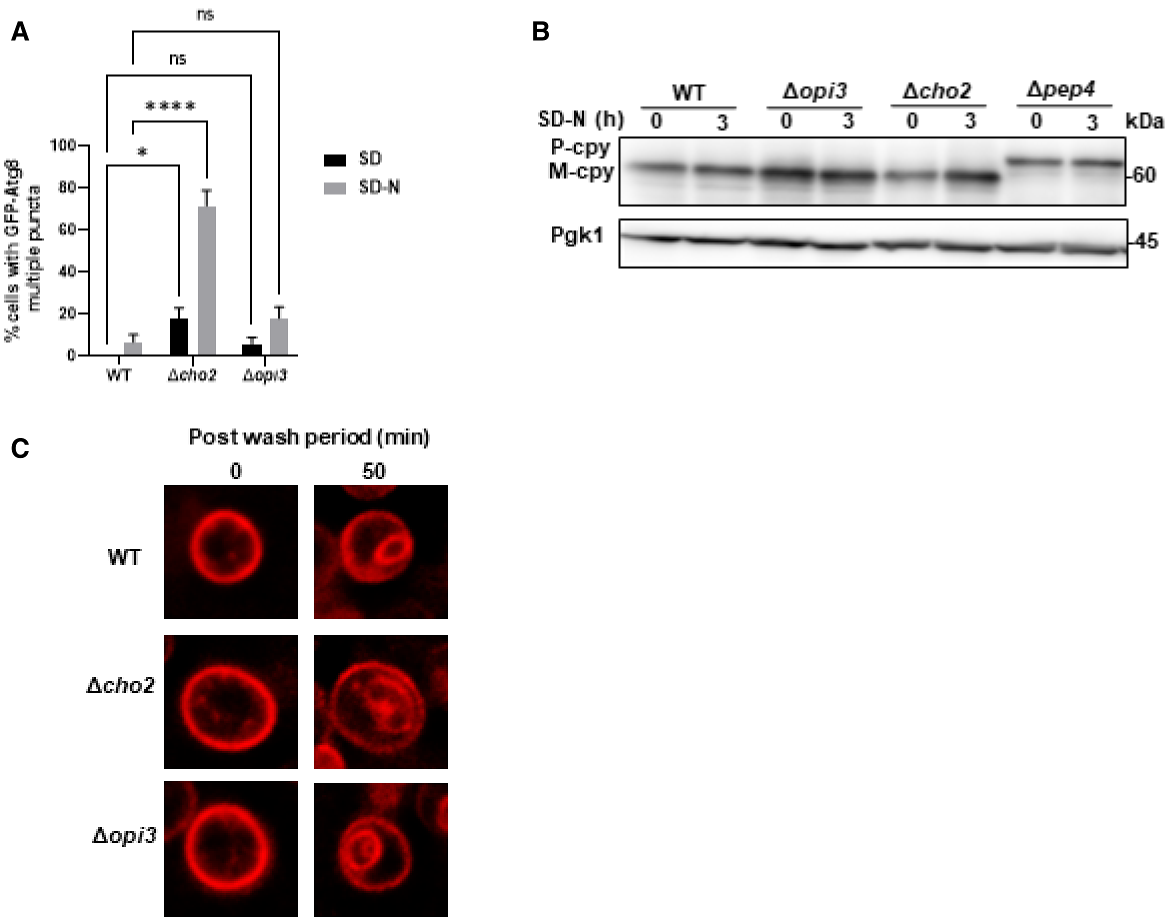

Figure EV2. PC deficiency specifically compromises the autophagic pathway.

A Quantification of cells with multiple GFP-Atg8 puncta during SD and SD-N. WT, Δ*opi3*, Δ*cho2* cells expressing GFP-Atg8 were grown to log phase in SD-URA and shifted to SD-N for 3 h. Percentage of cells with more than two GFP-Atg8 puncta per cell GFP-Atg8 puncta was counted. Statistical analysis was done by ANOVA multiple comparisons test-Sidak's (**P* ≤ 0.05, *****P* ≤ 0.0001, ns, not significant), error bars represent SEM of 3 independent experiments. Number of cells analyzed for each strain and condition, SD: WT (*n* = 446), Δ*opi3* (*n* = 272), Δ*cho2* (*n* = 272), SD-N: WT (*n* = 398), Δ*opi3* (*n* = 213), Δ*cho2* (*n* = 224).

B CPY maturation assay: precursor CPY (pCPY) is transported to the vacuole and processed into a mature form (mCPY) in the vacuolar lumen. WT, Δ*opi3* and Δ*cho2* cells, as well as Δ*pep4* cells as negative control for CPY maturation, all expressing GFP-Atg8, were grown to log phase in SD-URA, and shifted to SD-N for 3 h. Cells were harvested at the indicated time points and subjected to western blotting (Fig 2B). Pgk1 was monitored as loading control.

C WT, Δ*opi3* and Δ*cho2* cells were grown to log phase in SD medium, stained for 30 min on ice with FM4-64, washed 3 times with cold SD, and observed at indicated time points after wash by widefield microscopy (Fig 2C). Scale bar 1 μm.

Source data are available online for this figure.

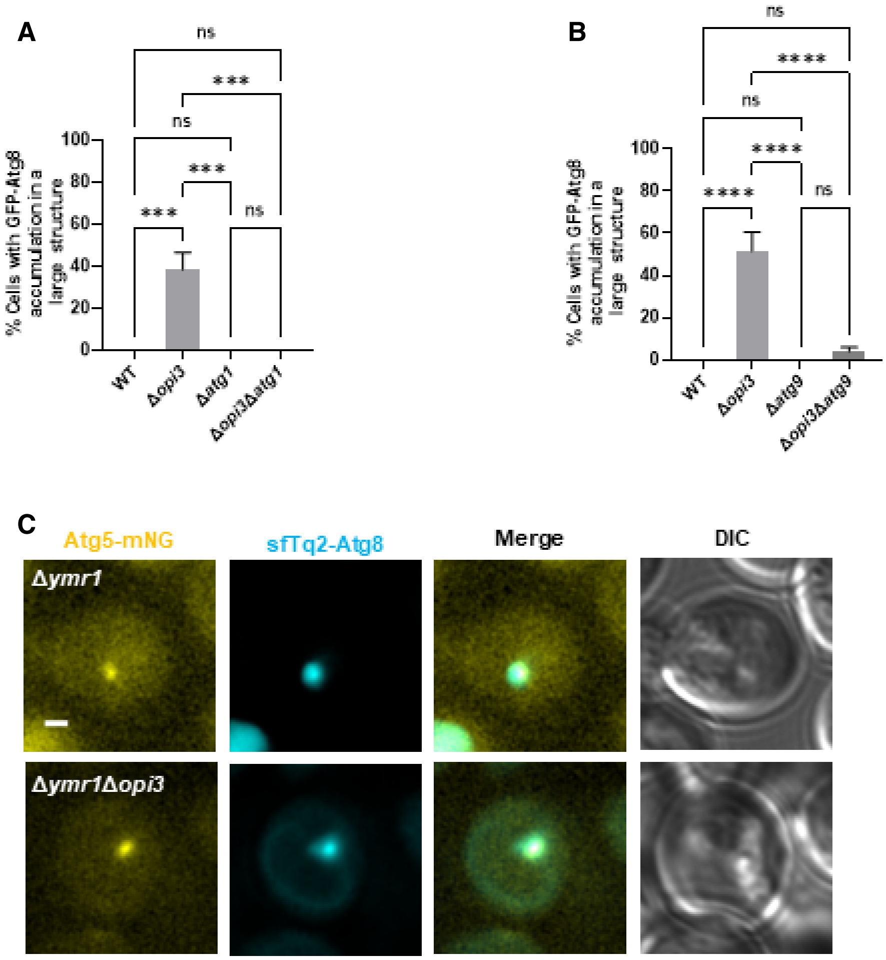

Figure EV3. Phospholipid imbalance leads to accumulation of *bona fide* autophagic structures.

A, B Quantification of imaged data shown in Fig 5A and B, respectively, of cells with GFP-Atg8 accumulation in large structures. Statistical analysis was done by ANOVA multiple comparisons test-Tukey's (****$P \leq 0.0001$, ns, not significant) error bars represent SEM of least 3 independent experiments. Number of cells analyzed for each strain. Panel (A): WT ($n = 298$), Δ*opi3* ($n = 510$), Δ*atg1* ($n = 179$), Δ*opi3*Δ*atg1* ($n = 177$), Panel (B): WT ($n = 349$), Δ*opi3* ($n = 564$), Δ*atg9* ($n = 275$), Δ*opi3*Δ*atg9* ($n = 253$).

C Representative images of mNG-tagged Atg5 colocalized with sfTq2-Atg8 on the background of Δ*ymr1*. WT and Δ*opi3* cells were grown to log phase in SD medium, and shifted to SD-N. Images were taken during SD-N (1–3 h) by widefield microscopy. Scale bar 1 μm.

 © 2022 The Authors

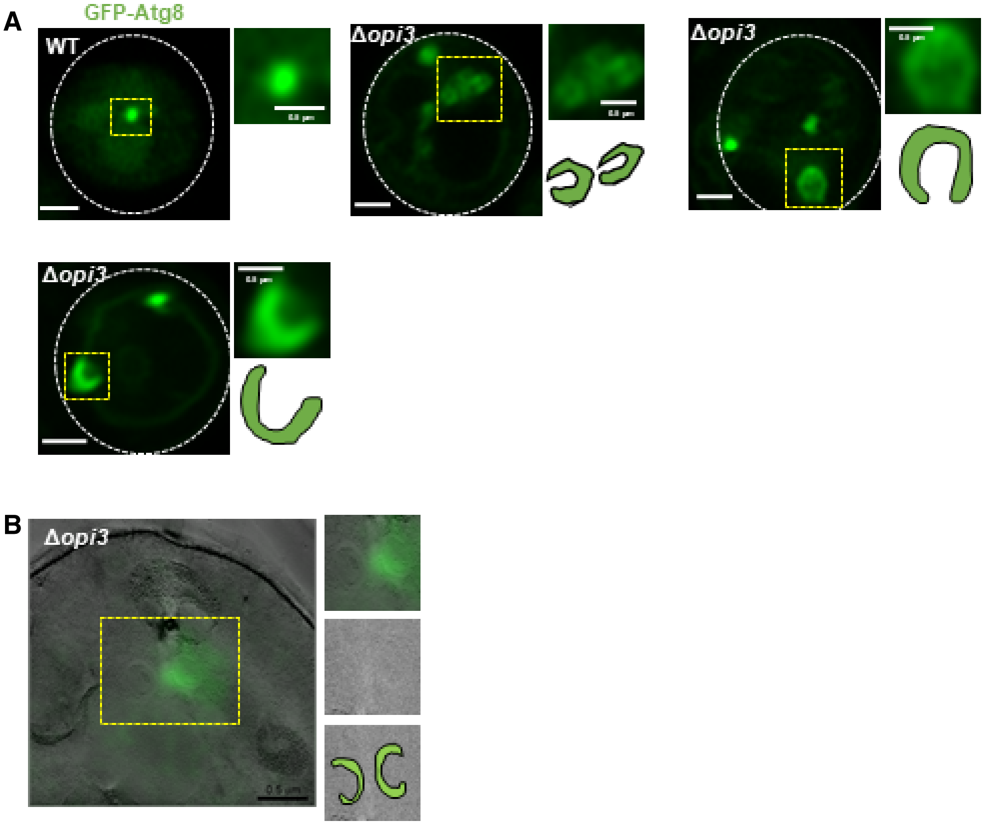

Figure EV4. Phospholipid imbalance leads to accumulation of elongated unsealed phagophores.

A Representative images of autophagic structures in WT and Δ*opi3* cells expressing GFP-Atg8. Cells were grown to log phase in SD-URA medium, shifted to SD-N for 3 h and observed by Airyscan microscopy. White dashed lines indicate cell boundaries, yellow dashed area indicate autophagic structures. For each cell—whole cell (left), magnification of yellow dashed area (top right) and schematic representation (bottom right). Scale bar 1 μm, 0.5 μm (enlarged images).

B Representative CLEM image of Δ*opi3* cells expressing GFP-Atg8, grown to log phase in SD-URA medium and shifted to SD-N for 3 h. Cells were harvested, deep frozen and processed for CLEM (left), V-vacuole. For each cell—magnification of yellow dashed area of GFP-Atg8 positive phagophore (top right), TEM image only (middle right), schematic representation of phagophores (bottom right). Scale bar 1 μm (left).

© 2022 The Authors

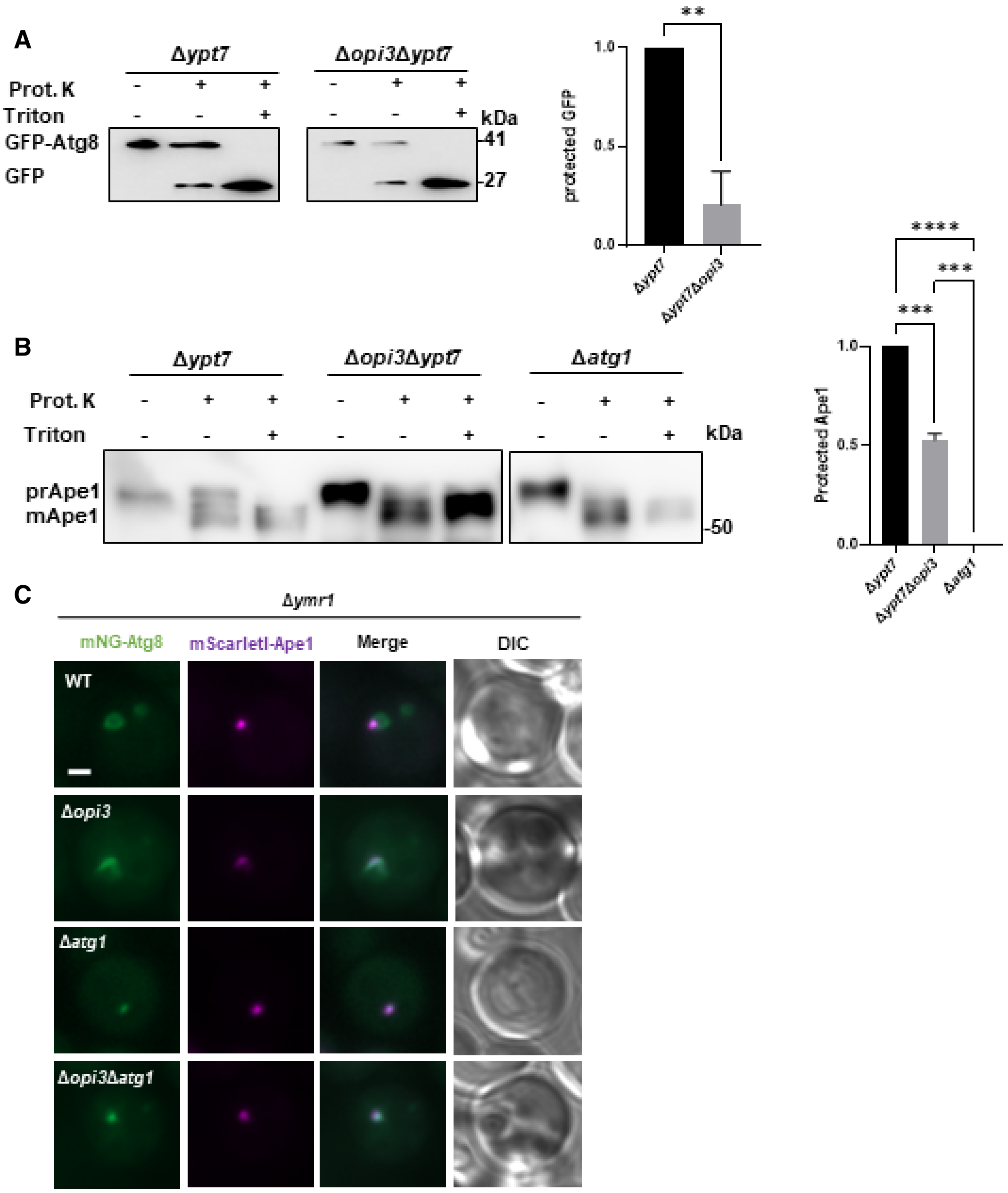

Figure EV5. Phospholipid balance promotes succession of autophagosome completion.

A, B *Δypt7* and *Δopi3Δypt7* mutant cells expressing GFP-Atg8 were grown to log phase in SD-URA medium and shifted to SD-N for 3 h, cell lysates were subjected to protease protection assay combined with immunoblot analysis (left panel). GFP-Atg8 processing (A) or Ape1 Processing (B) were determined with or without addition of proteinase K (Prot. K), in the presence or absence of detergent (Triton). Protection of GFP-Atg8/ ape1 may be assessed when proteinase K is added without detergent. Right panel (A)- protected GFP-the ratio of GFP-Atg8 to total GFP (GFP-Atg8 + free GFP). Statistical analysis was done by Student's *t*-test (paired, two tailed; $^{**}P \leq 0.005$), error bars represent SEM of at least 3 independent experiments. Right panel (B)- protected Ape1-the ratio of prApe1 to total Ape1 (PrApe1 + mApe1). Statistical analysis was done by ANOVA multiple comparison Tukey's test ($^{****}P \leq 0.0001$, $^{***}P \leq 0.001$).

C Representative images of mNG-tagged Atg8 colocalized with mScarletI-Ape1 expressed under the CUP1 promoter on the background of *Δymr1*. WT, *Δopi3*, *Δatg1* and *Δopi3Δatg1* cells were grown to log phase in SD medium in the presence of 10 μM copper sulfate, and shifted to SD-N in the presence of 10 μM copper sulfate. Images were taken during SD-N (1–3 h) by widefield microscopy. Scale bar 1 μm.

Source data are available online for this figure.

© 2022 The Authors
