# Supplementary material for: Phospholipid imbalance impairs autophagosome completion
Source: EMBO J. 2022 Oct 27;41(23):e110771. doi: 10.15252/embj.2022110771 (PMC9713711; doi:10.15252/embj.2022110771)
Supplement: Supplementary file 3 — Movie EV1 [file EMBJ-41-e110771-s008.zip › movie 1.docx]

Movie1:

Tomogram movie of Δ*opi3* CLEM image (Fig. 6D-E), focused on the phagophore, thickness of 120 nm, scale bar 500 nm.
