# Supplementary material for: Phospholipid imbalance impairs autophagosome completion
Source: EMBO J. 2022 Oct 27;41(23):e110771. doi: 10.15252/embj.2022110771 (PMC9713711; doi:10.15252/embj.2022110771)
Supplement: Supplementary file 4 — Movie EV2 [file EMBJ-41-e110771-s006.zip › Movie 2/movie 2.docx]

Movie 2:

Δ*ypt7* cells expressing GFP-Atg8 during SD-N
