# Supplementary material for: Phospholipid imbalance impairs autophagosome completion
Source: EMBO J. 2022 Oct 27;41(23):e110771. doi: 10.15252/embj.2022110771 (PMC9713711; doi:10.15252/embj.2022110771)
Supplement: Supplementary file 5 — Movie EV3 [file EMBJ-41-e110771-s002.zip › Movie 3/movie 3.docx]

Movie 3:

Δ*opi3*Δ*ypt7* cells expressing GFP-Atg8 during SD-N
