# Supplementary figures and images for: Phospholipid imbalance impairs autophagosome completion
Source: EMBO J. 2022 Oct 27;41(23):e110771. doi: 10.15252/embj.2022110771 (PMC9713711; doi:10.15252/embj.2022110771)

Anti GFP

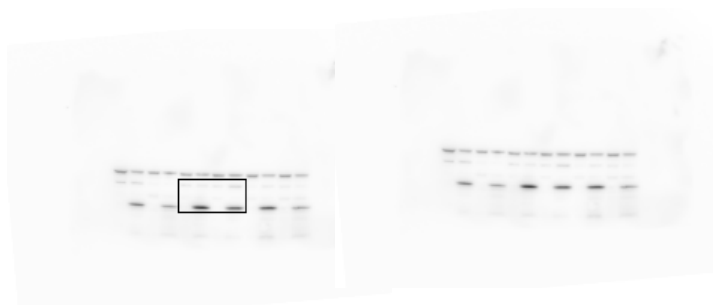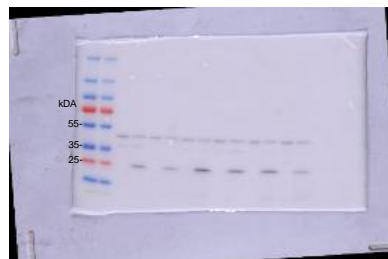

Anti Pgk1

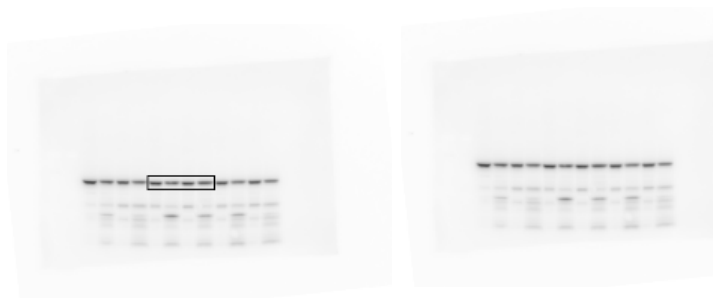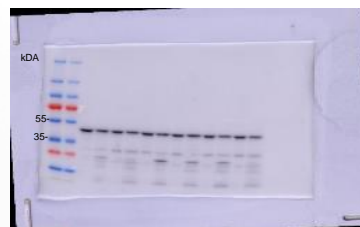

Anti Ape1

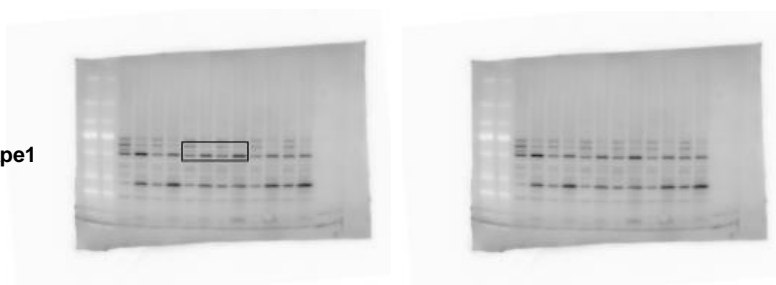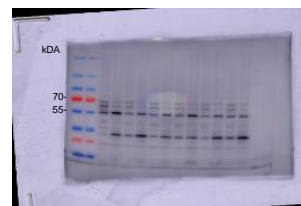

Anti GFP

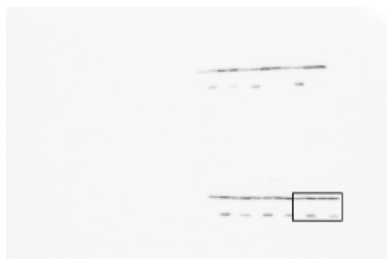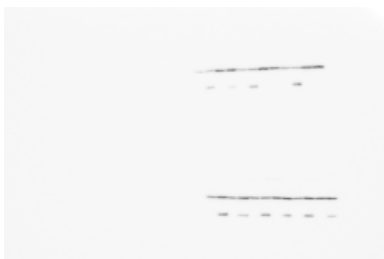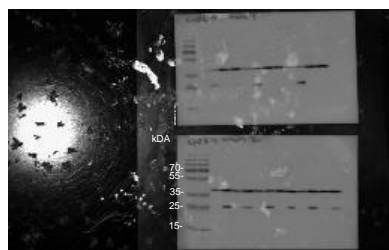

Anti Pgk1

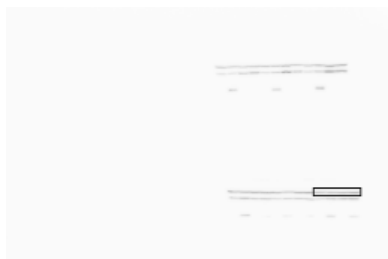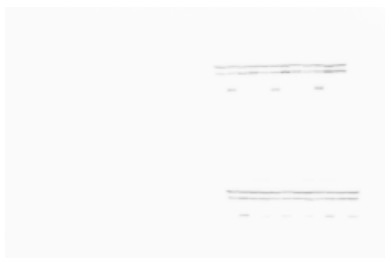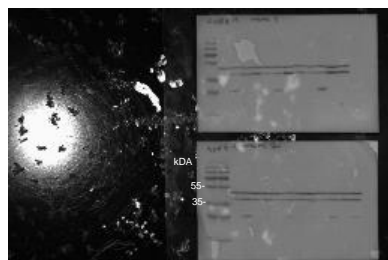

Anti Ape1

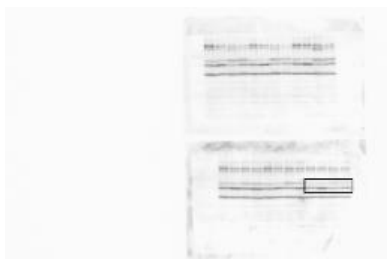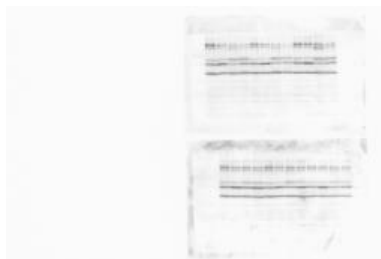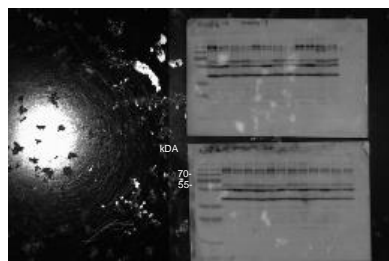

Anti GFP

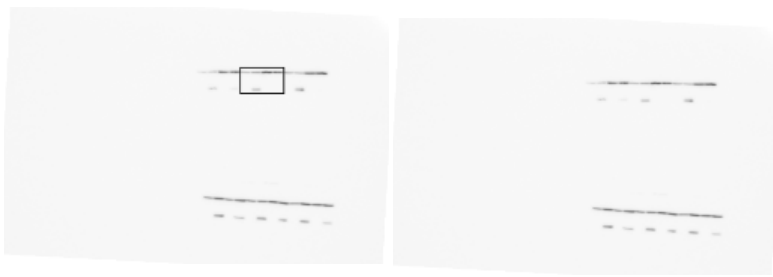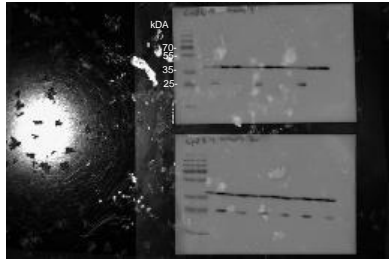

Anti Pgk1

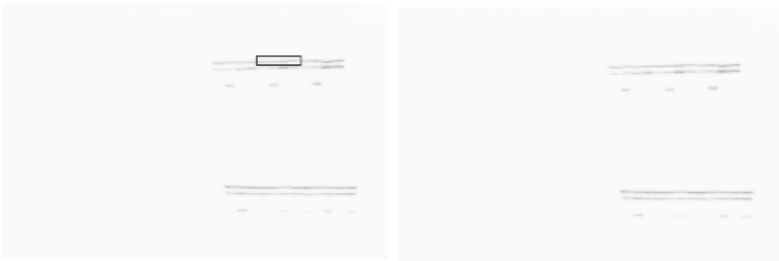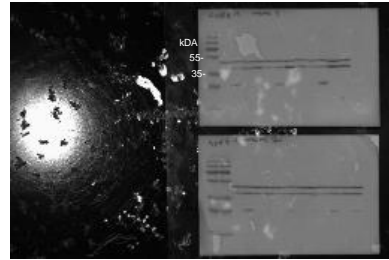

Anti Ape1

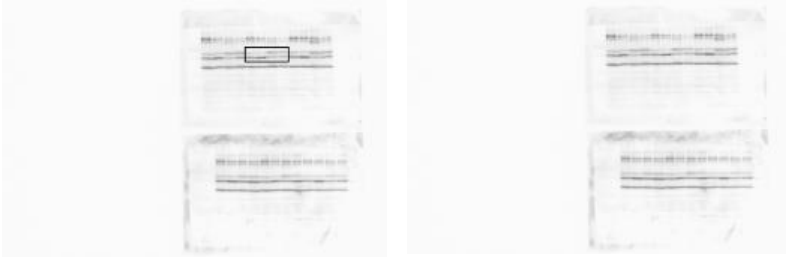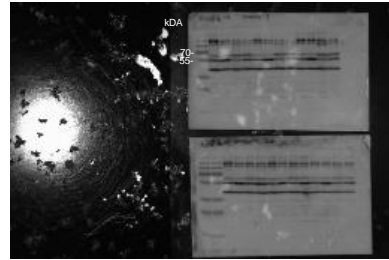

Anti GFP

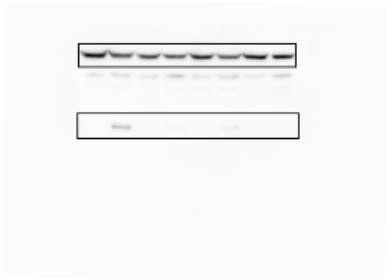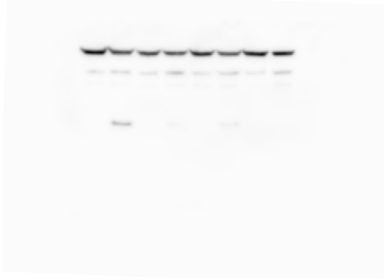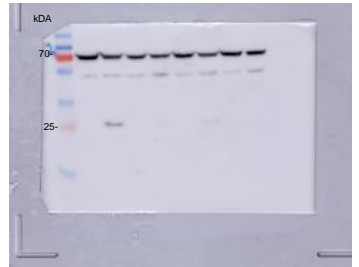

Anti Pgk1

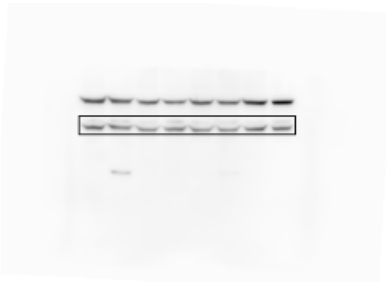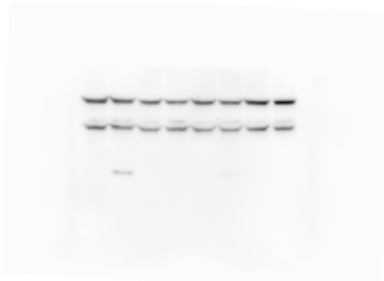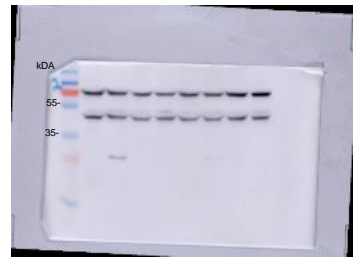

Supplement: Supplementary file 9 — Source Data for Figure 1 [file EMBJ-41-e110771-s009.zip › EMBOJ-2022-110771R1-Figure_1_Source_Data-sd.pdf]

Anti GFP

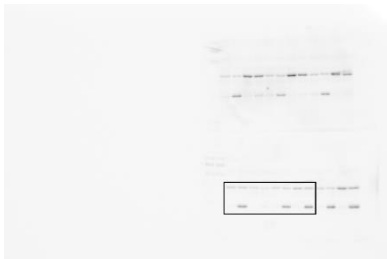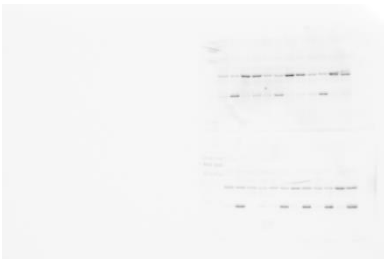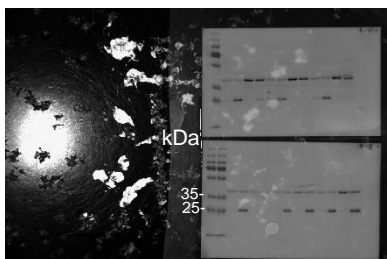

Anti Pgk1

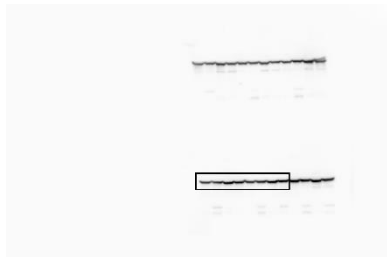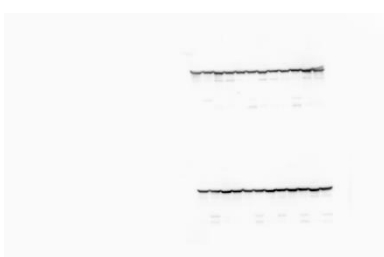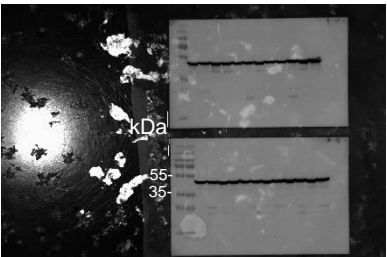

Anti Ape1

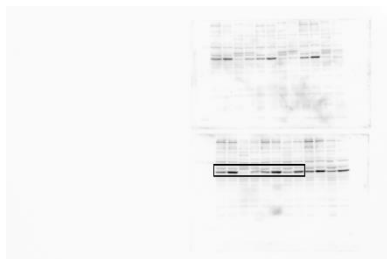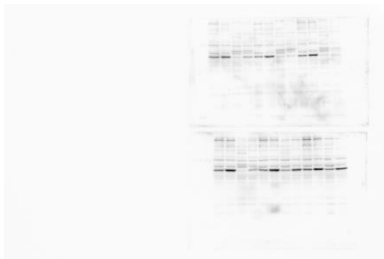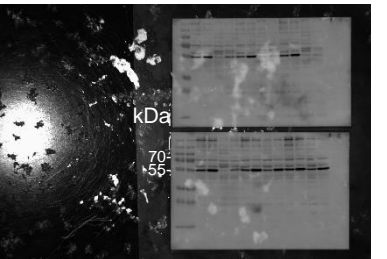

Anti GFP

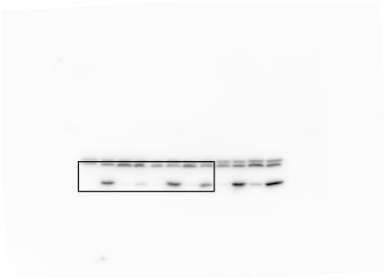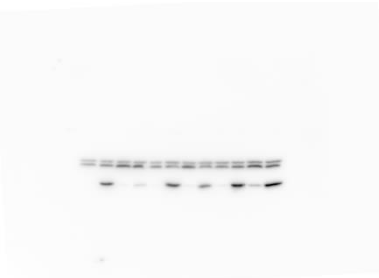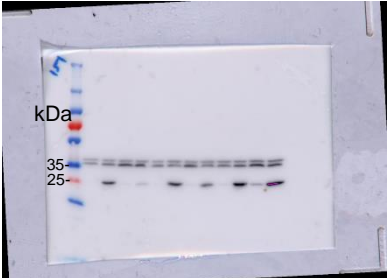

Anti Pgk1

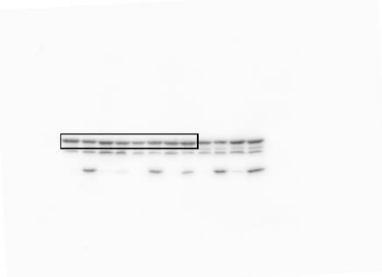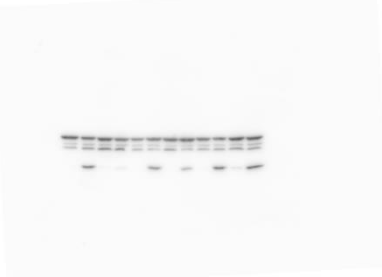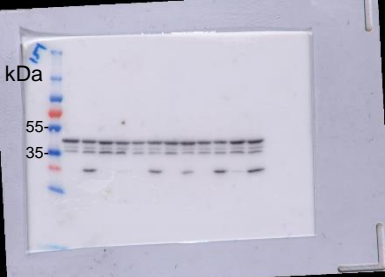

Anti Ape1

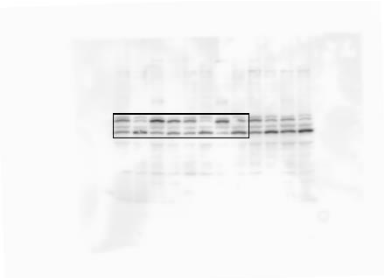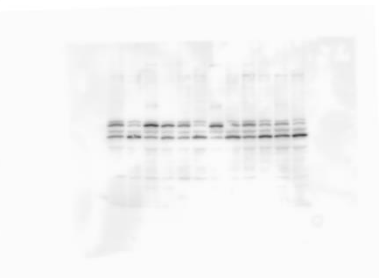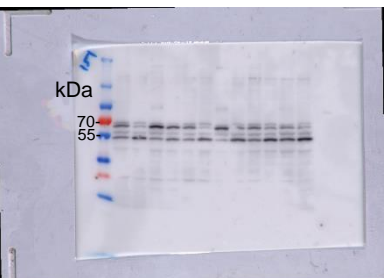

Supplement: Supplementary file 10 — Source Data for Figure 3 [file EMBJ-41-e110771-s011.zip › EMBOJ-2022-110771R1-Figure_3_Source_Data-sd.pdf]
