# Supplementary material for: Phospholipid imbalance impairs autophagosome completion
Source: EMBO J. 2022 Oct 27;41(23):e110771. doi: 10.15252/embj.2022110771 (PMC9713711; doi:10.15252/embj.2022110771)
Supplement: Supplementary file 12 [file EMBJ-41-e110771-s012.pdf]

THE EMBO JOURNAL

Phospholipid imbalance impairs autophagosome completion

Zvulun Elazar, Alexandra Polyansky, Oren Shatz, Milana Fraiberg, Eyal Shimoni, Tali Dadosh, Fulvio Reggiori, Xianlin Han, Muriel Mari, and Chao Qin
DOI: 10.15252/embj.2022110771

Corresponding author(s): Zvulun Elazar (zvulun.elazar@weizmann.ac.il)

Review Timeline:

Submission Date:	25th Jan 22
Editorial Decision:	18th Feb 22
Revision Received:	15th Aug 22
Editorial Decision:	6th Sep 22
Revision Received:	20th Sep 22
Accepted:	22nd Sep 22

Editor: William Teale

Transaction Report:

(Note: With the exception of the correction of typographical or spelling errors that could be a source of ambiguity, letters and reports are not edited. Depending on transfer agreements, referee reports obtained elsewhere may or may not be included in this compilation. Referee reports are anonymous unless the Referee chooses to sign their reports.)

Dear Zevi,

Thank you again for the submission of your manuscript entitled "Phosphatidylcholine levels regulate autophagosome completion". I am really glad you persevered with us and we were able to make some progress on the submission process. We have now received the referees' reports, which I have copied to the bottom of this message.

As you can see, the referees agree that your work clearly demonstrates a role for opi3 in phagophore development. Furthermore, all referees agree that the work is based on a technically accomplished collection of experiments; they also state unambiguously that the manuscript is timely and the topic is important. However, the feedback was not unambiguously positive. Referee 1 expresses three important concerns which will need directly to be addressed; firstly, whether the effect of changes in abundance of other lipids in the phagophore be discounted, secondly, why phagophore growth rates seem less affected than might be expected, and thirdly how the data you present can be reconciled with the recent study published by Schutter et al. (and to a lesser extent the study by Boumann et al. as pointed out by referee 2).

I would, though, like to invite you to address the comments of all referees in a revised version of the manuscript. I recommend we talk next week by Zoom to discuss the referees' comments further and whether the suggested lipidomics experiments are feasible; if you would like to do this, please suggest a couple of time slots. If you have any questions in the meantime, please do not hesitate to write to me.

I should add that it is The EMBO Journal policy to allow only a single major round of revision and that it is therefore important to resolve these concerns at this stage. Please contact me if you have any questions, need further input on the referee comments or if you anticipate any problems in addressing any of their points. Please, follow the instructions below when preparing your manuscript for resubmission.

I would also like to point out that as a matter of policy, competing manuscripts published during this period will not be taken into consideration in our assessment of the novelty presented by your study ("scooping" protection). We have extended this 'scooping protection policy' beyond the usual 3 month revision timeline to cover the period required for a full revision to address the essential experimental issues. Please contact me if you see a paper with related content published elsewhere to discuss the appropriate course of action.

When preparing your letter of response to the referees' comments, please bear in mind that this will form part of the Review Process File, and will therefore be available online to the community. For more details on our Transparent Editorial Process, please visit our website: https://www.embopress.org/page/journal/14602075/authorguide#transparentprocess

Again, please contact me at any time during revision if you need any help or have further questions.

Thank you very much again for the opportunity to consider your work for publication. I look forward to your revision.

Best wishes,

William

William Teale, Ph.D.
Editor
The EMBO Journal

When submitting your revised manuscript, please carefully review the instructions below and include the following items:

1) a .docx formatted version of the manuscript text (including legends for main figures, EV figures and tables). Please make sure that the changes are highlighted to be clearly visible.

2) individual production quality figure files as .eps, .tif, .jpg (one file per figure).

3) a .docx formatted letter INCLUDING the reviewers' reports and your detailed point-by-point response to their comments. As part of the EMBO Press transparent editorial process, the point-by-point response is part of the Review Process File (RPF), which will be published alongside your paper.

4) a complete author checklist, which you can download from our author guidelines (https://wol-prod-cdn.literatumonline.com/pb-assets/embo-site/Author Checklist%20-%20EMBO%20J-1561436015657.xlsx). Please insert information in the checklist that is also reflected in the manuscript. The completed author checklist will also be part of the RPF.

5) Please note that all corresponding authors are required to supply an ORCID ID for their name upon submission of a revised manuscript.

6) We require a 'Data Availability' section after the Materials and Methods. Before submitting your revision, primary datasets produced in this study need to be deposited in an appropriate public database, and the accession numbers and database listed under 'Data Availability'. Please remember to provide a reviewer password if the datasets are not yet public (see https://www.embopress.org/page/journal/14602075/authorguide#datadeposition). If no data deposition in external databases is needed for this paper, please then state in this section: This study includes no data deposited in external repositories. Note that the Data Availability Section is restricted to new primary data that are part of this study.

Note - All links should resolve to a page where the data can be accessed.

7) When assembling figures, please refer to our figure preparation guideline in order to ensure proper formatting and readability in print as well as on screen:
http://bit.ly/EMBOPressFigurePreparationGuideline

Please remember: Digital image enhancement is acceptable practice, as long as it accurately represents the original data and conforms to community standards. If a figure has been subjected to significant electronic manipulation, this must be noted in the figure legend or in the 'Materials and Methods' section. The editors reserve the right to request original versions of figures and the original images that were used to assemble the figure.

8) For data quantification: please specify the name of the statistical test used to generate error bars and P values, the number (n) of independent experiments (specify technical or biological replicates) underlying each data point and the test used to calculate p-values in each figure legend. The figure legends should contain a basic description of n, P and the test applied. Graphs must include a description of the bars and the error bars (s.d., s.e.m.).

9) We would also encourage you to include the source data for figure panels that show essential data. Numerical data can be provided as individual .xls or .csv files (including a tab describing the data). For 'blots' or microscopy, uncropped images should be submitted (using a zip archive or a single pdf per main figure if multiple images need to be supplied for one panel). Additional information on source data and instruction on how to label the files are available at .

10) We replaced Supplementary Information with Expanded View (EV) Figures and Tables that are collapsible/expandable online (see examples in https://www.embopress.org/doi/10.15252/embj.201695874). A maximum of 5 EV Figures can be typeset. EV Figures should be cited as 'Figure EV1, Figure EV2" etc. in the text and their respective legends should be included in the main text after the legends of regular figures.

- For the figures that you do NOT wish to display as Expanded View figures, they should be bundled together with their legends in a single PDF file called *Appendix*, which should start with a short Table of Content. Appendix figures should be referred to in the main text as: "Appendix Figure S1, Appendix Figure S2" etc. See detailed instructions regarding expanded view here: .

- Additional Tables/Datasets should be labelled and referred to as Table EV1, Dataset EV1, etc. Legends have to be provided in a separate tab in case of .xls files. Alternatively, the legend can be supplied as a separate text file (README) and zipped together with the Table/Dataset file.

11) Our journal encourages inclusion of *data citations in the reference list* to directly cite datasets that were re-used and obtained from public databases. Data citations in the article text are distinct from normal bibliographical citations and should directly link to the database records from which the data can be accessed. In the main text, data citations are formatted as follows: "Data ref: Smith et al, 2001" or "Data ref: NCBI Sequence Read Archive PRJNA342805, 2017". In the Reference list, data citations must be labeled with "[DATASET]". A data reference must provide the database name, accession number/identifiers and a resolvable link to the landing page from which the data can be accessed at the end of the reference. Further instructions are available at .

Instructions for preparing your revised manuscript:

Please make sure you upload a letter of response to the referees' comments together with the revised manuscript.

Please also check that the title and abstract of the manuscript are brief, yet explicit, even to non-specialists.

When assembling figures, please refer to our figure preparation guideline in order to ensure proper formatting and readability in

print as well as on screen:
https://bit.ly/EMBOPressFigurePreparationGuideline
See also guidelines for figure legends: https://www.embopress.org/page/journal/14602075/authorguide#figureformat

IMPORTANT: When you send the revision we will require
- a point-by-point response to the referees' comments, with a detailed description of the changes made (as a word file).
- a word file of the manuscript text.
- individual production quality figure files (one file per figure)
- a complete author checklist, which you can download from our author guidelines (https://www.embopress.org/page/journal/14602075/authorguide).
- Expanded View files (replacing Supplementary Information)
Please see out instructions to authors
https://www.embopress.org/page/journal/14602075/authorguide#expandedview

Please remember: Digital image enhancement is acceptable practice, as long as it accurately represents the original data and conforms to community standards. If a figure has been subjected to significant electronic manipulation, this must be noted in the figure legend or in the 'Materials and Methods' section. The editors reserve the right to request original versions of figures and the original images that were used to assemble the figure.

Further information is available in our Guide For Authors: https://www.embopress.org/page/journal/14602075/authorguide

We realize that it is difficult to revise to a specific deadline. In the interest of protecting the conceptual advance provided by the work, we recommend a revision within 3 months (19th May 2022). Please discuss the revision progress ahead of this time with the editor if you require more time to complete the revisions. Use the link below to submit your revision:

https://emboj.msubmit.net/cgi-bin/main.plex

Referee #1:

Review - Phosphatidylcholine levels regulate autophagosome completion
The authors of this manuscript investigated how a change of the cellular lipid composition impacts on the formation of autophagosomes in yeast. They found that deletion of the enzymes involved in the production of PC by the CDP-DAG pathway (Cho2 and Opi3) arrests phagophore formation by inhibiting sealing of phagophores, while deletion of Cpt1/Ept1, which generate PC by the Kennedy pathway, does not inhibit autophagy. Moreover, they found that the Kennedy pathway can compensate for reduced PC levels and thus restore autophagy if an excess of choline is supplemented. Using a series of colocalization and CLEM experiments, the authors showed the in opi3 knock out cells, one enlarged phagophore is formed. The authors concluded that a sealing defect arrests autophagy at a late stage in opi3 knock out cells, preventing the formation of additional autophagosomes, thereby inhibiting autophagic flux.
Overall, the presented data, notably the CLEM image in Fig. 6D, convincingly show that deletion of opi3 impacts on autophagy by impairing phagophore formation. However, several questions remain unanswered. It is, for example, not clear whether the reduction in PC level or the accompanied increase in PI and PS causes the autophagy defect. Furthermore, it is not clear why depletion of lipids impacts on sealing of phagophores (as shown by the authors using giant Ape1 assays), but not on their expansion. Furthermore, the study is in contradiction with a previous study (Schütter et al, Cell, 2020) in which phospholipid biogenesis by Faa1 on phagophores and the Kennedy pathway at the ER contributes to phagophore expansion.
A publication of this study in EMBO would require more mechanistic insights. Moreover, the functional contribution of PC in phagophore sealing needs to be demonstrated. Finally, the contradiction to previous studies needs to be resolved.

Major points:

The formation of the phospholipid precursor DAG at autophagic membranes by Faa1 has been shown to contribute to the formation of autophagosomes by regulating phagophore expansion (Schütter et al, Cell, 2020). DAG would be consumed by the CDP-Choline pathway to generate PC by Cpt1/Ept1. Here, the authors reported that the CDP-Choline pathway does not contribute to autophagy since both, GFP-delivery to the vacuole and Ape1 processing are not impaired in cpt1/ept1 delta cells. How do the authors reconcile this contradiction?

The authors mentioned in their introduction that: "De novo biosynthesis of phospholipids may also contribute to phagophore growth (Andrejeva et al., 2020, Ogasawara et al., 2020, Orii et al., 2021, Schutter et al., 2020). While studies showed that the autophagic membrane is enriched in phosphatidylcholine (PC) (Ogasawara et al., 2020, Orii et al., 2021, Schutter et al., 2020)". However, lipidomic and quantification of PC was only analyzed by Ogasawara et al. and an enrichment of PC in autophagic

membranes was not observed (PC level in log phase total membranes and in autophagic membranes under starvation is similar, ca. 40%). However, the amount of PI increased significantly in total and Atg8 membrane fractions upon starvation and the amount of PE decreased. The authors need demonstrate, using lipidomics, that the amount of PC in autophagic membrane indeed decreases. Lipidomics of total membrane fractions (as provided in Fig 4A) of yeast cells does not permit to draw conclusion of phagophore membrane composition (see differences between total membrane fractions and Atg8 membrane fractions of starved cells in Schutter et al., 2020). Moreover, one would expect that depletion of Opi3 would lead to an increase in PS and PI levels in autophagosomes (as observed already in total membrane fractions by the authors - Fig4A). Since PS and phosphorylation of PI would significantly increase the negative charge of membranes, this could inhibit autophagy as well. The direct proof that a decrease in PC, but not an accompanied increase in PS and PI, lead to autophagy defects thus need to be provided. The observation that addition of choline complements an Opi3 deletion could be explained by the augmented consumption of PA by the Kennedy pathway at the cost of the CDP-DAG pathway, entailing a reduction in PS and PI. To exclude this possibility, the authors should test whether autophagy can be restored in opi3 knock out cells if PA is supplied. The authors could also test whether an excess of ethanolamine impacts on phagophore formation in opi3 knock out cells (which should not be observed). Finally, autophagy in cells with a combined knock out of opi3 and cpt1/ept1 should be investigated.

Conceptional problem: It is difficult to understand that the depletion of PC (if the authors could demonstrate that this is the only significant change in the lipid composition of phagophore membranes in opi3 delta cells) leads to a defect in phagophore sealing and not in phagophore expansion. PC is one of the most common lipids in cells and depleting it should also affect phagophore expansion (as demonstrated by Schutter et al. for the function of FFA on phagophore membranes).

Other comments:

Figure 1B: The bands in 1B are not separated rendering quantification of notably total GFP-levels and Pgk1-levels (loading control) impossible. The authors need to provide gels with well separated bands and repeat, based on optimized and better-quality gels, their quantification.

Figure 1D,E: Deletion of opi3 has a much stronger effect as the deletion of cho2. If PC is required for autophagy and the CDP-DAG pathway is critical to supply PC during starvation, why is deletion of cho2 less severe than deletion of opi3?

Figure 6D: Why do the authors not show a CLEM images of the wt cell (left)?

Figure 7A: The experiment showing that the aberrant Atg8 phenotype in opi3 delta, ypt7 delta cells can be reverted by supplementing choline is interesting. Why do the authors observe one big structure in in opi3 delta, ypt7 delta cells, but many Atg8 puncta in opi3 delta cells in Fig 2A and in Movie 3? CLEM images of opi3 delta, ypt7 delta cells with and w/o choline substitution should be shown. The experiment should also be repeated by complementing PA instead of choline (see above).

Referee #2:

Polyansky et al explore what happens to yeast macroautophagy when the cell is compromised in its ability to produce phosphatidylcholine (PC). Previous studies had implicated PC in supporting mitophagy (PMID 26438722) and its production in playing an essential role in mammalian autophagosome biogenesis (PMID 31517566). The work here supports and/or replicates these stories but significantly, extends the narrative to now show that in yeast, it is specifically the closure of the autophagosome that is disrupted when PC levels are artificially suppressed. They use both fluorescence microscopy to follow the progression of autophagosome growth and beautiful CLEM to confirm that these structures are incomplete. In addition, there is a wealth of lipidomics and general cell biology intended to explore potential secondary consequences in the cell that could have impacted autophagic progression. The work is very well done, several experimental approaches were brought to bear on the principle question and the manuscript was a pleasure to read. I have no major criticisms and expect that this paper will find a broad audience in both the autophagy and lipid homeostasis fields.

My minor concerns relate to the interpretation of some data with respect to previous publications and should each be addressed.
1) **Thoughts on changes to the lipid profile in PC-depleted cells**: The authors do not observe significant overall lipidomic changes in the absence of PC production except for the very significant overproduction of PMME (as expected given the genetic removal of an enzyme functioning between PMME and PC production). In particular, they do not see a change in the acyl chain composition of the zwitterion PE. Previously, in experiments that are fundamentally similar, Boumann et al (PMID 16339082) observed widespread changes in acyl chain composition (both length and saturation) which they interpreted as essential for yeast to maintain a distribution of lipids that could support all forms of membrane dynamics. In particular, the loss of PC was expected to deplete the cell of so-called "cylindrical" lipids and this would need to be compensated by reshaping other lipids in the cell. Could the authors comment on the apparent absence of cylindrical lipids in their model?
2) **Role of Atg8-PMME in autophagosome progression**: In discussing how the only major change in lipid profile is the accumulation of PMME, the authors write: Pg 6 "This attributes the autophagic defects of deltaopi3 to low PC levels rather than to non-specific phospholipid imbalance or a dominant-negative effect of other specific phospholipids". I am not sure this is a fair statement. As noted previously (PMID 26438722), PMME can be used as a substrate in the lipidation of Atg8 proteins, but Atg8-

PMME is not a substrate for the deconjugation reaction driven by Atg4. Thus, under conditions where PMME is very highly overproduced, Atg8-PMME is likely to accumulate. The downstream impact of that accumulation is not entirely clear, but could represent a depletion of available Atg8, or a failure to control a key membrane dynamic event late in autophagosome biogenesis. This sentence should probably be softened, and some discussion around this previously published hypothesis should also be included in their discussion (for example when the highlight this very study, but only comment on the mitophagy effects) as one of the potential explanations for their result.
3) **Morphology of accumulating "phagophores" in PC depleted cells**: Intriguingly, their fluorescence microscopy results in the absence of PC (an accumulation of Atg8 protein into a single bright punctum) is reminiscent of old work on Atg2 knockouts. In that original work, people also speculated around a failure to close the autophagosome, but that interpretation has changed as EM has been brought to bear on the question and it has become apparent that phagophore biogenesis is largely disrupted. Atg2 is a lipid transport protein, so similarities with a lipid homeostasis problem are intriguing. Here the authors show only a single EM image in their main figure (6D,E) which is a compelling example of an unclosed autophagosome. They also quantify the EM, concluding that the presence of phagophores rather than autophagosomes is an all-or-nothing outcome in opi3 vs WT cells. It would be helpful if the authors could clarify their quantification and possibly expand on this result. First, is the quantification in 6D a measure of phagophores from the electron microscopy or of the fluorescence distribution in the images used for the final CLEM (analogous to the quantification in 6C)? It is important to have quantification of the EM itself which is the only place the key conclusion that autophagosomes do not close can be assessed. Second, some discussion of the morphologies observed is warranted. Is the beautiful cup-like structure in 6D/E what is predominantly observed, or do the authors also frequently see vesicles, clusters of vesicles, etc. Indeed, even in some of the fluorescence (particularly with Ape1 OE like 6B) the "phagophore" appears to be a collection of discrete fluorescent puncta. This distinction is again important for evaluating whether the loss of PC is primarily felt at the closure of the autophagosome or earlier in biogenesis.
4) **Accumulation of GFP-Atg8 in PC depleted cells**: Finally, in putting all of these questions together are the very bright puncta observed in opi3 cells or in opi3/ypt7 cells bright because the membrane is so big (as interpreted in final model in figure 7) or because all of the Atg8 is recruited here, perhaps due to Atg8-PMME conjugates representing an irreversible trap of the available protein? Quantification of protein recruitment and of phagophore size seems appropriate to do.

Referee #3:

Formation of a phagophore and its elongation and closure into an autophagosome are critical steps in autophagy. Although the role of PI3P and PE has been studied well, the role of PC, a major phospholipid in eukaryotic cells, in autophagosome formation remains elusive. In this manuscript, the authors studied the role of PC in autophagosome formation using budding yeast. First, the authors showed that the CDP-DAG pathway enzymes, especially Opi3, is important for both selective autophagy (Cvt pathway) and non-selective autophagy. The autophagy defect in delta-opi3 cells was suppressed by addition of Choline and lipidomic analysis revealed that Choline addition recovered the PC level without significantly affecting the composition of the other lipids, suggesting that loss of PC, but not alteration of the other lipid composition, impaired autophagy. Combination of fluorescence microscopy including Airyscan ultra-resolution microscopy and correlative light and electron microscopy showed that phagophores but not autophagosomes were accumulated in delta-opi3 cells. Based on these data, the authors concluded that PC is important for closure of phagophores into autophagosomes.
This is the first report that revealed a critical role of PC in autophagosome formation and contributes to the understanding of basic mechanisms of autophagy. The logic is straightforward, and cell biological data are well supported by lipidomic analysis. Although the molecular mechanism of how PC controls phagophore closure remains elusive, this study gives us the first clue for understanding the critical role of PC in autophagy and thus contributes greatly to the autophagy field. There are some concerns, which should be resolved prior to be published at EMBO Journal.

Major points
1) The most important conclusion of this manuscript is that PC is necessary for closure of phagophores. To further confirm this conclusion, perform a proteinase protection assay, which is often used to judge whether sealed autophagosomes are generated or not using ypt7-deficient cells (for example, Figure 5 in PMID 16079147). Precursor form of Ape1 (prApe1) prepared from delta-opi3 delta-ypt7 cells should be much more severely degraded upon proteinase K treatment compared with those prepared from delta-ypt7 cells if Opi3 is important for closure of phagophores.
2) In Figure 5E, Atg5 signal looks like a dot whereas Atg8 signal looks like an elongated structure. If this structure corresponds to a phagophore, Atg5 should show the same pattern with Atg8. Repeat observation and quantify the frequency of colocalization and pattern difference. If Atg5 repeatedly shows a localization pattern different from Atg8, explain the reason.

Minor points
1) To study the activity of non-selective autophagy quantitatively in budding yeast, Pho8delta60 assay is commonly used (PMID 19185711). Apply this assay to study the importance of Opi3 in non-selective autophagy.
2) Quantify the frequency of observation of cup-shape phagophores in Fig. 6C, EV6A.
3) In page 8, "Fast Airyscan super-resolution microscoy GFP-Atg8-positive cup-shape phagophores in delta-opi3 but not WT cells", this sentence is lacking a verb.
4) Figure 6D has two graphs. What is the difference between them?

We greatly appreciate the helpful and comprehensive reviews we received from the three reviewers and the editor. We invested a considerable effort to address all comments, particularly those related to the determination of the lipid composition of Atg8-labeled phagophore membranes and to extending our CLEM analysis. As part of the revision, we established new strains to further characterize the nature of the Atg8-labeled membranes accumulated in response to PC deficiency. We put most of our effort into isolation of Atg8-labeled membranes from WT and PC-deficient mutants and analysis of their lipid content. This significantly improved our study, solidifying our working hypothesis that changes in phagophore lipid composition caused by PC deficiency leads to the inhibition of the transition from immature open phagophores to mature double membrane autophagosome.

Our point-by-point responses are provided below.

Reviewer #1:

Review - Phosphatidylcholine levels regulate autophagosome completion
The authors of this manuscript investigated how a change of the cellular lipid composition impacts on the formation of autophagosomes in yeast. They found that deletion of the enzymes involved in the production of PC by the CDP-DAG pathway (Cho2 and Opi3) arrests phagophore formation by inhibiting sealing of phagophores, while deletion of Cpt1/Ept1, which generate PC by the Kennedy pathway, does not inhibit autophagy. Moreover, they found that the Kennedy pathway can compensate for reduced PC levels and thus restore autophagy if an excess of choline is supplemented. Using a series of colocalization and CLEM experiments, the authors showed the in opi3 knock out cells, one enlarged phagophore is formed. The authors concluded that a sealing defect arrests autophagy at a late stage in opi3 knock out cells, preventing the formation of additional autophagosomes, thereby inhibiting autophagic flux.
Overall, the presented data, notably the CLEM image in Fig. 6D, convincingly show that deletion of opi3 impacts on autophagy by impairing phagophore formation. However, several questions remain unanswered. It is, for example, not clear whether the reduction in PC level or the accompanied increase in PI and PS causes the autophagy defect.

We thank the reviewer for his constructive comment. We have now performed lipidomic analyses of autophagic (Atg8-positive) membranes, and observed increase in the prominence of PS but not PI (Fig. 4C). The reduction in PC levels is accompanied with alteration in phospholipid composition in the autophagic membranes, and we assume that PS can compensate for low PC levels in the phagophore by similar cylindrical properties in support of planar bilayer surface. Nevertheless, the lipid disequilibrium conferred by low PC levels specifically impairs the capacity of the phagophore to seal – while leaving initiation and elongation intact.

Furthermore, it is not clear why depletion of lipids impacts on sealing of phagophores (as shown by the authors using giant Ape1 assays), but not on their expansion.

As PC deficiency does not impair cell viability and general membrane trafficking (Fig. 2B-D, Fig. EV2B-C), we believe that the majority of flat membrane surfaces – including the majority of the autophagic phagophore surface throughout its expansion – are capable of adapting to the major loss of PC, e.g. by utilizing another cylindrical lipid like PS. We surmise that sealing of the highly-curve phagophore rim demands a more fine-tuned lipid composition.

Of note, as membrane elongation around the Giant Ape1 complex was shown to be aborted half-way through the process in wildtype cells (Suzuki et al., 2013), this assay is not suitable to address sealing. We therefore use it only to show that PC depleted cells are able to maintain phagophore expansion.

Furthermore, the study is in contradiction with a previous study (Schütter et al, Cell, 2020) in which phospholipid biogenesis by Faa1 on phagophores and the Kennedy pathway at the ER contributes to phagophore expansion. A publication of this study in EMBO would require more mechanistic insights. Moreover, the functional contribution of PC in phagophore sealing needs to be demonstrated. Finally, the contradiction to previous studies needs to be resolved.

In the aforementioned study (Schutter et al., 2020), phagophore expansion was assayed by observing the rate of membrane formation along the strictly-selective giant Ape1 complex, where less frequent expansion and a slower growth rate were observed upon loss of localized acyl chain activation and treatment with cerulenin (Fig. 5A-C there). This is attributed to the importance of supply of newly-synthesized fatty acids and consequently phospholipids to the growing phagophore. While our study shows that in conditions where PC level is low (while the supply of newly-synthesized fatty acids is supposed to be intact), the expansion of the phagophore around giant Ape1 cargo is maintained, whereas the sealing of starvation-induced phagophores is compromised. The findings of our study are therefore not in contradiction to the prior publication. Indeed, hours after the begin of starvation-induced autophagy, *opi3* knockout Atg8-positive membranes are visibly well-elongated, yet unsealed as visualized by super-resolution AiryScan microscopy (Fig. 6C, EV6A) and CLEM (Fig. 6D-E, EV6B and Fig. 7F in the revised manuscript), while kinetic analysis shows that sealing is the rate limiting step (Fig. 7A), for successive biogenesis of new autophagosome, thus accounting for low levels of autophagic flux.

It may have been unclear in our original manuscript; however, our data only implies that PC synthesis promotes phagophore closure whereas synthesis of other phospholipids may nevertheless maintain elongation. We clarify this in the discussion section of the revised manuscript. We hope the reviewer agrees that our new lipidomics of the Atg8-positive membranes and our additional data about autophagosome closure and maturation will provide the required insights needed for publication in EMBO J. We nevertheless hope that reviewer agrees that the exact mechanistic reasons by which phospholipid imbalances in the phagophore membrane affect this process will be the subject of future studies. Of note, a recent report by Baumeister and coworkers (https://doi.org/10.1101/2022.05.02.490291), has indicated that the phagophore rim has a complex architecture that may be particularly proem to lipids imbalance, thereby compromising membrane sealing upon PC deficiency but not expansion of the relatively-flat majority of the phagophore surface. We refer to this report in the discussion section of the revised manuscript.

Major points:

The formation of the phospholipid precursor DAG at autophagic membranes by Faa1 has been shown to contribute to the formation of autophagosomes by regulating phagophore expansion (Schütter et al, Cell, 2020). DAG would be consumed by the CDP-Choline pathway to generate PC by Cpt1/Ept1. Here, the authors reported that the CDP-Choline pathway does not contribute to autophagy since both, GFP-delivery to the vacuole and Ape1 processing are not impaired in cpt1/ept1 delta cells. How do the authors reconcile this contradiction?

Faa1, which was studied by (Schutter et al., 2020) in the presence of fatty acid synthetase inhibition by cerulenin, produces acyl-CoA, which is in turn directed to synthesis of lyso-PA, PA and downstream phospholipids – without an involvement of DAG (Fig. 7A there). However, as PA can nevertheless be broken down into DAG by Pah1, the role of DAG in regulation of autophagosome biogenesis remains a valid concern. In this regard, we point that in yeast DAG can be consumed for PC synthesis both directly by Ept1/Cpt1 in the CDP-choline pathway and indirectly by CDP-DAG (DAG->PA->CDP-DAG->PS->PE->PMME->PC), the latter pathway being the primary source for PC in absence of exogenously-provided choline. Indeed, as the Faa1 study was carried out in conditions of choline-deficient synthetic medium, it can be reasonably expected that in that study Acyl-CoA-derived PA was channeled to PC synthesis through CDP-DAG rather than CDP-choline, in line with our reported dispensability of the CDP-choline enzymes Cpt1/Ept1 to autophagy.

The authors mentioned in their introduction that: "De novo biosynthesis of phospholipids may also contribute to phagophore growth (Andrejeva et al., 2020, Ogasawara et al., 2020, Orii et al., 2021, Schutter et al., 2020). While studies showed that the autophagic membrane is enriched in phosphatidylcholine (PC) (Ogasawara et al., 2020, Orii et al., 2021, Schutter et al., 2020)". However, lipidomic and quantification of PC was only analyzed by Ogasawara et al. and an enrichment of PC in autophagic membranes was not observed (PC level in log phase total membranes and in autophagic membranes under starvation is similar, ca. 40%). However, the amount of PI increased significantly in total and Atg8 membrane fractions upon starvation and the amount of PE decreased.

We thank the reviewer for the comment. One of the mentioned studies (Ogasawara et al., 2020) visualized incorporation of newly-synthesized PC into autophagic membranes in mammalian cells, and observed a higher incorporation to autophagosomes than into the outer nuclear membrane (Fig. 2A, B there), suggesting autophagic membranes as a preferable endpoint of newly synthesized PC. In another yeast study (Schutter et al., 2020) the phospholipid composition of total cell membranes in log phase and starvation was compared with that of Atg8 membranes in starvation. In the latter study (Fig. 6A-C there), which is more relevant to ours, total PC was reduced from ~35% in log phase to ~25% in starvation, while PC in starvation-induced Atg8 membranes approached 40% - a 1.6-fold enrichment (40%/25%). In contrast, the PI content in starved Atg8 membranes is slightly less than 40% while that of total membranes is slightly more than 40% – indicating no enrichment. We therefore maintain our statement that PC was shown to be enriched in autophagic membranes.

In the revised manuscript we added the lipidomic analysis of the Atg8-enriched membranes and found that PC is a major component of those membranes, whereas PI is a relatively minor species. This is presented in Figure 4C of the revised manuscript.

The authors need demonstrate, using lipidomics, that the amount of PC in autophagic membrane indeed decreases. Lipidomics of total membrane fractions (as provided in Fig 4A) of yeast cells does not permit to draw conclusion of phagophore membrane composition (see differences between total membrane fractions and Atg8 membrane fractions of starved cells in Schutter et al., 2020). Moreover, one would expect that depletion of Opi3 would lead to an increase in PS and PI levels in autophagosomes (as observed already in total membrane fractions by the authors - Fig4A). Since PS and phosphorylation of PI would significantly increase the negative

charge of membranes, this could inhibit autophagy as well. The direct proof that a decrease in PC, but not an accompanied increase in PS and PI, lead to autophagy defects thus need to be provided.
Conceptional problem: It is difficult to understand that the depletion of PC (if the authors could demonstrate that this is the only significant change in the lipid composition of phagophore membranes in opi3 delta cells) leads to a defect in phagophore sealing and not in phagophore expansion. PC is one of the most common lipids in cells and depleting it should also affect phagophore expansion (as demonstrated by Schutter et al. for the function of FFA on phagophore membranes).

We thank the reviewer for this comment. We now show in a new lipidomic analysis of Atg8-enriched membranes (similar to Schutter et al.,) (Fig. 4C-D, Appendix Fig. S4H-L in the revised manuscript), that autophagic membranes of the *Δopi3* mutant possess low levels of PC, high levels of PS and similar levels of PI.
Choline addition restores PC and to a lesser extend PS levels, while PS acyl chains composition remained different compared to control (Fig. 4C-D, Appendix Fig. S4J in the revised manuscript) – conditions that rescue autophagosome biogenesis and autophagic flux (Fig. 3A-C, Fig. 5C-F, Fig. 7E-F in the revised manuscript). Accordingly, we adjusted our statement to reflect lipid imbalance due to PC deficiency, as possible reason for autophagic impairment, in the revised manuscript.

The observation that addition of choline complements an Opi3 deletion could be explained by the augmented consumption of PA by the Kennedy pathway at the cost of the CDP-DAG pathway, entailing a reduction in PS and PI. To exclude this possibility, the authors should test whether autophagy can be restored in opi3 knock out cells if PA is supplied. The authors could also test whether an excess of ethanolamine impacts on phagophore formation in opi3 knock out cells (which should not be observed).

Our new lipidomic analysis show similar levels of PA and PI with or without choline in Atg8-enriched membranes, and high levels of PS, which acyl chain composition remain different in the presence of choline (Fig. 4C, Appendix SFig. 4J in the revised manuscript). Nevertheless, we did add PA and ethanolamine to WT and *opi3* knockout cells, and saw no influence on autophagic flux, or on the presence of open phagophores as presented below (Figure for the reviewer). We hope that the reviewer will agree with our decision to leave this data out of the revised manuscript as it seems somewhat away from the main focus of the present study.

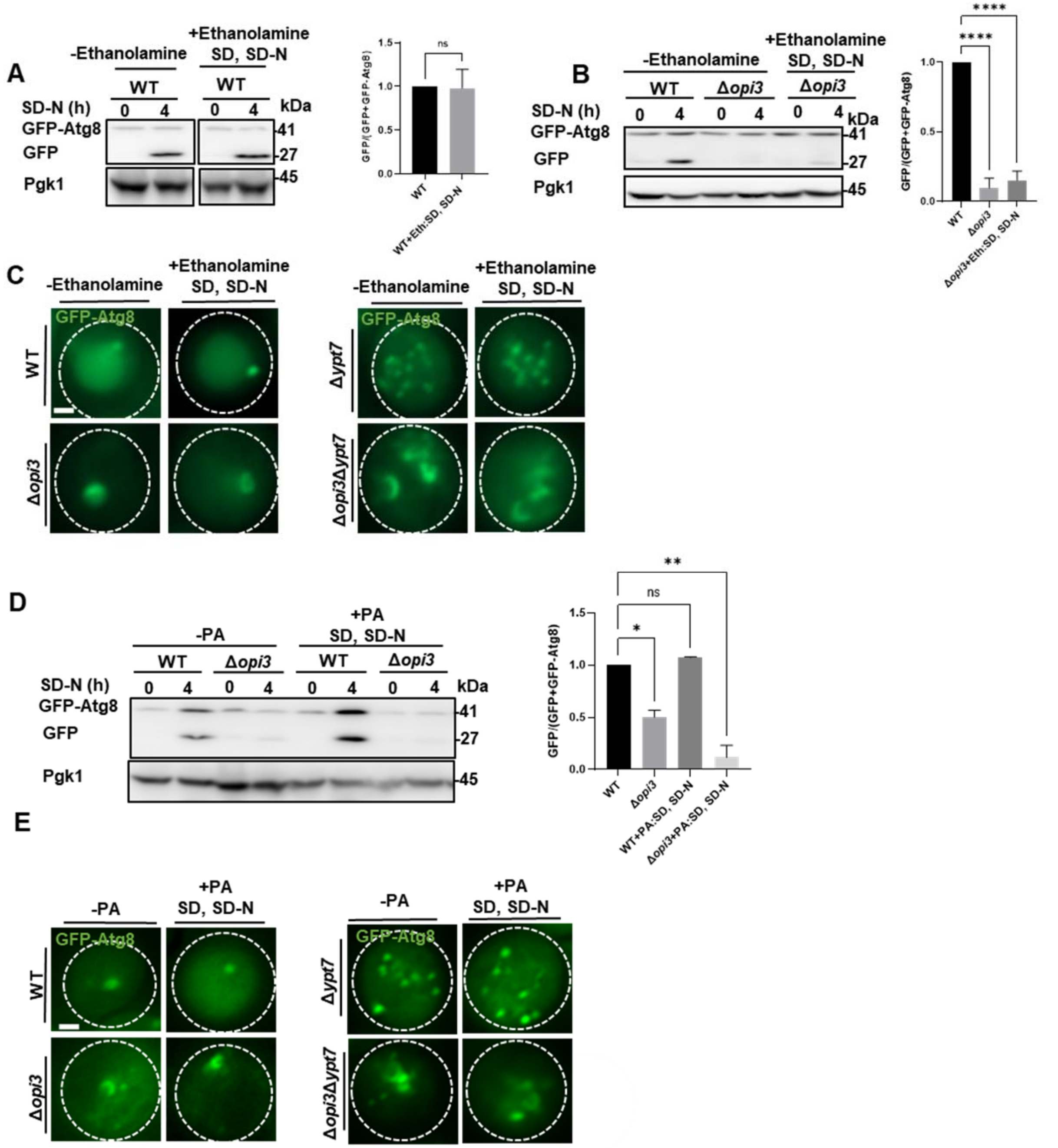

Figure for the reviewers:

Supplementation of ethanolamine and PA does affect autophagy in PC deficient cells

A. WT cells expressing GFP-Atg8 were grown to log phase in SD-URA, and shifted to SD-N for 4 h, ethanolamine (1 mM) was supplemented to SD and SD-N where indicated (+ethanolamine SD, SD-N). Cells were harvested at indicated time points and subjected to western blotting. Pgk1 was monitored as a loading control. Right panel- Autophagic activity was quantified during starvation by calculating the ratio of free GFP to total GFP (GFP-Atg8 + free GFP). Statistical analysis was done by student's t-test (paired, two tailed) (ns- not significant), error bars represent SEM of at least 3 independent experiments.

B. WT and Δ*opi3* cells expressing GFP-Atg8 were grown to log phase in SD-URA, and shifted to SD-N for 4 h, ethanolamine (1 mM) was supplemented to SD and SD-N where indicated (+ethanolamine SD, SD-N). Cells were harvested at indicated time points and subjected to western blotting. Pgk1 was monitored as a loading control. Right panel- Autophagic activity was quantified during starvation by calculating the ratio of free GFP to total GFP (GFP-Atg8 + free GFP). Statistical analysis was done by Anova Dunnett's multiple comparison (****, $p \leq 0.0001$), error bars represent SEM (n=3).
C. Left panel - representative images of WT and Δ*opi3* expressing GFP-Atg8, Cells were grown to log phase in SD-URA medium and starved in SD-N, with supplementation of Ethanolamine (1 mM) to SD and SD-N as indicated. images were taken during starvation (1-3 h) by widefield microscopy. Scale bar 1 μm. Right panel- representative images of Δ*ypt7,* Δ*opi3*Δ*ypt7* expressing GFP-Atg8.. Cells were grown to log phase in SD-URA medium and starved in SD-N with supplementation of Ethanolamine (1 mM) to SD and SD-N as indicated, images were taken during starvation (1-3h) by widefield microscopy (left panel). Scale bar 1 μm.
D. WT and Δ*opi3* cells expressing GFP-Atg8 were grown to log phase in SD-URA, and shifted to SD-N for 4 h, PA (stock solution of 5 mg (3-*sn*-Phosphatidic acid sodium salt from egg yolk lecithin), was dissolved in chloroform, evaporated by nitrogen, and dissolved by 200 μl ethanol and 100 μl chlorophorm, 1:1000 was taken from stock solution) was supplemented to SD and SD-N where indicated (+PA SD, SD-N) and the dissolvent solution without the PA was added to the untreated samples. Cells were harvested at indicated time points and subjected to western blotting. Pgk1 was monitored as a loading control. Right panel- Autophagic activity was quantified during starvation by calculating the ratio of free GFP to total GFP (GFP-Atg8 + free GFP). Statistical analysis was done by Anova Dunnett's multiple comparison (**, $p \leq 0.005$, *, $p \leq 0.001$, ns- not significant), error bars represent SEM (n=3).
E. Left panel - representative images of WT and Δ*opi3* expressing GFP-Atg8. Cells were grown to log phase in SD-URA medium and starved in SD-N, with supplementation of PA to SD and SD-N as indicated. Images were taken during starvation (1-3h) by widefield microscopy (left panel). Scale bar 1 μm.
Right panel- representative images of Δ*ypt7,* Δ*opi3*Δ*ypt7* expressing GFP-Atg8.
Cells were grown to log phase in SD-URA medium and starved in SD-N with supplementation of PA to SD and SD-N as indicated. Images were taken during starvation (1-3h) by widefield microscopy (left panel). Scale bar 1 μm.

Finally, autophagy in cells with a combined knock out of opi3 and cpt1/ept1 should be investigated.

While we agree this is an important control, unfortunately we failed to combine a knockout of *OPI3* with the double mutant of Δ*cpt1*Δ*ept1*, probably due to the lethal absence of PC under this genetic condition.

Other comments:

Figure 1B: The bands in 1B are not separated rendering quantification of notably total GFP-levels and Pgk1-levels (loading control) impossible. The authors need to provide gels with well separated bands and repeat, based on optimized and better-quality gels, their quantification.

We now provide better separated bands and repeated the quantification.

Figure 1D,E: Deletion of opi3 has a much stronger effect as the deletion of cho2. If PC is required for autophagy and the CDP-DAG pathway is critical to supply PC during starvation, why is deletion of cho2 less severe than deletion of opi3?

We thank the reviewer for this comment.
The deletion of *CHO2* leads to a much lesser reduction in PC levels than that of *Δopi3* (Fig. 2E), in correlation with the less severe impairment of autophagy (Fig. 1C-G).

Figure 6D: Why do the authors not show a CLEM images of the wt cell (left)?

Phagophores in our hands are too rare to be detected in CLEM of WT cells. We clarify this in the result section of the revised manuscript.

Figure 7A: The experiment showing that the aberrant Atg8 phenotype in opi3 delta, ypt7 delta cells can be reverted by supplementing choline is interesting. Why do the authors observe one big structure in in opi3 delta, ypt7 delta cells, but many Atg8 puncta in opi3 delta cells in Fig 2A and in Movie 3?

We thank the reviewer for this comment. In the Δ*opi3*Δ*ypt7* double knockout, where vacuolar fusion of autophagosomes is impaired, we mostly observe few large Atg8-positive structures that presumably represent stalled phagophores, (Movie 3 and Fig. 7A, previously Fig. 7B). In contrast, in the fusion-competent *opi3* knockout cells (Fig. 2A) we mostly observe a single perivacuolar structure corresponding to a phagophore.

CLEM images of opi3 delta, ypt7 delta cells with and w/o choline substitution should be shown. The experiment should also be repeated by complementing PA instead of choline (see above).

We thank the reviewer for this comment. We now include a new CLEM analysis with Δ*opi3*Δ*ypt7* double knockout cells with or without choline (Fig. 7F), and show aberrant open phagophores in PC deficient conditions (without choline), which is recovered to only closed autophagosomes upon restoration of PC (by addition of choline). We also added PA and ethanolamine to the Δ*ypt7* stains and saw no difference in the presence of open phagophores (figure for reviewers), and no restoration of the Δ*opi3* impairment back to wildtype-like levels (figure for reviewers).

Reviewer #2:

Polyansky et al explore what happens to yeast macroautophagy when the cell is compromised in its ability to produce phosphatidylcholine (PC). Previous studies had implicated PC in supporting mitophagy (PMID 26438722) and its production in playing an essential role in mammalian autophagosome biogenesis (PMID 31517566). The work here supports and/or replicates these stories but significantly, extends the narrative to now show that in yeast, it is specifically the closure of the autophagosome that is disrupted when PC levels are artificially suppressed. They use both fluorescence microscopy to follow the progression of autophagosome

growth and beautiful CLEM to confirm that these structures are incomplete. In addition, there is a wealth of lipidomics and general cell biology intended to explore potential secondary consequences in the cell that could have impacted autophagic progression. The work is very well done, several experimental approaches were brought to bear on the principle question and the manuscript was a pleasure to read. I have no major criticisms and expect that this paper will find a broad audience in both the autophagy and lipid homeostasis fields.

My minor concerns relate to the interpretation of some data with respect to previous publications and should each be addressed.

1) Thoughts on changes to the lipid profile in PC-depleted cells: The authors do not observe significant overall lipidomic changes in the absence of PC production except for the very significant overproduction of PMME (as expected given the genetic removal of an enzyme functioning between PMME and PC production). In particular, they do not see a change in the acyl chain composition of the zwitterion PE. Previously, in experiments that are fundamentally similar, Boumann et al (PMID 16339082) observed widespread changes in acyl chain composition (both length and saturation) which they interpreted as essential for yeast to maintain a distribution of lipids that could support all forms of membrane dynamics. In particular, the loss of PC was expected to deplete the cell of so-called "cylindrical" lipids and this would need to be compensated by reshaping other lipids in the cell. Could the authors comment on the apparent absence of cylindrical lipids in their model?

We thank the reviewer for this constructive comment. To address the reshaping of the membrane by other lipids, we have conducted a new lipidomic analysis of autophagic membranes and found PS, which is also a cylindrical phospholipid, to be abundant in those membranes (Fig. 4C, Appendix Fig. S4D of the revised manuscript). We assume the combined prominence of PS with the rest of the adjusted lipid composition support the perseverance of the phagophore membrane integrity in PC-depleted conditions.

While we do not have a clear explanation for the difference in the acyl chains observed in our system to those reported by Boumann et al., however, we speculate that these may be attributed to different metabolic scenario of the two systems. Accordingly, our Δ*opi3* null cells were grown in the absence of choline, contained a low level of PC and were analyzed during starvation. In contrast, Boumann et al. diluted their Δ*opi3*Δ*cho2* double knockout cells from choline supplemented medium to choline deficient medium and analyzed the lipid composition following prolonged PC deficient period.

2) Role of Atg8-PMME in autophagosome progression: In discussing how the only major change in lipid profile is the accumulation of PMME, the authors write: Pg 6 "This attributes the autophagic defects of deltaopi3 to low PC levels rather than to non-specific phospholipid imbalance or a dominant-negative effect of other specific phospholipids". I am not sure this is a fair statement. As noted previously (PMID 26438722), PMME can be used as a substrate in the lipidation of Atg8 proteins, but Atg8-PMME is not a substrate for the deconjugation reaction driven by Atg4. Thus, under conditions where PMME is very highly overproduced, Atg8-PMME is likely to accumulate. The downstream impact of that accumulation is not entirely clear, but could represent a depletion of available Atg8, or a failure to control a key membrane dynamic event late in autophagosome biogenesis. This sentence should probably be softened, and some discussion around this previously published hypothesis should also be included in their

discussion (for example when the highlight this very study, but only comment on the mitophagy effects) as one of the potential explanations for their result.

The addition of choline eliminated open phagophores (in Fig. 3, Fig. 5C-F and Fig. 7E, F), while PMME levels were still elevated (Appendix Fig. S4F), supporting the notion that PC rather than PMME is the culprit of impaired closure. We nevertheless soften our statement indicating that the new lipid disequilibrium caused by the PC deficiency may influence membrane sealing, and expanded on the subject in the discussion section.

3) Morphology of accumulating "phagophores" in PC depleted cells: Intriguingly, their fluorescence microscopy results in the absence of PC (an accumulation of Atg8 protein into a single bright punctum) is reminiscent of old work on Atg2 knockouts. In that original work, people also speculated around a failure to close the autophagosome, but that interpretation has changed as EM has been brought to bear on the question and it has become apparent that phagophore biogenesis is largely disrupted. Atg2 is a lipid transport protein, so similarities with a lipid homeostasis problem are intriguing. Here the authors show only a single EM image in their main figure (6D, E) which is a compelling example of an unclosed autophagosome.

We now added to the revised manuscript additional CLEM images of PC deficient cells with open phagophores (Fig. EV6B and Fig. 7F of the revised manuscript). Moreover, we now created new strains with a fluorescent reporter of autophagic PI3P, and show in Fig.7C of the revised manuscript that PC-deficient phagophores are positive for both Atg8 and PI3P, just like their PC-sufficient counterparts, while only Atg8-positive, PI3P-negative, presumably pre-phagophore PAS structures are evident in cognate Δ*atg1* mutants. Additionally, we show that PC-deficient open phagophores co-localize with the cargo complex Ape1 (Fig. EV7C of the revised manuscript).

They also quantify the EM, concluding that the presence of phagophores rather than autophagosomes is an all-or-nothing outcome in opi3 vs WT cells. It would be helpful if the authors could clarify their quantification and possibly expand on this result.

We thank the reviewer for this comment, we now modified the quantification method from the presence of phagophore per field to % of cells with cup-shaped GFP-Atg8 positive structures. Apparentlly, in the PC deficient strain cup-shaped open structures were predominantly represented (Fig. 6D- right panel of the revised manuscript).

First, is the quantification in 6D a measure of phagophores from the electron microscopy or of the fluorescence distribution in the images used for the final CLEM (analogous to the quantification in 6C)?

We now annotated each graph in the revised manuscript. The quantification in Fig. 6D is the CLEM analysis whereas the quantification in Fig. 6C relates to the Airyscan analysis.

It is important to have quantification of the EM itself which is the only place the key conclusion that autophagosomes do not close can be assessed.

We thank the reviewer for the comment. We quantified the CLEM as depicted in Fig. 6D right panel. However, we employed additional methods to demonstrate the presence of phagophores, as follows. First, we show colocalization with Atg5 which is present only on

immature phagophores but not mature autophagosomes (Fig. 5E, Fig. EV5C of the revised manuscript). Second, we performed protection assay where less protected cargo was detected in PC-deficient cells (Fig. EV7A-B). Finally, to assure that we follow with autophagy-related membranes we now provide additional data which show colocalization of vivid phagophores with PI3P (Fig. 7C) and Ape1 (Fig. EV7C).

Second, some discussion of the morphologies observed is warranted. Is the beautiful cup-like structure in 6D/E what is predominantly observed, or do the authors also frequently see vesicles, clusters of vesicles, etc. Indeed, even in some of the fluorescence (particularly with Ape1 OE like 6B) the "phagophore" appears to be a collection of discrete fluorescent puncta. This distinction is again important for evaluating whether the loss of PC is primarily felt at the closure of the autophagosome or earlier in biogenesis.

We thank the reviewer for this constructive comment, we indeed observed mostly cup shaped structures by CLEM, as was counted in the right panel of Fig. 6D. The quantification showed that most of the structures were phagophores, while clusters and vesicles where not detected. Additionally, new CLEM analysis of those structure is now shown in Figs. EV6B and 7F of the revised manuscript, demonstrating the presence of open structures in PC-deficient cells and the rescue by addition of choline where PC is replenished.
We accept the reviewer's comment regarding the appearance of the Atg8-labeled membrane in Fig. 6B. It is important to mention that such fragmentations were also observed in the WT strain. We therefore analyzed the diameters of the longest continued Atg8-labeled membranes found in WT vs the Δ*opi3* mutant and found longer membrane in the PC deficient cells (0.94/0.44 μM in WT and 1/ 0.76 μM in Δ*opi3*) when averaging number of the longest diameter of the structures in the strains were comparable 0.68 μM in WT and 0.74 μM in Δ*opi3* cells. To avoid confusion in the revised manuscript we replaced the image with phagophore that appear unfragmented in the Δ*opi3* cells.

*4) **Accumulation of GFP-Atg8 in PC depleted cells**: Finally, in putting all of these questions together are the very bright puncta observed in opi3 cells or in opi3/ypt7 cells bright because the membrane is so big (as interpreted in final model in figure 7) or because all of the Atg8 is recruited here, perhaps due to Atg8-PMME conjugates representing an irreversible trap of the available protein? Quantification of protein recruitment and of phagophore size seems appropriate to do.*

We thank the reviewer for this important comment. According to our model, Δ*opi3* or Δ*opi3* Δ*ypt7* double mutant accumulate phagophore membrane that are labeled throughout with Atg8 whereas Δ*ypt7* mutant accumulate mature autophagosome that are labeled mainly in the inner membrane while Atg8 on the outer membrane is delipidated by Atg4. This may explain the difference in intensity observed in our mutants. We discuss this in the Discussion section of the revised manuscript. To overcome this issue, we constructed a new Δ*ymr1* knockout strains for assessing the size of autophagic structures stalled at the maturation stage (Cebollero et al., 2012). As depicted in Fig. 7C-D, the diameter of these structures was similar in both PC deficient and sufficient cells.
Finally, to directly address the issue of PMME, we show in Figs. 3, 5 and 7E & F that the addition of choline eliminated open phagophores, while PMME levels remained elevated (Appendix Fig. S4F), supporting the notion that PC rather than PMME is the culprit for impaired closure.

Reviewer #3:

Formation of a phagophore and its elongation and closure into an autophagosome are critical steps in autophagy. Although the role of PI3P and PE has been studied well, the role of PC, a major phospholipid in eukaryotic cells, in autophagosome formation remains elusive. In this manuscript, the authors studied the role of PC in autophagosome formation using budding yeast. First, the authors showed that the CDP-DAG pathway enzymes, especially Opi3, is important for both selective autophagy (Cvt pathway) and non-selective autophagy. The autophagy defect in delta-opi3 cells was suppressed by addition of Choline and lipidomic analysis revealed that Choline addition recovered the PC level without significantly affecting the composition of the other lipids, suggesting that loss of PC, but not alteration of the other lipid composition, impaired autophagy. Combination of fluorescence microscopy including Airyscan ultra-resolution microscopy and correlative light and electron microscopy showed that phagophores but not autophagosomes were accumulated in delta-opi3 cells. Based on these data, the authors concluded that PC is important for closure of phagophores into autophagosomes. This is the first report that revealed a critical role of PC in autophagosome formation and contributes to the understanding of basic mechanisms of autophagy. The logic is straightforward, and cell biological data are well supported by lipidomic analysis. Although the molecular mechanism of how PC controls phagophore closure remains elusive, this study gives us the first clue for understanding the critical role of PC in autophagy and thus contributes greatly to the autophagy field. There are some concerns, which should be resolved prior to be published at EMBO Journal.

Major points
1) The most important conclusion of this manuscript is that PC is necessary for closure of phagophores. To further confirm this conclusion, perform a proteinase protection assay, which is often used to judge whether sealed autophagosomes are generated or not using ypt7-deficient cells (for example, Figure 5 in PMID 16079147). Precursor form of Ape1 (prApe1) prepared from delta-opi3 delta-ypt7 cells should be much more severely degraded upon proteinase K treatment compared with those prepared from delta-ypt7 cells if Opi3 is important for closure of phagophores.

We thank the reviewer for this comment. We have now performed a protection assay of Ape1 and of GFP-Atg8 fusion cargo on a Δ*ypt7* knockout background, as depicted in Fig. EV7A-B of the revised manuscript, less protected cargo was detected in the PC deficient strain.

2) In Figure 5E, Atg5 signal looks like a dot whereas Atg8 signal looks like an elongated structure. If this structure corresponds to a phagophore, Atg5 should show the same pattern with Atg8. Repeat observation and quantify the frequency of colocalization and pattern difference. If Atg5 repeatedly shows a localization pattern different from Atg8, explain the reason.

We thank the reviewer for the comment we quantify the frequency and localization,
We analyzed 889 WT cells, and detected 490 Atg8-labeled structures with 186 Atg5 puncta, which had 100% colocalization with Atg8. In Δ*opi3* we analyzed 383 cells with 382 Atg8 structures and 140 Atg5 puncta which had 100% colocalization with Atg8. We attributed the fact that not all Atg8-labeled structures contained Atg5 in part to its (Atg5) low abundancy.

For better visualization of Atg5 distribution along the phagophores, a new Δ*ymr1* knockout strains, where maturation is impaired (Cebollero et al., 2012). As shown in Fig. EV5C of the revised manuscript Atg5 was mostly found at the phagophore base in PC sufficient and deficient cells. Importantly, our observations are in line with previous reports of starvation-

induced cells where it has been observed as a single dot at the base of the phagophore (Graef et al., 2013), and with recent implication in starvation-induced PAS formation (Harada et al., 2019), while overlap to Atg8 signal has been observed only on giant Ape1 complex-associated phagophores (Suzuki et al., 2013), which is not our current experimental condition.

As for colocalization frequency in this strain, in Δ*ymr1* cells we analyzed 288 cells, and detected 332 Atg8 structures with 64 Atg5 puncta, which had 100% colocalization with Atg8, in Δ*opi3*Δ *ymr1* we analyzed 143 cells with 134 Atg8 structures and 40 Atg5 puncta which had 100% colocalization with Atg8.

We hope the reviewer agree that this information is not essential for the current study as we only use this data to confirm that the Atg8-labeled structures that colocalize with Atg5 represent phagophores.

Minor points

1) To study the activity of non-selective autophagy quantitatively in budding yeast, Pho8delta60 assay is commonly used (PMID 19185711). Apply this assay to study the importance of Opi3 in non-selective autophagy.

The results of this assay are now added to Fig. 1F, of the revised manuscript. This experiment shows the inhibition of non-selective autophagy in PC deficient cells, in line with the data obtained by the Fba1-GFP cleavage assay (Fig. 1E of the revised manuscript).

2) Quantify the frequency of observation of cup-shape phagophores in Fig. 6C, EV6A.

This quantification is now added to Fig. 6C of the revised manuscript. In addition, a similar quantification of such structure in Δ*opi3*Δ*ypt7* double mutant is now added to Fig. 7B of the revised manuscript.

3) In page 8, "Fast Airyscan super-resolution microscoy GFP-Atg8-positive cup-shape phagophores in delta-opi3 but not WT cells", this sentence is lacking a verb.

A verb was added to the sentence.

“ Fast Airyscan super-resolution microscopy detected GFP-Atg8-positive cup-shape structures in Δ*opi3* (but not WT) cells”

4) Figure 6D has two graphs. What is the difference between them?

We thank the reviewer for noticing this. Indeed, in the original submission the graphs were not perfectly aligned with the panels. Accordingly, the upper graph represents the analysis of panel C whereas the lower graph belongs to panel D. This has been corrected in the revised manuscript and annotations were added on top of each graph.

References:

Cebollero E, van der Vaart A, Zhao M, Rieter E, Klionsky DJ, Helms JB, Reggiori F (2012) Phosphatidylinositol-3-phosphate clearance plays a key role in autophagosome completion. *Curr Biol* 22: 1545-53

Graef M, Friedman JR, Graham C, Babu M, Nunnari J (2013) ER exit sites are physical and functional core autophagosome biogenesis components. *Mol Biol Cell* 24: 2918-31

Harada K, Kotani T, Kirisako H, Sakoh-Nakatogawa M, Oikawa Y, Kimura Y, Hirano H, Yamamoto H, Ohsumi Y, Nakatogawa H (2019) Two distinct mechanisms target the autophagy-related E3 complex to the pre-autophagosomal structure. *Elife* 8

Ogasawara Y, Cheng J, Tatematsu T, Uchida M, Murase O, Yoshikawa S, Ohsaki Y, Fujimoto T (2020) Long-term autophagy is sustained by activation of CCTbeta3 on lipid droplets. *Nat Commun* 11: 4480

Schutter M, Giavalisco P, Brodesser S, Graef M (2020) Local Fatty Acid Channeling into Phospholipid Synthesis Drives Phagophore Expansion during Autophagy. *Cell* 180: 135-149 e14

Suzuki K, Akioka M, Kondo-Kakuta C, Yamamoto H, Ohsumi Y (2013) Fine mapping of autophagy-related proteins during autophagosome formation in Saccharomyces cerevisiae. *J Cell Sci* 126: 2534-44

1st Revision - Editorial Decision 6th Sep 2022

Dear Zevi,

We have now received re-review reports from all three referees. Though you have largely addressed the comments of the referees satisfactorily, one referee has remaining concerns, particularly over your firm attribution of a functional role for PC in autophagosome closure. Reviewer 1 maintains that an increase in PS after deletion of Opi3 (if PS would have a negative impact on phagophore closure) could also explain your observations. This reviewer requests, and I agree, that you make modifications to your title, abstract and discussion to account for this possibility. Please also address the other remaining concerns of Reviewer 1.

In addition, there are some remaining editorial points which need to be addressed:
In this regard would you please:

Remove the figures from the manuscript file.
Move Table 1 to after the references and add a short description and title.
Limit the number of keywords to five.
Add a Disclosure and Competing Interests Statement.
Remove figure call-outs to Figures 4G, I, K and L as these panels do not exist.
Zip the movie legends to the corresponding files
Include a table of contents in the appendix file and label as Appendix Figure S1
Please rename the EV figures as follows - EV5 should be EV3, EV6 should be EV4 and EV7 should be EV5 - and update the callouts accordingly.
We encourage the publication of source data, particularly for electrophoretic gels and blots, with the aim of making primary data more accessible and transparent to the reader. It would be great if you could provide me with a PDF file per figure that contains the original, uncropped and unprocessed scans of all or key gels used in the figures. The PDF files should be labeled with the appropriate figure/panel number, and should have molecular weight markers; further annotation could be useful but is not essential. The PDF files will be published online with the article as supplementary "Source Data" files. Source Data can also include Excel tables to accompany your graphs. We anticipate that their inclusion will make your work more discoverable and useable to scientists in the future.

We include a synopsis of the paper (see http://emboj.embopress.org/). Please provide me with a general summary statement and 3-5 bullet points that capture the key findings of the paper.
We also need a summary figure for the synopsis. The size should be 550 wide by [200-400] high (pixels). You can also use something from the figures if that is easier.
EMBO Press is an editorially independent publishing platform for the development of EMBO scientific publications.

Best wishes,

William

https://emboj.msubmit.net/cgi-bin/main.plex

Referee #1:

The authors present a revised version of their study. The authors performed lipidomics of Atg8 positive membranes to address my concern that a decrease in PC, but not an increase in PS or PI induces the autophagy defect in delta opi3 cells. The results of this key experiment demonstrate that deletion of opi3 leads to a decrease in PC levels of Atg8-positive membranes, but also to a concomitant increase in PC levels. Substitution of choline to restore PC levels leads to an increase in PC levels and to a decrease in PS levels. This demonstrates that either depletion of PC from autophagic membranes, or the increase in PS induces the autophagy defect. All data can be interpreted as a consequence of the one or the other effect. The conclusion that reduced levels of PC stall autophagy at the stage of phagophore completion is thus not supported by the data.

Major points:

1) The authors state that: "The reduction in PC levels is accompanied with alteration in phospholipid composition in the autophagic membranes, and we assume that PS can compensate for low PC levels in the phagophore by similar cylindrical properties in support of planar bilayer surface." However, PS is not a cylindrical, but a conical lipid which partitions preferentially into HII and cubic phases. PC is indeed a cylindrical lipid that supports the formation of lamellar phases. Thus, the conclusion that PS can support the formation of lamellar phases and planar bilayer surfaces is not true. By contrast, PS stabilizes negative

membrane curvature which would interfere with the formation of flat membranes. Moreover, PS is a negatively charged lipid and increased levels in PS strongly change the properties of membranes by destabilizing lamellar phases and providing an access of negative charges.
2) The authors tried to address the concern that not reduced PC levels, but a concomitant increase in the levels of PI and PS are causing the autophagy defect by lipidomics of delta opi3 cells with and without substitution of choline. The authors state that "Δopi3 mutant had higher phosphatidylinositol (PI) and PMME levels, which were only partially reduced upon addition of choline". However, the difference of PI levels is significant in the absence, but not significant in the presence of choline upon deletion of opi3, indicating that PI levels are sufficiently restored upon addition of choline. More importantly, the authors performed lipidomic of Atg8 positive membranes to correlate changes in the lipid composition of autophagic membranes with an autophagy defect. Here, the authors found that deletion of opi3 leads to a decrease in PC levels, as predicted, but also to a strong increase in PS levels, which is entirely restored upon addition of choline. Thus, the data do not support the conclusion that loss of PC levels lead to an autophagy defect. Since PS is not only a conical, but also a charged lipid, the increase in PS drastically changes the properties of Atg8 positive membranes. It remains unclear whether the decrease of PC levels or the increase of PS levels inhibits autophagy.
3) The authors analyzed whether inhibition of autophagy in delta opi3 cells occurs at the level of autophagy initiation or expansion of phagophores by comparing recruitment of Atg5 to the PAS. Why do the authors observe formation of Atg8 puncta in delta atg3 cells? One would expect that in the absence of Atg8 conjugation, Atg8 is not recruited to the PAS? Do the authors consider using another marker for the PAS (Atg1 or Atg14, for example)?
4) To verify that expansion of phagophores also remain intact upon opi3 deletion, the authors analyzed the formation of Atg8 positive cup shaped membranes around giant Ape1 cargo in wt and delta opi3 cells. In their quantification, no Atg8-positive cups were detected in WT cells (Fig. 6C), but the authors show such cups in WT cells in Fig 4B. This inconsistency needs to be resolved. Furthermore, data in expanded Fig 6 A suggest that also completed autophagosomes (ring like structures) are present in delta opi3 cells (middle and left panel). The shape of the schematics appears to be an overinterpretation of the fluorescent data.
5) The same limitation applies to data shown in Fig. 7C. The resolution of the fluorescent images does not allow to assign round and "not fully round" shapes to Atg8 positive structures in WT or delta opi3 cells.

Other points:
1) The quality of the blots shown in Fig. 1C needs to be improved. The bands of the different lanes are not well separated and it will thus be difficult to quantify GFP and

Referee #2:

The authors have satisfactorily addressed my original concerns. The possibility that PC is an essential component of autophagosome closure is intriguing and will be of relatively broad interest.

Referee #3:

The authors have addressed all of my concerns.

Dear Zevi,

We have now received re-review reports from all three referees. Though you have largely addressed the comments of the referees satisfactorily, one referee has remaining concerns, particularly over your firm attribution of a functional role for PC in autophagosome closure. Reviewer 1 maintains that an increase in PS after deletion of Opi3 (if PS would have a negative impact on phagophore closure) could also explain your observations. This reviewer requests, and I agree, that you make modifications to your title, abstract and discussion to account for this possibility. Please also address the other remaining concerns of Reviewer 1.

In accordance with the request of reviewer 1 and your suggestion, we now modified the title and text of our manuscript to emphasize the fact that PC deficiency in autophagic membrane is accompanied by aberrant accumulation of PS. We believe our current focus on specific aspects of phospholipid equilibrium with the autophagic membrane will be instrumental in further studies on the mechanism of autophagosome biogenesis.

In addition, there are some remaining editorial points which need to be addressed: In this regard would you please:

Remove the figures from the manuscript file.

The figure legends were moved to the end of the manuscript.

Move Table 1 to after the references and add a short description and title.

Done

Limit the number of keywords to five.

Done

Add a Disclosure and Competing Interests Statement.

Done

Remove figure call-outs to Figures 4G, I, K and L as these panels do not exist.

Done

Zip the movie legends to the corresponding files

Done

Include a table of contents in the appendix file and label as Appendix Figure S1

Done

Please rename the EV figures as follows - EV5 should be EV3, EV6 should be EV4 and EV7 should be EV5 - and update the callouts accordingly.

Done

We encourage the publication of source data, particularly for electrophoretic gels and blots, with the aim of making primary data more accessible and transparent to the reader. It would be great if you could provide me with a PDF file per figure that contains the original, uncropped and unprocessed scans of all or key gels used in the figures. The PDF files should be labeled with the appropriate figure/panel number, and should have molecular weight markers; further annotation could be useful but is not essential. The PDF files will be published online with the article as supplementary "Source Data" files. Source Data can also include Excel tables to accompany your graphs. We anticipate that their inclusion will make your work more discoverable and useable to scientists in the future.

We include a synopsis of the paper (see http://emboj.embopress.org/). Please provide me with a general summary statement and 3-5 bullet points that capture the key findings of the paper.

Done

We also need a summary figure for the synopsis. The size should be 550 wide by [200-400] high (pixels). You can also use something from the figures if that is easier. EMBO Press is an editorially independent publishing platform for the development of EMBO scientific publications.

Done

Referee #1:

The authors present a revised version of their study. The authors performed lipidomics of Atg8 positive membranes to address my concern that a decrease in PC, but not an increase in PS or PI induces the autophagy defect in delta opi3 cells. The results of this key experiment demonstrate that deletion of opi3 leads to a decrease in PC levels of Atg8-positive membranes, but also to a concomitant increase in PC levels. Substitution of choline to restore PC levels leads to an increase in PC levels and to a decrease in PS levels. This demonstrates that either depletion of PC from autophagic membranes, or the increase in PS induces the autophagy defect. All data can be interpreted as a consequence of the one or the other effect. The conclusion that reduced levels of PC stall autophagy at the stage of phagophore completion is thus not supported by the data.

We thank the referee for noticing this and fully agree that the fact that the phagophore membrane accumulates PS upon PC deficiency raises the possibility that either or both phospholipid changes may affect its closure. We adjusted the manuscript text accordingly to reflect these possibilities.

Major points:

1) The authors state that: "The reduction in PC levels is accompanied with alteration in phospholipid composition in the autophagic membranes, and we assume that PS can compensate for low PC levels in the phagophore by similar cylindrical properties in support of planar bilayer surface." However, PS is not a cylindrical, but a conical lipid which partitions preferentially into HII and cubic phases.

We respectfully maintain our original statements for the cylindrical shape of PS under normal physiological conditions (McMahon & Boucrot, 2015; Osman *et al*, 2011). PS only assumes a conical shape under low pH (de Kroon *et al*, 1990; Hope & Cullis, 1980).

PC is indeed a cylindrical lipid that supports the formation of lamellar phases. Thus, the conclusion that PS can support the formation of lamellar phases and planar bilayer surfaces is not true. By contrast, PS stabilizes negative membrane curvature which would interfere with the formation of flat membranes. Moreover, PS is a negatively charged lipid and increased levels in PS strongly change the properties of membranes by destabilizing lamellar phases and providing an access of negative charges.

As for the conical shape of PS – please see above. We agree that the negative charge of the accumulated PS may contribute to the observed defect in phagophore closure. We now discuss this in the revised manuscript.

2) The authors tried to address the concern that not reduced PC levels, but a concomitant increase in the levels of PI and PS are causing the autophagy defect by lipidomics of delta opi3 cells with and without substitution of choline. The authors state that "Δopi3 mutant had higher phosphatidylinositol (PI) and PMME levels, which were only partially reduced upon addition of choline". However, the difference of PI levels is significant in the absence, but not significant in the presence of choline upon deletion of opi3, indicating that PI levels are sufficiently restored upon addition of choline.

In the revised manuscript we attribute the defect in autophagosome completion to imbalanced phospholipids composition within the autophagic membrane. At present our data mainly support PS as the chief culprit phospholipid in accordance to the referee's comment above. We nevertheless agree that other phospholipids can take part in this phenotype.

More importantly, the authors performed lipidomic of Atg8 positive membranes to correlate changes in the lipid composition of autophagic membranes with an autophagy defect. Here, the authors found that deletion of opi3 leads to a decrease in PC levels, as predicted, but also to a strong increase in PS levels, which is entirely restored upon addition of choline. Thus, the data do not support the conclusion that loss of PC levels lead to an autophagy defect. Since PS is not only a conical, but also a charged lipid, the increase in PS drastically changes the properties of Atg8 positive membranes. It remains unclear whether the decrease of PC levels or the increase of PS levels inhibits autophagy.

As indicated above, these issues are now addressed in the revised manuscript.

3) The authors analyzed whether inhibition of autophagy in delta opi3 cells occurs at the level of autophagy initiation or expansion of phagophores by comparing recruitment of Atg5 to the PAS. Why do the authors observe formation of Atg8 puncta in delta atg3 cells? One would expect that in the absence of Atg8 conjugation, Atg8 is not recruited to the PAS?

We thank the referee for this comment. We first verified that these are indeed delta atg3 strains. As a matter of fact, this is evident by the cytosolic diffused Atg8 distribution and the absence from the vacuole (Figure 6A). The Atg8 labeled structure presented in the previous revision of the manuscript is over-representation of a very weak signal. We now present representative images of the same experiment.

Do the authors consider using another marker for the PAS (Atg1 or Atg14, for example)?

Indeed, we used Atg2-mNG and observed similar recruitment kinetics to the PAS. We prefer to keep these data for future studies.

4) To verify that expansion of phagophores also remain intact upon opi3 deletion, the authors analyzed the formation of Atg8 positive cup shaped membranes around giant Ape1 cargo in wt and delta opi3 cells. In their quantification, no Atg8-positive cups were detected in WT cells (Fig. 6C), but the authors show such cups in WT cells in Fig 4B. This inconsistency needs to be resolved.

We apologies for the confusion and note that the quantification in Figure 6C refers to the microscopy Airyscan data, while the giant Ape1 appears without quantification in panel 6B. We clarify this in the revised manuscript.

Furthermore, data in expanded Fig 6 A suggest that also completed autophagosomes (ring like structures) are present in delta opi3 cells (middle and left panel). The shape of the schematics appears to be an overinterpretation of the fluorescent data.

We reanalyzed the images shown in Fig. EV4A of the revised manuscript and found only open structures in delta opi3 cells. We therefore respectfully decided to keep our schematic representation of these structures.

5) The same limitation applies to data shown in Fig. 7C. The resolution of the fluorescent images does not allow to assign round and "not fully round" shapes to Atg8 positive structures in WT or delta opi3 cells.

The main purpose of this experimental setup was to analyze the size of the accumulated autophagic structures in these strains. To address the reviewer's comment we omitted the sentence related to shape of these structures from the result section of the revised manuscript.

Other points:

1) The quality of the blots shown in Fig. 1C needs to be improved. The bands of the different lanes are not well separated and it will thus be difficult to quantify GFP and

While the GFP-Atg8 fusion seems slightly connected, the conclusion from this experiment is supported by the free GFP cleavage and is complemented by the microscopic observation indicating inhibition of GFP-Atg8 entry to the vacuole (Figure 2A). We therefore maintain the figure as it stands.

References

de Kroon AI, Timmermans JW, Killian JA, de Kruijff B (1990) The pH dependence of headgroup and acyl chain structure and dynamics of phosphatidylserine, studied by 2H-NMR. Chem Phys Lipids 54: 33-42

Hope MJ, Cullis PR (1980) Effects of divalent cations and pH on phosphatidylserine model membranes: a 31P NMR study. Biochem Biophys Res Commun 92: 846-852

McMahon HT, Boucrot E (2015) Membrane curvature at a glance. J Cell Sci 128: 1065-1070

Osman C, Voelker DR, Langer T (2011) Making heads or tails of phospholipids in mitochondria. J Cell Biol 192: 7-16

2nd Revision - Editorial Decision 22nd Sep 2022

Dear Zevi,

I am pleased to inform you that your manuscript has been accepted for publication in the EMBO Journal.

Congratulations on a really insightful piece of work!

Referee #1:

The authors successfully addressed my remaining concerns and I recommend publication of this very interesting work in the EMBO Journal.

Please note that it is EMBO Journal policy for the transcript of the editorial process (containing referee reports and your response letter) to be published as an online supplement to each paper. If you do NOT want this, you will need to inform the Editorial Office via email immediately. More information is available here: https://www.embopress.org/page/journal/14602075/authorguide#transparentprocess

Your manuscript will be processed for publication in the journal by EMBO Press. Manuscripts in the PDF and electronic editions of The EMBO Journal will be copy edited, and you will be provided with page proofs prior to publication. Please note that supplementary information is not included in the proofs.

You will be contacted by Wiley Author Services to complete licensing and payment information. The required 'Page Charges Authorization Form' is available here: https://www.embopress.org/pb-assets/embo-site/tej_apc.pdf - please download and complete the form and return to embopressproduction@wiley.com

EMBO Press participates in many Publish and Read agreements that allow authors to publish Open Access with reduced/no publication charges. Check your eligibility: https://authorservices.wiley.com/author-resources/Journal-Authors/open-access/affiliation-policies-payments/index.html

Should you be planning a Press Release on your article, please get in contact with embojournal@wiley.com as early as possible, in order to coordinate publication and release dates.

If you have any questions, please do not hesitate to call or email the Editorial Office. Thank you for your contribution to The EMBO Journal.

Best wishes,

William

William Teale, PhD
Editor
The EMBO Journal
w.teale@embojournal.org

** Click here to be directed to your login page: https://emboj.msubmit.net

EMBO Press Author Checklist

Corresponding Author Name: Zvulun Elazar	
Journal Submitted to: EMBO J	
Manuscript Number: EMBOJ-2022-110771	

USEFUL LINKS FOR COMPLETING THIS FORM
The EMBO Journal - Author Guidelines
EMBO Reports - Author Guidelines
Molecular Systems Biology - Author Guidelines
EMBO Molecular Medicine - Author Guidelines

Reporting Checklist for Life Science Articles (updated January
This checklist is adapted from Materials Design Analysis Reporting (MDAR) Checklist for Authors. MDAR establishes a minimum set of requirements in transparent reporting in the life sciences (see Statement of Task: 10.31222/osf.io/9sm4x). Please follow the journal's guidelines in preparing your
Please note that a copy of this checklist will be published alongside your article.

Abridged guidelines for figures
1. Data
The data shown in figures should satisfy the following conditions:
- the data were obtained and processed according to the field's best practice and are presented to reflect the results of the experiments in an accurate and unbiased manner.
- ideally, figure panels should include only measurements that are directly comparable to each other and obtained with the same assay.
- plots include clearly labeled error bars for independent experiments and sample sizes. Unless justified, error bars should not be shown for technical
- if n<5, the individual data points from each experiment should be plotted. Any statistical test employed should be justified.
- Source Data should be included to report the data underlying figures according to the guidelines set out in the authorship guidelines on Data

2. Captions
Each figure caption should contain the following information, for each panel where they are relevant:
- a specification of the experimental system investigated (eg cell line, species name).
- the assay(s) and method(s) used to carry out the reported observations and measurements.
- an explicit mention of the biological and chemical entity(ies) that are being measured.
- an explicit mention of the biological and chemical entity(ies) that are altered/varied/perturbed in a controlled manner.
- the exact sample size (n) for each experimental group/condition, given as a number, not a range;
- a description of the sample collection allowing the reader to understand whether the samples represent technical or biological replicates (including how many animals, litters, cultures, etc.).
- a statement of how many times the experiment shown was independently replicated in the laboratory.
- definitions of statistical methods and measures:
  - common tests, such as t-test (please specify whether paired vs. unpaired), simple χ2 tests, Wilcoxon and Mann-Whitney tests, can be unambiguously identified by name only, but more complex techniques should be described in the methods section;
  - are tests one-sided or two-sided?
  - are there adjustments for multiple comparisons?
  - exact statistical test results, e.g., P values = x but not P values < x;
  - definition of 'center values' as median or average;
  - definition of error bars as s.d. or s.e.m.

Please complete ALL of the questions below.
Select "Not Applicable" only when the requested information is not relevant for your study.

Materials

Newly Created Materials	Information included in the manuscript?	In which section is the information available? (Reagents and Tools Table, Materials and Methods, Figures, Data Availability Section)
New materials and reagents need to be available; do any restrictions apply?	Yes	New yeast strains - described in the Material and Method Section

Antibodies	Information included in the manuscript?	In which section is the information available? (Reagents and Tools Table, Materials and Methods, Figures, Data Availability Section)
For **antibodies** provide the following information:<br>- Commercial antibodies: RRID (if possible) or supplier name, catalogue number and or/clone number<br>- Non-commercial: RRID or citation	Yes	The information is avaliable in the Material and Method Section

DNA and RNA sequences	Information included in the manuscript?	In which section is the information available? (Reagents and Tools Table, Materials and Methods, Figures, Data Availability Section)
**Short novel DNA or RNA including primers, probes**: provide the sequences.	Not Applicable	

Cell materials	Information included in the manuscript?	In which section is the information available? (Reagents and Tools Table, Materials and Methods, Figures, Data Availability Section)
**Cell lines:** Provide species information, strain. Provide accession number in repository **OR** supplier name, catalog number, clone number, and/**OR** RRID.	Not Applicable	
**Primary cultures:** Provide species, strain, sex of origin, genetic modification status.	Not Applicable	
Report if the cell lines were recently **authenticated** (e.g., by STR profiling) and tested for mycoplasma contamination.	Not Applicable	

Experimental animals	Information included in the manuscript?	In which section is the information available? (Reagents and Tools Table, Materials and Methods, Figures, Data Availability Section)
**Laboratory animals or Model organisms:** Provide species, strain, sex, age, genetic modification status. Provide accession number in repository **OR** supplier name, catalog number, clone number, **OR** RRID.	Not Applicable	
**Animal observed in or captured from the field:** Provide species, sex, and age where possible.	Not Applicable	
Please detail **housing and husbandry conditions**.	Not Applicable	

Plants and microbes	Information included in the manuscript?	In which section is the information available? (Reagents and Tools Table, Materials and Methods, Figures, Data Availability Section)
**Plants:** provide species and strain, ecotype and cultivar where relevant, unique accession number if available, and source (including location for collected wild specimens).	Not Applicable	
**Microbes:** provide species and strain, unique accession number if available, and source.	Not Applicable	

Human research participants	Information included in the manuscript?	In which section is the information available? (Reagents and Tools Table, Materials and Methods, Figures, Data Availability Section)
If collected and within the bounds of privacy constraints report on age, sex and gender or ethnicity for all study participants.	Not Applicable	

Core facilities	Information included in the manuscript?	In which section is the information available? (Reagents and Tools Table, Materials and Methods, Figures, Data Availability Section)
If your work benefited from core facilities, was their service mentioned in the acknowledgments section?	Not Applicable	

Design

Study protocol	Information included in the manuscript?	In which section is the information available? (Reagents and Tools Table, Materials and Methods, Figures, Data Availability Section)
If study protocol has been **pre-registered, provide DOI in the manuscript**. For clinical trials, provide the trial registration number **OR** cite DOI.	Not Applicable	
Report the **clinical trial registration number** (at ClinicalTrials.gov or equivalent), where applicable.	Not Applicable	

Laboratory protocol	Information included in the manuscript?	In which section is the information available? (Reagents and Tools Table, Materials and Methods, Figures, Data Availability Section)
Provide DOI OR other citation details if **external detailed step-by-step protocols** are available.	Not Applicable	

Experimental study design and statistics	Information included in the manuscript?	In which section is the information available? (Reagents and Tools Table, Materials and Methods, Figures, Data Availability Section)
Include a statement about **sample size** estimate even if no statistical methods were used.	Yes	Throught the manuscript
Were any steps taken to minimize the effects of subjective bias when allocating animals/samples to treatment (e.g. **randomization procedure**)? If yes, have they been described?	Not Applicable	
Include a statement about **blinding** even if no blinding was done.	Not Applicable	
Describe **inclusion/exclusion criteria** if samples or animals were excluded from the analysis. Were the criteria pre-established?<br><br>If sample or data points were omitted from analysis, report if this was due to attrition or intentional exclusion and provide justification.	Not Applicable	
For every figure, are **statistical tests** justified as appropriate? Do the data meet the assumptions of the tests (e.g., normal distribution)? Describe any methods used to assess it. Is there an estimate of variation within each group of data? Is the variance similar between the groups that are being statistically compared?	Yes	Details in the manuscript figure legends, and material and methods

Sample definition and in-laboratory replication	Information included in the manuscript?	In which section is the information available? (Reagents and Tools Table, Materials and Methods, Figures, Data Availability Section)
In the figure legends: state number of times the experiment was **replicated** in laboratory.	Yes	Throughout the manuscript
In the figure legends: define whether data describe **technical or biological replicates**.	Yes	Throughout the manuscript

Ethics

Ethics	Information included in the manuscript?	In which section is the information available? (Reagents and Tools Table, Materials and Methods, Figures, Data Availability Section)
Studies involving **human participants**: State details of **authority granting ethics approval** (IRB or equivalent committee(s), provide reference number for approval.	Not Applicable	
Studies involving **human participants**: Include a statement confirming that **informed consent** was obtained from all subjects and that the experiments conformed to the principles set out in the WMA Declaration of Helsinki and the Department of Health and Human Services Belmont Report.	Not Applicable	
Studies involving **human participants:** For publication of **patient photos**, include a statement confirming that consent to publish was obtained.	Not Applicable	
Studies involving experimental **animals**: State details of **authority granting ethics approva**l (IRB or equivalent committee(s), provide reference number for approval. Include a statement of compliance with ethical regulations.	Not Applicable	
Studies involving **specimen and field samples:** State if relevant **permits** obtained, provide details of authority approving study; if none were required, explain why.	Not Applicable	

Dual Use Research of Concern (DURC)	Information included in the manuscript?	In which section is the information available? (Reagents and Tools Table, Materials and Methods, Figures, Data Availability Section)
Could your study fall under dual use research restrictions? Please check biosecurity documents and list of **select agents and toxins** (CDC): https://www.selectagents.gov/sat/list.htm	Not Applicable	
If you used a select agent, is the security level of the lab appropriate and reported in the manuscript?	Not Applicable	
If a study is subject to dual use research of concern regulations, is the name of the **authority granting approval and reference number** for the regulatory approval provided in the manuscript?	Not Applicable	

Reporting

The MDAR framework recommends adoption of discipline-specific guidelines, established and endorsed through community initiatives. Journals have their own policy about requiring specific guidelines and recommendations to complement MDAR.

Adherence to community standards	Information included in the manuscript?	In which section is the information available? (Reagents and Tools Table, Materials and Methods, Figures, Data Availability Section)
State if relevant guidelines or checklists (e.g., **ICMJE, MIBBI, ARRIVE, PRISMA**) have been followed or provided.	Not Applicable	
For **tumor marker prognostic studies,** we recommend that you follow the **REMARK** reporting guidelines (see link list at top right). See author guidelines, under 'Reporting Guidelines'. Please confirm you have followed these guidelines.	Not Applicable	
For **phase II and III randomized controlled trials**, please refer to the **CONSORT** flow diagram (see link list at top right) and submit the CONSORT checklist (see link list at top right) with your submission. See author guidelines, under 'Reporting Guidelines'. Please confirm you have submitted this list.	Not Applicable	

Data Availability

Data availability	Information included in the manuscript?	In which section is the information available? (Reagents and Tools Table, Materials and Methods, Figures, Data Availability Section)
Have **primary datasets** been deposited according to the journal's guidelines (see 'Data Deposition' section) and the respective accession numbers provided in the Data Availability Section?	Not Applicable	
Were **human clinical and genomic datasets** deposited in a public access-controlled repository in accordance to ethical obligations to the patients and to the applicable consent agreement?	Not Applicable	
Are **computational models** that are central and integral to a study available without restrictions in a machine-readable form? Were the relevant accession numbers or links provided?	Not Applicable	
If publicly available data were reused, provide the respective **data citations in the reference list**.	Not Applicable	
