# Supplementary material for: Phospholipid imbalance impairs autophagosome completion
Source: EMBO J. 2022 Oct 27;41(23):e110771. doi: 10.15252/embj.2022110771 (PMC9713711; doi:10.15252/embj.2022110771)
Supplement: Supplementary file 8 — Source Data for Expanded View [file EMBJ-41-e110771-s001.zip › EMBOJ-2022-110771R1-Source_Data_for_EV2-sd.zip!/EMBOJ-2022-110771R1-Source_Data_for_EV2-sd.pdf]

Anti CPY

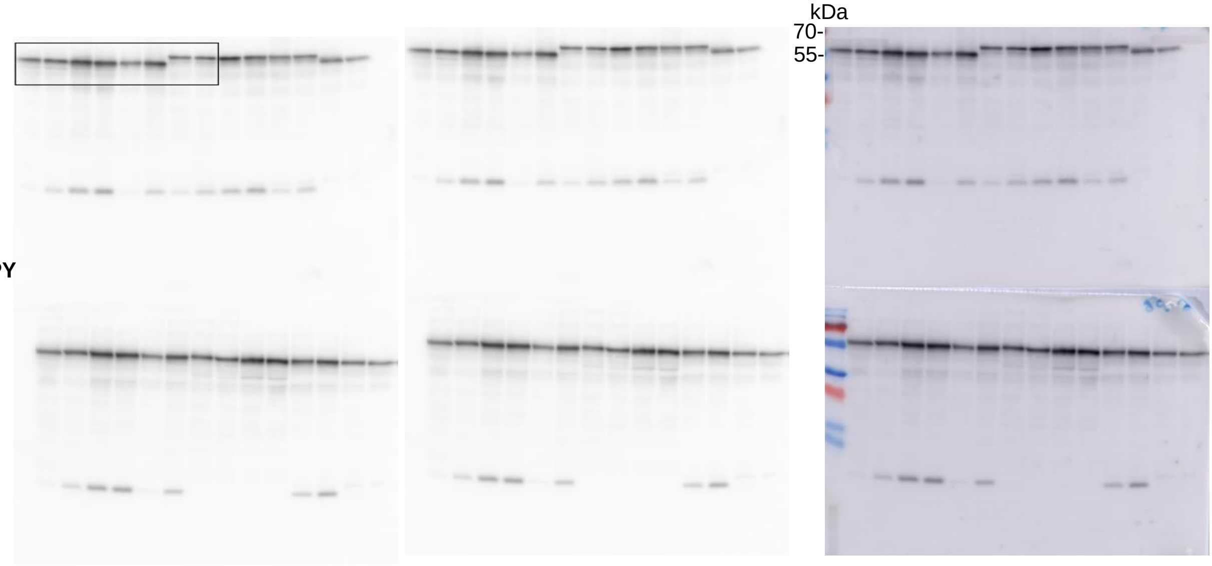

Anti Pgk1

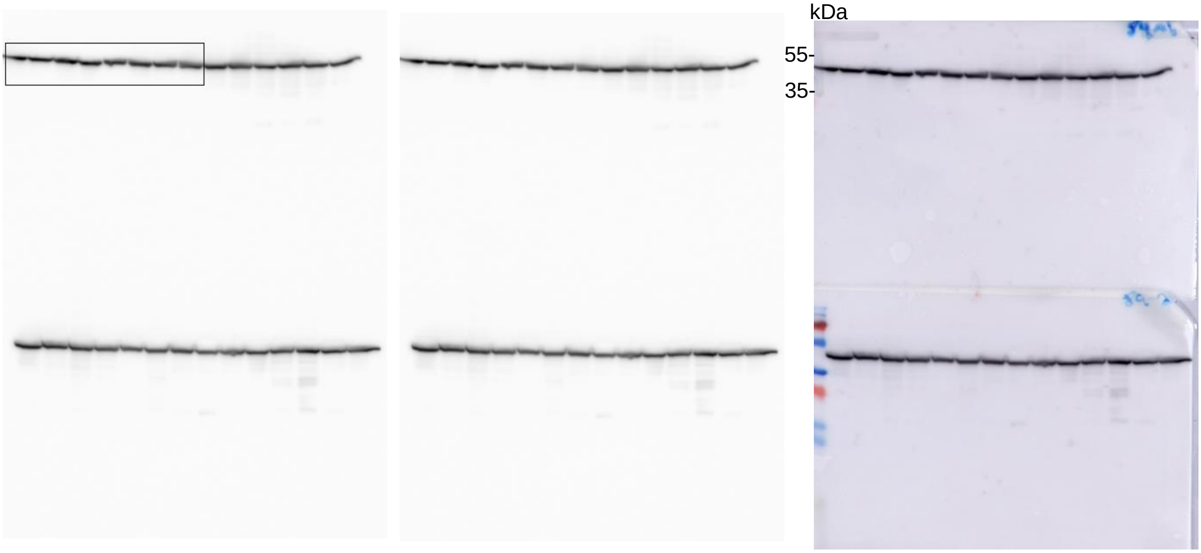

Source data Fig. EV2B
